# Assembly-dependent translational feedback regulation of photosynthetic proteins in land plants

**DOI:** 10.1038/s41477-025-02074-x

**Published:** 2025-08-18

**Authors:** Rabea Ghandour, Yang Gao, Stephanie Ruf, Ralph Bock, Reimo Zoschke

**Affiliations:** 1 Max Planck Institute of Molecular Plant Physiology, Potsdam-Golm, Germany; 2 Present Address: Institute of Microbiology, Friedrich Schiller University, Jena, Germany

**Keywords:** Plant molecular biology, Plant physiology

## Abstract

In the green alga *Chlamydomonas reinhardtii*, the synthesis of chloroplast-encoded photosynthetic subunits is feedback regulated by their protein complex assembly state. This regulation is known as control by epistasy of synthesis (CES) and matches subunit synthesis with requirements of complex assembly in photosystem II (PSII), the cytochrome *b*_6_*f* complex (Cyt *b*_*6*_*f*), photosystem I (PSI), ATP synthase and Rubisco. In embryophytes, CES was only described for Rubisco, raising the question of whether CES exists for components of the photosynthetic electron transport chain in land plants. Here we systematically examined land plant mutants with assembly defects in PSII, Cyt *b*_*6*_*f*, PSI, ATP synthase, NAD(P)H dehydrogenase-like (NDH) complex and Rubisco for feedback regulation. We confirmed the CES in Rubisco and provide evidence for translational feedback regulation in PSII, involving *psbA*, *psbB* and *psbD*, and in Cyt *b*_*6*_*f*, connecting *petA* and *petB*. Our results also point to potential feedback regulation between ATP synthase subunits. Most of these regulatory connections are not conserved between algae and embryophytes. We did not find evidence for CES in land plant PSI or NDH complex assembly. Our results, however, indicate a regulatory connection between PSII and PSI. Overall, we revealed commonalities and differences in assembly-dependent feedback regulation of photosynthetic complexes between embryophytes and green algae.

## Abstract (web summary)

Algae fine-tune protein synthesis to assembly demands of photosynthetic complexes. In land plants, this feedback control is conserved in Rubisco and partly in photosystem II, but varies or is absent in other complexes, revealing divergent evolution.

## Main

Photosynthesis provides the basis for life on earth by producing organic compounds and molecular oxygen. In plants and eukaryotic algae, photosynthesis occurs in chloroplasts, specialized organelles that descend from cyanobacteria^1,2^. Water-splitting and the light-driven electron transport reactions of photosynthesis as well as ATP synthesis are executed by thylakoid membrane-embedded multisubunit protein complexes. The genes that encode the subunits of these complexes and the carbon-fixing enzyme Rubisco are distributed between the nuclear and chloroplast genomes^3,4^. This bipartite genomic arrangement requires intercompartmentally coordinated gene expression to ensure matched subunit synthesis and unobstructed assembly. The cross-compartmental orchestration of gene expression is enabled by bidirectional signalling between the nucleus and chloroplasts^5^. Many photosynthetic core subunits are chloroplast encoded, and it was suggested that the evolutionary preservation of these genes within plastomes was driven by requirements of organelle-autonomous feedback regulation^6,7^.

Across domains of life, steady-state protein synthesis of the subunits of multiprotein complexes is stoichiometrically adjusted to ensure controlled complex assembly^8–10^. For instance, in chloroplasts of the green alga Chlamydomonas (*Chlamydomonas reinhardtii*) and the embryophytes tobacco (*Nicotiana tabacum*), Arabidopsis (*Arabidopsis thaliana*) and maize (*Zea mays*), the non-equimolar stoichiometry of ATP synthase subunits is preset by accordingly tuned levels of gene expression^11–13^. The subunits of other photosynthetic membrane protein complexes, namely: photosystem I and II (PSI and PSII), the cytochrome *b*_6_*f* complex (Cyt *b*_6_*f*) and the NAD(P)H dehydrogenase-like (NDH) complex, as well as those of the stromal Rubisco are assembled in an equimolar stoichiometry. To reveal whether assembly-dependent feedback regulation contributes to the adjusted accumulation of the subunits in photosynthetic complexes, targeted perturbations in the production of core subunits and simultaneous examination of the synthesis of their assembly partners are required. Indeed, such approaches were broadly applied in Chlamydomonas and revealed negative autoregulatory feedback regulation at the translational level (termed control by epistasy of synthesis, CES) within each of the abovementioned photosynthetic complexes^9^ (Supplementary Fig. 1). In PSII, for instance, interconnected assembly-dependent regulation controls the protein synthesis of the two core subunits PsbA (D1) and PsbB (CP47) as follows: PsbD (D2) accumulation controls the synthesis of PsbA, which sets the level of PsbB synthesis^14^. Similar CES cascades are controlled by the assembly status of Cyt *b*_6_*f* (linking PetB, PetD and PetA)^15^, PSI (involving PsaB, PsaA and PsaC)^16^, ATP synthase (including ATPC, AtpB and AtpA)^17^ and Rubisco (connecting RBCS and RbcL)^18,19^. Hence, CES is a well-established and widespread feature in the expression of chloroplast-encoded photosynthetic genes in Chlamydomonas.

Little is known about the prevalence of assembly-dependent translational feedback regulation in embryophytes, the clade that contains all seed plants. Similar to Chlamydomonas, in tobacco, synthesis of the Rubisco large subunit (RbcL) is translationally feedback-controlled by the availability of the small subunit (RBCS)^18,20^. It was also speculated that the Chlamydomonas CES of PetA (cytochrome *f*) within the Cyt *b*_6_*f* complex is conserved in embryophytes^15,21,22^. In addition, a recent model for photodamage-induced autoregulation of PsbA synthesis in embryophytes may be applicable to the de novo assembly of PSII^23,24^. Finally, the analysis of ATP synthase mutants in embryophytes^12,25^ did not reveal the regulatory link between AtpB and AtpA synthesis that had been previously described in Chlamydomonas^17^. Overall, despite the identified CES in Chlamydomonas, analyses to prove or disprove the existence of similar regulatory feedback loops in embryophytes are scarce and currently inconclusive.

To get a more complete picture of potential CES mechanisms in embryophytes, we here validated the suitability of ribosome profiling to detect the known CES of Rubisco in a tobacco *RBCS* mutant. Next, we systematically examined adequate tobacco and Arabidopsis mutants with impaired assembly of PSII, Cyt *b*_6_*f*, PSI, NDH complex and ATP synthase to broadly survey for assembly-dependent feedback regulation in embryophyte chloroplasts. Our results indicate that synthesis of the PSII subunits PsbA and PsbB directly depends on the accumulation of PsbD, a translational feedback control that is similar but not identical to that described in Chlamydomonas. Furthermore, we found that, within Cyt *b*_6_*f*, *petA* overexpression stimulates PetB synthesis, a regulatory link that has not been described in Chlamydomonas. In addition, our results may point to feedback regulation in the ATP synthase of embryophytes, revealing potential regulatory connections that are different from the known CES in the ATP synthase of Chlamydomonas. Our results do not provide evidence for translational feedback regulation in PSI or the NDH complex of embryophytes. Finally, our findings suggest a translational control of the PSI core subunits PsaA, PsaB and to some extent also PsaC in response to PSII complex accumulation, thereby providing evidence for a translational regulatory mechanism that adjusts PSII:PSI stoichiometry. Overall, we pinpoint commonalities and differences in assembly-dependent feedback regulation of photosynthetic complexes between embryophytes and green algae, and substantially expand the evolutionary picture of regulatory events involved in the biogenesis of photosynthetic complexes.

## Results

### Systematic search for feedback regulation in embryophytes

To systematically survey for feedback regulation in the assembly of photosynthetic complexes of embryophytes, various tobacco and Arabidopsis mutants with defects in the assembly of photosynthetic complexes were selected for re-examination by ribosome and transcriptome profiling analyses (Supplementary Table 1). To this end, we used the following criteria to choose adequate mutants: (1) In view of the laborious generation and initial characterization of novel transgenic lines (especially transplastomic lines), we mostly considered mutants that were readily available (as seeds or in tissue culture) and have been previously extensively characterized (Supplementary Table 1). This approach greatly enhanced the efficiency of our survey, thereby enabling the parallel examination of all photosynthetic complexes in a decent time. On the other hand, previously generated mutants were not specifically designed to survey for CES. Hence, some of these mutants do not provide completely unambiguous results (which we carefully discuss). (2) On the basis of the known CES in Chlamydomonas and the available information about the ordered assembly of photosynthetic complexes, we mainly chose lines with altered expression of core subunits in PSII, Cyt *b*_6_*f*, PSI, ATP synthase and the NDH complex. (3) In the past, embryophyte mutants of nucleus-encoded chloroplast-targeted RNA-binding proteins that control the expression of chloroplast photosynthetic genes were analysed to gain insights into chloroplast feedback regulation (for example, ref. ^25^). However, these proteins (often PPR proteins) commonly serve more than one target RNA (for example, ref. ^26^). Hence, the interpretation of results can be ambiguous in that additional targets may be misinterpreted as feedback-regulated genes. Therefore, whenever available, transplastomic tobacco lines were chosen that either knock out or knock down the expression of specific photosynthetic core subunits by directly disrupting reading frames or mutating well-defined *cis* elements of gene expression (for example, promoters, start codons or Shine–Dalgarno sequences). To accelerate the survey for CES in land plants even further, we also considered lines that knock out several photosynthetic core subunits in parallel, thereby allowing the time-efficient simultaneous testing for CES effects of diverse subunits (sometimes even in different photosynthetic complexes). An obvious drawback of this approach is that the interpretation of results from these lines is not always unambiguous and consequently, we commonly validated interesting results by the analysis of independent mutant lines or alleles. (4) In Chlamydomonas, knockout alleles of photosynthetic core subunits often enabled the detection of even mild feedback regulation in mixotrophic growth conditions^9^. However, in land plants, a complete knockout of photosynthesis has a multitude of secondary effects on plant metabolism and development, and requires artificial heterotrophic growth on sucrose-containing medium. Hence, we also considered hypomorphic mutant alleles with residual expression levels that suffice for autotrophic growth. In each of these cases, we determined expression levels of the respective photosynthetic genes with appropriate experiments.

**Extended Data Fig. 1:**
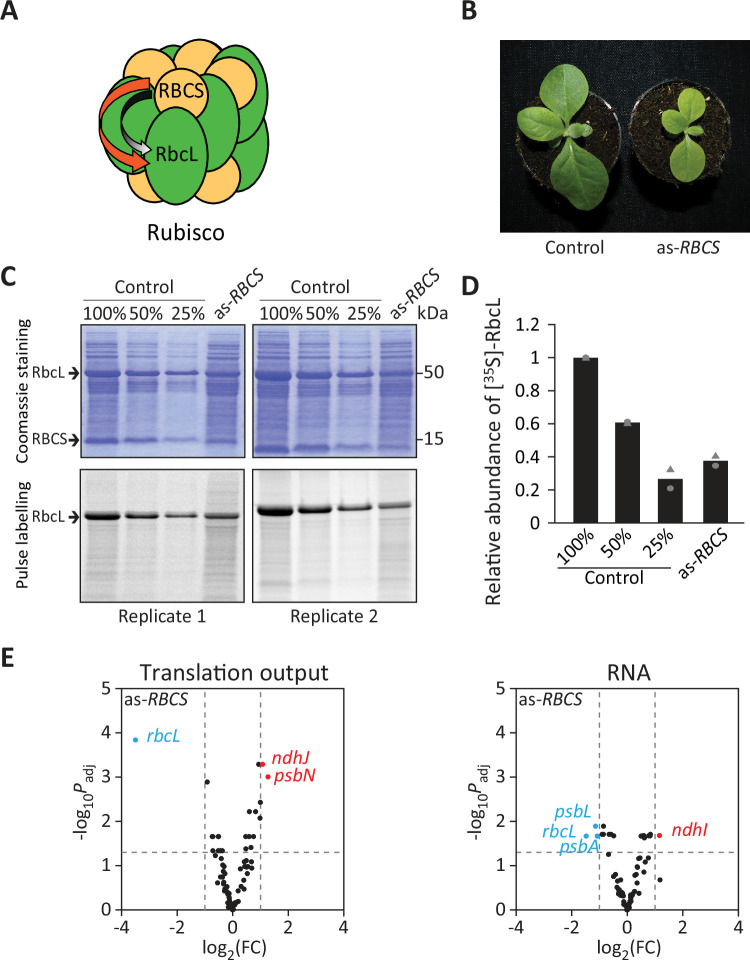
Reduced levels of RBCS provoke a pronounced decrease in the translation output of *rbcL.* (**a**) Scheme of the oligomeric Rubisco complex in the chloroplast. Plastid-encoded and nucleus-encoded subunits are shown in green and yellow, respectively. The black and red arrows connect the assembly partner (arrow origin) to the CES subunit (arrowhead) as reported in Chlamydomonas and tobacco, respectively^18–20^, and verified in this work. The scheme is adapted from Choquet and Wollman^9^. (**b**) The antisense RBCS mutant (as-*RBCS*) analyzed in this work was created by transforming *Nicotiana tabacum* Petit Havana cv. SR1 plants with an *RBCS* antisense DNA construct (Supplementary Table 1). Untransformed SR1 plants were used as control. While SR1 displayed a wild-type phenotype, as-*RBCS* plants were pale green. (**c**) *In vivo* pulse labeling of the SR1 control and as-*RBCS* demonstrated reduced RbcL synthesis in as-*RBCS* and thereby validated the CES regulation of *rbcL*. The experiment was carried out in two biological replicates that displayed similar results. Top panel: Image of the Coomassie staining of all proteins, RbcL and RBCS are marked. Bottom panel: Image of the autoradiograph of the newly synthesized stromal proteins, RbcL is marked. (**d**) Background-normalized quantification of the autoradiograph shown in *C* using Image Lab (Bio-Rad). Dots and triangles represent the data from two biological replicates used for average calculation. (**e**) Ribosome and transcriptome profiling analyses carried out in three biological replicates. Log_2_-fold changes of translation output (left) and transcript abundance (right) between mutant and control plants were plotted in volcano plots against the negative common logarithm of the adjusted *P* values (False Discovery Rate (FDR))^86^ for all chloroplast reading frames (data shown in Supplementary Data 1). Genes whose expression is at least 2-fold downregulated or upregulated with a *P*_adj_ ≤ 0.05 are labelled in blue or red, respectively. The vertical and horizontal dashed lines mark the 2-fold change and the *P*_adj_ of 0.05, respectively. The cuto-ff of 2-fold changes is justified by the 5^th^ percentile in the distribution of fold changes across all microarray data (Supplementary Fig. 2). Relative translation efficiencies, that is changes in translation output that have been normalized to those in transcript accumulation, are presented in Supplementary Fig. 3 and Supplementary Data 1. Source data

As a general proof of concept, we confirmed CES in the tobacco Rubisco complex^18–20^ (Supplementary Results, Extended Data Fig. 1 and Supplementary Data 1). To this end, we analysed a tobacco *RBCS* knockdown mutant and validated the translational feedback regulation of *rbcL* by protein pulse labelling and ribosome profiling experiments (Extended Data Fig. 1). Ribosome profiling takes advantage of messenger (m)RNA fragments that are protected by translating ribosomes against ribonucleases (ribosome footprints). The approach utilizes these ribosome footprints as readout to determine translation output (that is, ribosome coverage of reading frames). Because each ribosome footprint derives from a translating ribosome, translation output provides a genome-wide proxy of protein synthesis levels. The parallel analysis of transcript levels (RNA) enables the differentiation of translation and transcript-level control of translation output. Normalizing changes in translation output to those in transcript accumulation determines the translation efficiency of mRNAs (Supplementary Fig. 3 and Supplementary Data 1). By confirming the known CES in tobacco Rubisco assembly with ribosome profiling (Supplementary Results, Extended Data Fig. 1 and Supplementary Data 1), we validated the suitability of the approach to detect assembly-dependent feedback regulation in land plant chloroplasts.

### Translational feedback regulation in PSII assembly

**Fig. 1:**
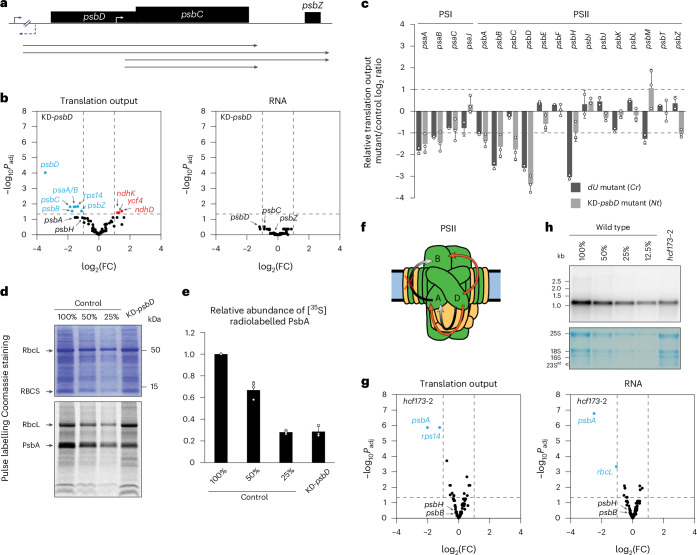
PSII assembly-dependent feedback control of translation in land plants differs from that in Chlamydomonas. **a**, Physical map of the *psbD/C*-coding region in the tobacco *psbD* knockdown mutant. Transcripts that derive from two transcription initiation sites in this polycistron are shown below the map. The selectable marker *aadA* is inserted upstream of *psbD* in antisense direction (dashed arrow). Accumulation of *psbD* transcript derived from the upstream promoter (black arrow) is reduced by the *aadA* insertion. Transcription from an additional internal promoter (white arrow) generates transcripts that largely exclude *psbD*. Genes above the line are transcribed from left to right. Black boxes, chloroplast protein-coding regions; dashed arrow, insertion site and transcription direction of the *aadA* cassette. **b**, Comparison of translation output and transcript accumulation of all chloroplast reading frames in the KD-*psbD* PSII mutant represented in volcano plots (mutant name indicated in the upper-left corner). Results from three biological replicates were summarized and log_2_ fold changes (FC) were plotted against the negative common logarithm of the adjusted *P* values (*P*_adj_) in comparison to an *aadA* cassette-containing control line (Supplementary Table 1; *P* values shown in Supplementary Data 1). Genes whose expression is at least 2-fold downregulated or upregulated with *P*_adj_ ≤ 0.05 are labelled in blue or red, respectively. The vertical and horizontal dashed lines represent cut-offs of 2-fold change and *P*_adj_ = 0.05, respectively. The cut-off of 2-fold changes is justified by the 5th percentile in the distribution of fold changes across all microarray data (Supplementary Fig. 2). Relative translation efficiencies, that is, changes in translation output that have been normalized to those in transcript accumulation, are presented in Supplementary Fig. 3 and Supplementary Data 1. **c**, Mean ± s.d. translation output of PSI and PSII genes in the tobacco (*Nt*) KD-*psbD* mutant (cotyledon stage) and the Chlamydomonas (*Cr*) *dU* mutant grown at 80 µmol photons m^−2^ s^−1^ light intensity^12^ relative to their corresponding controls is highly similar. *n* = 3 biological replicates shown as dots. **d**, Pulse labelling of plastid-encoded proteins demonstrates a decrease in PsbA synthesis due to reduced *psbD* expression. Coomassie staining and autoradiograph of a representative gel are shown (biological replicates are presented in Supplementary Fig. 6). RbcL and PsbA bands are marked. **e**, For three biological replicates, the PsbA signal intensity was quantified using Image Lab software (Bio-Rad) and normalized to the background signal excluding RbcL (thereby, any effect of unequal loading or different labelling efficiency was excluded from the analysis). Data are mean ± s.d.; data points of replicates are shown as dots. **f**, Schematic representation of the PSII complex. Plastid-encoded subunits are shown in green; nucleus-encoded subunits are shown in yellow. Black arrows show the PsbD-PsbA-PsbB CES cascade reported in Chlamydomonas (proteins labelled D, A and B, respectively). In tobacco, the verified PsbD-PsbA CES and the newly discovered PsbD-PsbB CES are represented by red arrows. The PsbA-PsbB CES interaction from Chlamydomonas is not conserved in tobacco (depicted by a red cross). The scheme is adapted from ref. ^9^. **g**, Comparison of translation output and transcript accumulation of all chloroplast reading frames in the *hcf173*-2 PSII mutant (details as in **b**). **h**, RNA gel blot analysis with a *psbA* probe confirms the severe *psbA* transcript accumulation defect observed in the *hcf173*-2 transcriptome profiling data (shown in **g**; RNA ladder sizes are labelled on the left side of the gel; 2 µg RNA is loaded as 100%). Methylene blue staining is shown as a loading control and reflects the abundance of the rRNA species that are labelled on the left. Biological replicates 2 and 3 are shown in Supplementary Fig. 5a. Source data

**Fig. 2:**
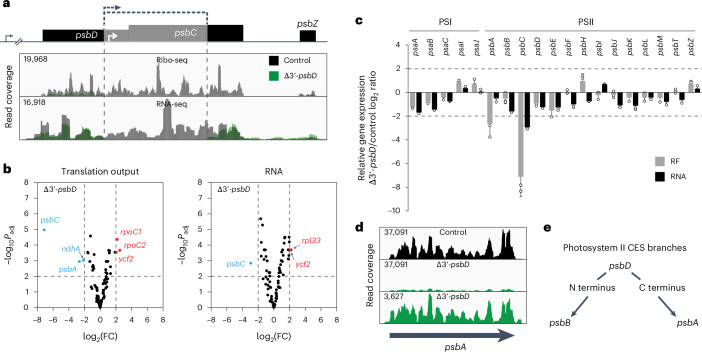
PSII assembly in land plants is probably controlled by a branched CES pathway. **a**, Top: physical map of the Δ3’-*psbD* mutant, in which the 3’ end of *psbD* and a substantial fraction of the 5’ end of *psbC* are replaced by the *aadA* cassette. The deleted region is shaded. Labelling as in Fig. 1a. Bottom: screenshots from Integrative Genomics Viewer (IGV) showing the ribosome footprint and RNA coverage at the *aadA* insertion site in the mutant (green) and the control (grey). The *y* axes represent the number of reads. The maximum *y*-axis values are shown in the upper-left corner. **b**, Volcano plot comparison of the translation output (Ribo-seq) and the transcript abundance (RNA-seq) between Δ3’-*psbD* and *aadA* control line (Supplementary Table 1) for all chloroplast reading frames. Data were collected from three biological replicates and log_2_(FC) of normalized RPKM values were plotted against the negative common logarithm of the adjusted *P* values (*P* values shown in Supplementary Data 1). Genes with at least 4-fold downregulation or upregulation and a *P*_adj_ ≤ 0.01 are labelled in blue or red, respectively. The vertical and horizontal dashed lines represent cut-offs of 4-fold change and *P*_adj_ = 0.01, respectively. The cut-off of 4-fold changes is justified by the 5th percentile in the distribution of fold changes across all NGS data (Supplementary Fig. 2). Translation efficiencies are shown in Supplementary Fig. 3 and Supplementary Data 1. **c**, Mean ± s.d. relative translation output (RF) and RNA accumulation of chloroplast-encoded PSI and PSII genes in Δ3’-*psbD*. *n* = 3 biological replicates shown as dots. **d**, Screenshots from IGV showing the ribosome footprint distribution along the *psbA* reading frame in Δ3’-*psbD* and the control. Note that the mutant data are shown in two different *y*-axis scales to visualize the overall reduction in *psbA* ribosome coverage compared to the control as well as the unaltered distribution of residual ribosomes on the *psbA* reading frame. Other details as in **a**. **e**, Schematic representation showing the proposed branched CES between PSII subunits in embryophytes based on our data (for details, see ‘Results’ and ‘Discussion’).

**Extended Data Fig. 2:**
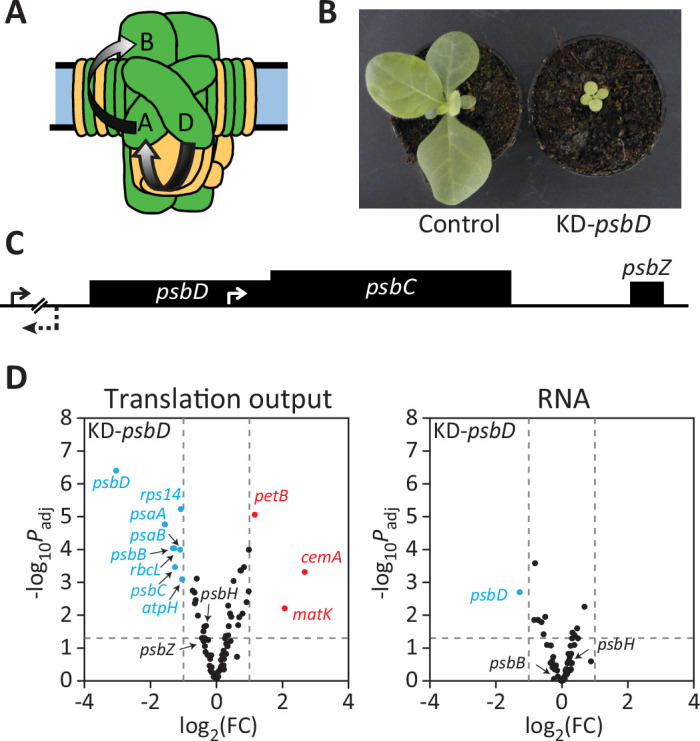
Translation output and RNA accumulation analyses of the KD-*psbD* mutant reveal a potential CES interaction between *psbD* and *psbB.* (**a**) Schematic representation showing PSII embedded in the thylakoid membrane. Labeling details are described in Extended Data Fig. 1a. CES interactions identified in Chlamydomonas are emphasized by black arrows connecting the upstream triggering subunit to the CES subunit (protein subunits are labelled with their last letter). (**b**) Phenotype of the KD-*psbD* mutant in comparison to a control line (Supplementary Table 1). (**c**) Physical map of the KD-*psbD* mutant. Black boxes comprise protein-coding regions. Black and white arrows represent the upstream and internal promoters in the *psbD*-*psbC*-*psbZ* transcription unit. The dashed arrow marks the insertion site and the transcriptional direction of the *aadA* cassette that disrupts the *psbD* 5’ UTR. Genes above the line are transcribed from left to right. (**d**) Volcano plots comparing the translation output and the RNA accumulation in the KD-*psbD* mutant in comparison to the control. Other details are as in Fig. 1b. Data were obtained from three biological replicates.

**Extended Data Fig. 3:**
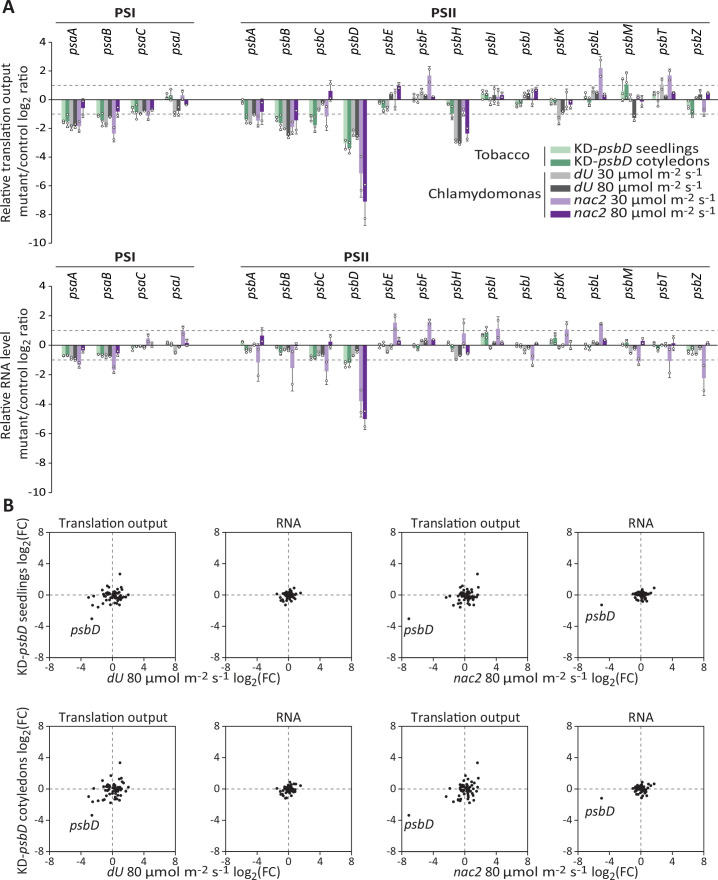
Comparison of RNA accumulation and translation output of *psbD* mutants in tobacco and Chlamydomonas. (**a**) Top and bottom diagrams: mean relative ratios of translation output and RNA accumulation in the indicated tobacco and Chlamydomonas *psbD* gene expression mutants compared to the corresponding control for chloroplast-encoded PSI and PSII genes. Error bars denote standard deviations from three biological replicates (data points of replicates are shown as dots). Note that different growth conditions and developmental stages were compared. Chlamydomonas data were taken from^12^. (**b**) Scatter plots of the relative ratios of translation output and RNA accumulation for the indicated mutants relative to the corresponding controls. Shown are all conserved chloroplast genes.

**Extended Data Fig. 4:**
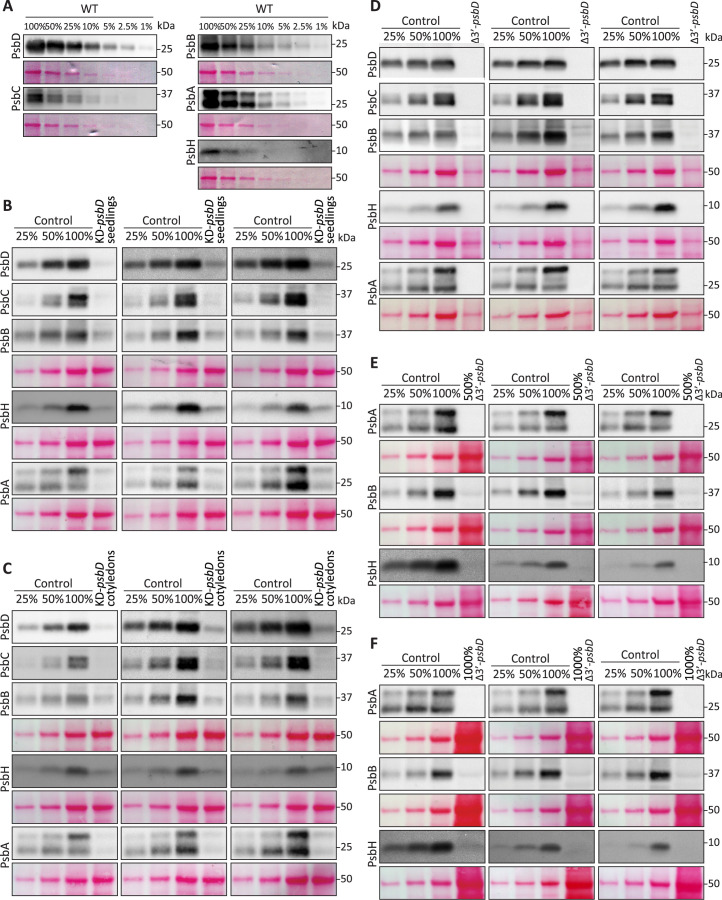
PsbB and PsbH accumulate to higher levels than other PSII subunits in KD-*psbD* and ∆3’-*psbD* mutants. Immunoblot analyses were performed to examine the protein accumulation of different PSII subunits. Protein gel blot analyses of samples of 5 µg (100%) total plant protein are shown. Equal loading was confirmed by Ponceau-S-staining (RbcL accumulation is shown). (**a**) Dilution series of wild-type protein samples to detect the sensitivity of the used antibodies. (**b** and **c**) Immunoblots show that PsbB and PsbH have a higher abundance than PsbA and PsbC in KD-*psbD* at the four-true-leave stage (seedlings) (**b**) and the cotyledon stage (**c**). (**d**) In the Δ3’-*psbD* mutant, no difference in protein accumulation was observed for PsbB and PsbH compared to other PSII subunits, due to loss of mature PSII from the *psbD*/*C* knockout mutant. (**e** and **f**) Upon overloading the mutant lane five times (**e**) or ten times (**f**), faint PsbB and PsbH bands are observed, whereas PsbA remains undetectable. Results in (**b**-**f**) are derived from three biological replicates (all three biological replicates are shown). Source data

**Extended Data Fig. 5:**
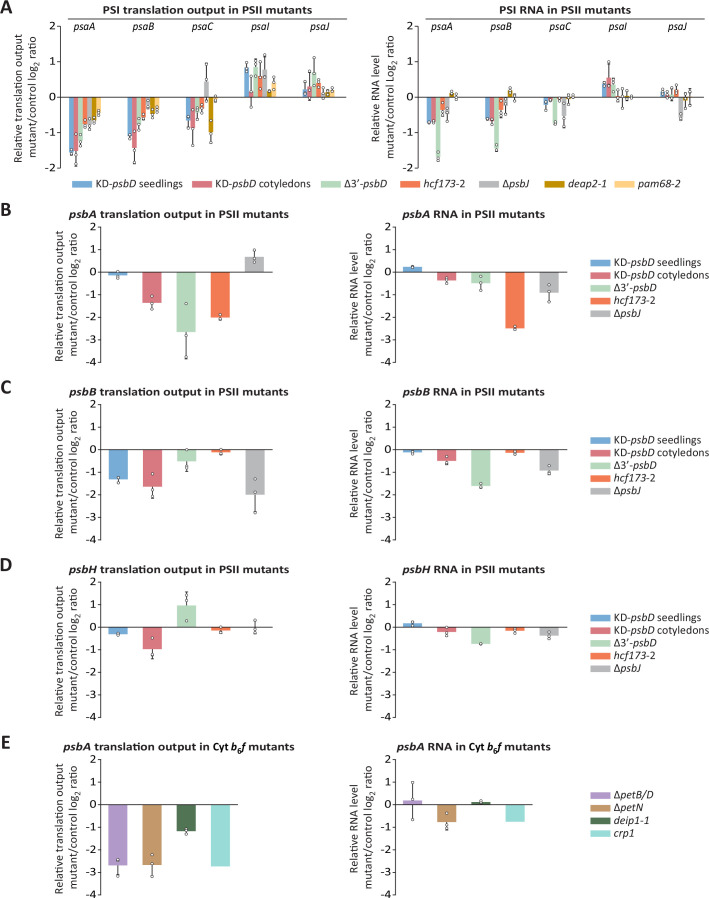
Comparison of translation output and RNA accumulation for specific chloroplast genes across different mutants illustrate shared effects. (**a**) Mean translation output (left panel) and transcript accumulation (right panel) of PSI genes relative to their corresponding controls in PSII mutants analyzed in this work and the Arabidopsis PSII assembly factor mutants *deap2-1* and *pam68-2* that have been previously analyzed and display pale-green, growth-retarded phenotypes that are caused by exon insertions of T-DNAs^33^. (**b**) *psbA*, (**c**) *psbB* and (**d**) *psbH* mean translation output (left panels) and transcript accumulation (right panels) in PSII mutants analyzed in this work. (**e**) Mean *psbA* translation output (left panel) and transcript accumulation (right panel) in Cyt*b*_6_*f* mutants analyzed in this work and the previously analyzed Arabidopsis and maize Cyt*b*_6_*f* mutants *deip1-1* and *crp1* that exhibit a Cyt*b*_6_*f* assembly defect and *petA* and *petD* translation defects that are caused by intron insertion of a T-DNA (representing a hypomorphic mutant allele) and an exon insertion of a transposon causing pale-green, growth-retarded phenotypes, respectively^25,49^. Error bars in all panels represent standard deviations of three biological replicates with data points of replicates shown as dots (note that summarized *crp1* data were extracted from the publication, hence, no standard deviation or data points are given).

**Extended Data Fig. 6:**
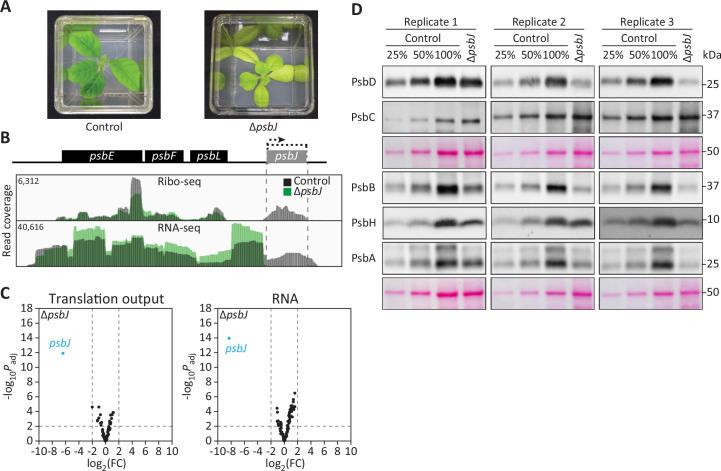
Chloroplast gene expression analysis of the ∆*psbJ* mutant. (**a**) The Δ*psbJ* mutant displays a pale green phenotype and needs to be grown heterotrophically due to its strongly diminished PSII function (Supplementary Table 1). The corresponding control line was grown under identical conditions (Supplementary Table 2). (**b**) Top panel: Physical map of the genomic region where the *aadA* cassette is inserted and fully replaces the *psbJ* reading frame (the deleted region is shaded). The dashed black arrow depicts the insertion site and the direction of transcription of the *aadA* cassette. Bottom panel: Screenshots from IGV (Integrated Genome Viewer) showing ribosome footprint and RNA coverage at the *aadA* insertion site. The y-axes represent the number of reads. The maximum y-axis values are shown in the upper left corner. (**c**) Volcano plots of the translation output (left) and RNA abundance (right) in the Δ*psbJ* mutant versus the control. Other details are as in Fig. 2b. (**d**) Immunoblot analyses of PSII subunit accumulation validating the PSII accumulation defect in Δ*psbJ*. The 100% sample represents 5 µg of total protein from tobacco. Source data

PSII comprises numerous nucleus- and plastid-encoded proteins, cofactors and lipids whose assembly is highly ordered and coordinated, a process that is largely conserved in the plant kingdom^27,28^. An early step of PSII assembly is the co-translational membrane insertion of the core subunit PsbD^27,29^. In Chlamydomonas, PsbD accumulation establishes a CES cascade in which PsbD availability regulates PsbA synthesis and accumulation of PsbA controls PsbB production^9,14^ (Fig. 1f). To address whether similar feedback regulation occurs in embryophytes, tobacco and Arabidopsis mutants with impaired PSII assembly were analysed by ribosome profiling (Figs. 1 and 2, Extended Data Figs. 2–6 and Supplementary Figs. 3–8).

First, a tobacco knockdown mutant with diminished *psbD* transcript accumulation (KD-*psbD*) was examined in comparison to a control line (Supplementary Table 1, Extended Data Fig. 2 and Supplementary Data 1). Chloroplast transcriptome analysis validated a >2-fold decrease in *psbD* mRNA accumulation (Extended Data Fig. 2d). Despite co-transcription of *psbD* with *psbC* and *psbZ*^30,31^, the downstream genes were less affected (~2-fold and <2-fold reduction, respectively; Extended Data Fig. 2c,d), probably due to the activity of an additional promoter located within the *psbD* reading frame^32^ (Extended Data Fig. 2c). Translation output of *psbD*, *psbC* and *psbZ* were >8-fold, ~2.5-fold and <2-fold reduced, respectively, arguing for an additional direct effect of the mutation on the translation of *psbD* and *psbC* (Extended Data Fig. 2d). Interestingly, the *psbD* mutant also displayed a reduced translation output of the PSII gene *psbB* (with a significant, >2-fold decrease; Extended Data Fig. 2d). *psbB* has a different genomic location and its downregulation may indicate a feedback control by PsbD levels, similar to the known CES in Chlamydomonas^12,14^. Interestingly, the translation output of the PSI core genes *psaA* and *psaB* was also significantly reduced, an effect that was previously described for other mutants with PSII defects (for example, refs. ^12,33–36^).

In Chlamydomonas, PsbA is located directly downstream of PsbD in the PSII CES cascade^9^ (Extended Data Fig. 2a). However, translation output of *psbA* was only modestly reduced in the KD-*psbD* mutant at the 4-true-leaf stage (Extended Data Fig. 2d). Since CES controls de novo PSII assembly, we reasoned that it should be particularly prominent at early developmental stages, when thylakoid biogenesis is ongoing. At later developmental stages, CES regulation of PsbA may be masked by the light-induced PsbA repair synthesis that is presumably active in both KD-*psbD* mutant and control lines^24^. To obtain a clearer picture of potential assembly-dependent feedback regulation of PsbA, tobacco seedlings of the KD-*psbD* mutant were examined at a very early developmental stage, 8 days after sowing (Supplementary Fig. 4a, referred to as cotyledon stage). Transcriptome analysis revealed a ~2-fold reduction in *psbD* and *psbC* transcript abundances (Fig. 1b), which is consistent with the previous observations at the later developmental stage (Extended Data Fig. 2d). Similarly, translation output of *psbB*, *psbD*, *psbC* and *psbZ* were more than 3-fold, 11-fold, 3-fold and 2-fold reduced, respectively (Fig. 1b,c). Despite the reduction in *psbZ* translation output, which possibly represents a direct developmental stage-dependent consequence of the mutation in the upstream *psbD*/*C* transcription unit, this reflects the results observed at the later developmental stage (Extended Data Fig. 2d), thus validating the potential feedback regulation of PsbB. Remarkably, in addition, a >2-fold (although not statistically significant) decrease in the translation output of *psbA* and *psbH* was observed in KD-*psbD* plants at the cotyledon stage (Fig. 1b,c). Reduced PsbA synthesis in KD-*psbD* plants at the cotyledon stage was validated by in vivo protein isotope labelling (Fig. 1d,e and Supplementary Fig. 6).

The KD-*psbD* mutant has defects in *psbD* and *psbC* expression (Fig. 1b and Extended Data Fig. 2d). Hence, we cannot unambiguously distinguish which of the subunits causes the translational downregulation of *psbA* and *psbB*. However, when comparing our tobacco KD-*psbD* mutant data to recently published ribosome profiling data of *psbD* knockdown mutants in Chlamydomonas^12^ (which do not have a *psbC* expression defect), a wide conservation of translational regulation in PSII and PSI core subunits was observed (Fig. 1c and Extended Data Fig. 3), supporting the idea that the *psbD* defect accounts for the *psbA* and *psbB* translational regulation. The similarities between Chlamydomonas and tobacco *psbD* mutants include the translational downregulation of the PSII and PSI transcripts of *psbA*, *psbB*, *psbH* and *psaA*, *psaB*, *psaC*, respectively (Fig. 1c and Extended Data Fig. 3). Consequently, we reasoned that similar feedback regulation occurs in the PSII assembly of Chlamydomonas and tobacco including the potential CES subunits PsbA, PsbB and PsbH, whose synthesis may be controlled by PsbD (although at this point, we could not fully exclude the possibility that diminished PsbC synthesis or accumulation is contributing to the observed effects). However, these results did not enable unambiguous conclusions about a potential hierarchy in this PSII CES regulation.

The similarities of assembly-dependent regulation in PSII subunit synthesis between Chlamydomonas and embryophytes may point to a conserved CES cascade in plants, in which PsbD availability controls PsbA synthesis and PsbA accumulation controls PsbB synthesis (Fig. 1f). However, previous analyses of the *hcf173* and *hcf244* mutants that exhibit substantial *psbA* expression defects did not reveal major effects on *psbB* expression^36–39^. To validate these findings in the light of our results, an Arabidopsis *hcf173* mutant was analysed (Supplementary Table 1). HCF173 stabilizes the *psbA* transcript and regulates its translation^37,38^. In line with a recent model of HCF173 function^24^, our transcriptome and ribosome profiling data of the *hcf173*-2 mutant suggest that, in the low-light growth conditions and at the early developmental stage analysed here, HCF173 has a major function in *psbA* transcript stabilization and, if at all, only a minor function in stimulating PsbA protein synthesis, given that no additional effect was observed at the level of translation output compared to the transcript accumulation defect (Fig. 1g,h and Supplementary Fig. 5a). However, despite the substantial reduction in *psbA* expression, *hcf173*-2 showed only marginal, non-significant effects on the translation output of *psbB*, *psbH* or other chloroplast PSII-coding genes (Fig. 1g, Supplementary Fig. 5b and Supplementary Data 1), which is in line with the previously published ribosome profiling analysis of an *hcf173* mutant^37^ and suggests that, in embryophytes, *psbB* and *psbH* translation are not controlled by PsbA.

A hypothetical alternative scenario for the PsbD-dependent feedback regulation in embryophytes could be that reduced PsbD synthesis directly triggers the translational downregulation of both *psbA* and *psbB* (potentially also *psbH*) (Fig. 1f). To examine this hypothesis further and elucidate a potential role of PsbC in PSII feedback regulation, a *psbD* knockout mutant with a deletion of the C and N termini of *psbD*- and *psbC*-coding regions, respectively, was analysed (referred to as Δ3’-*psbD*; Fig. 2a). Due to the complete knockout of PSII and photosynthesis in this mutant, the low amount of material from heterotrophic plant growth in sterile culture required next-generation sequencing (NGS)-based transcriptome and ribosome profiling. In Δ3’-*psbD*, *psbC* transcript accumulation and translation output were 8-fold and 130-fold reduced, respectively. The strong effect on *psbC* expression is expected due to the deletion of an upstream promoter and the *psbC* start codon (Fig. 2a). By contrast, ribosome footprints were gained from the 5’ end of the *psbD* reading frame (upstream of the deletion), suggesting the synthesis of a truncated PsbD protein. Indeed, a lowly abundant protein that is slightly smaller than mature PsbD was immunologically detected specifically in the Δ3’-*psbD* mutant with a PsbD-specific antibody which was raised against a peptide upstream of the deletion (high turnover of this potential truncated and non-functional PsbD protein may cause its low-level accumulation). However, our data do not enable the unambiguous assignment of the respective signal to a truncated version of PsbD (Supplementary Fig. 7). Interestingly, the Δ3’-*psbD* mutant exhibited a significant >6-fold decrease in the translation output of *psbA* (Fig. 2b,c), while the ribosome distribution of the residual ribosomes within the *psbA* reading frame was unaltered, suggesting a *psbA* translation initiation defect (Fig. 2d). In contrast to the knockdown of the full-length PsbD in the KD-*psbD* mutant, the C-terminally truncated synthesis of PsbD in Δ3’-*psbD* (and the simultaneous absence of PsbC) did not significantly affect the ribosome coverage of *psbB* and *psbH*. Together, this (1) provides additional evidence against a role of PsbA accumulation in the control of PsbB or PsbH synthesis (validating the *hcf173* results and arguing against a regulatory connection between PsbA and PsbB or PsbH; Fig. 1g), (2) may suggest that synthesis of the N terminus of PsbD is sufficient to trigger *psbB* translation (that is, the reduced expression of the PsbD N terminus in KD-*psbD* may suppress *psbB*, and potentially *psbH*, translation via a negative feedback regulation), (3) could indicate that the PsbD C terminus controls *psbA* translation (through a similar feedback loop; note that alternative interpretations of (2) and (3) are discussed below in ‘Discussion’) and (4) largely excludes a role of PsbC in the control of *psbB* or *psbH* translation in land plants (a regulatory connection that recent data may indicate in Chlamydomonas^40^) because both KD-*psbD* and Δ3’-*psbD* show strongly diminished PsbC synthesis but only the milder *psbC* mutant KD-*psbD* displays a significant decrease in *psbB* and *psbH* translation, whereas the stronger *psbC* mutant Δ3’-*psbD* does not (Figs. 1c and 2c). We cannot fully exclude the possibility that the impaired *psbC* expression found in both Δ3’-*psbD* and KD-*psbD* mutants causes reduced *psbA* translation. However, we consider this possibility to be unlikely because of (1) the known conserved assembly pathway of PSII in which PsbD and PsbA are located considerably upstream of PsbC^27^ and (2) the extensive similarities observed between *psbD* expression-dependent translational regulation in Chlamydomonas (detected in *psbD* mutants that lack *psbC* defects) with the effects observed in the herein analysed tobacco *psbD* mutants (Fig. 1c and Extended Data Fig. 3; see also ‘Discussion’). In sum, our data indicate that, in embryophytes, synthesis (or accumulation) of N- and C-terminal regions of PsbD controls the translation of *psbB* and *psbA* (Fig. 2e), respectively, as discussed in detail below (see ‘Discussion’).

We noticed that all of the PSII mutants analysed so far showed impaired *psbA* translation. To exclude the possibility that any defect in PSII function causes a reduction in *psbA* translation, a Δ*psbJ* mutant (with strongly impaired but not completely knocked-out PSII function) was examined by Ribo-seq (Extended Data Fig. 6 and Supplementary Table 1). This analysis clearly demonstrates that, despite the strongly compromised PSII function in Δ*psbJ*, *psbA* translation was not affected (Extended Data Figs. 6c and Fig. 5b), a finding that is in line with previous ribosome profiling and pulse labelling analyses of the PSII mutants *hcf107* and *lpe1* that exhibited only negligible reductions in *psbA* translation activity even though PSII accumulation was substantially impaired^23,37,39^. Together, this (1) largely excludes the possibility that any PSII defect causes reduced *psbA* translation and therefore (2) strengthens the arguments for a specific PsbD-dependent feedback regulation of *psbA* translation in embryophytes.

In Chlamydomonas, it was suggested that, in the absence of complex assembly, CES subunits can accumulate to very low levels that suffice to trigger feedback (auto-)regulation^9^. Hence, we investigated accumulation of different PSII subunits in *psbD* mutants immunologically. In the KD-*psbD* mutant, the abundances of all tested PSII subunits were reduced to negligible but detectable levels (Extended Data Fig. 4). Interestingly, PsbB and PsbH seem to be less affected in comparison to other PSII subunits (Extended Data Fig. 4). Remarkably, PsbB and PsbH were still detectable in the PSII knockout mutant Δ3’-*psbD*, suggesting that these subunits can accumulate to low levels independently of other PSII core subunits (such as PsbD, PsbC or PsbA). This may be explainable by the order of assembly events in PSII, in which PsbH and PsbB preassemble before incorporation into the reaction centre (RC) complex to convert RC into assembly intermediate RC47 (ref. ^27^). To detect PsbH-containing PSII assembly intermediates and determine their accumulation in *psbD* mutants, a blue-native–PAGE (BN–PAGE) experiment was performed on thylakoid membrane protein complexes from control plants, KD-*psbD* and Δ3’-*psbD* mutants (Supplementary Fig. 8). This analysis verified the virtual absence and strong reduction of PSII monomer, PSII dimer and PSII-containing supercomplexes in Δ3’-*psbD* and KD-*psbD*, respectively. In addition, immunoblot analysis revealed that PsbB and PsbH accumulate at low levels independently of other PSII core subunits in a smaller subcomplex, presumably RC47, a PSII assembly intermediate (Supplementary Fig. 8).

In sum, our results point to a branched CES in PSII of embryophytes that links PsbD synthesis or accumulation directly to *psbA* and *psbB* translation in PSII assembly by a translational feedback mechanism that shows similarity to but also partially differs from that described in Chlamydomonas (Figs. 1f and 2e). Previous findings suggested that PsbH is involved in CES and may control *psbB* expression^12,41^. However, based on our data, it remains elusive how and where translational control of *psbH* is integrated in the PSII CES since *psbH* translation output does not consistently follow the changes which we observed for any gene involved in PSII regulatory circuits (that is, *psbD*, *psbA* or *psbB*) of either land plants or Chlamydomonas (Extended Data Figs. 3a and 5b–d). In addition to these findings, our data validate previous evidence for a translational feedback control that adjusts PSII and PSI stoichiometries (Extended Data Figs. 3a and 5a; see also ‘Discussion’).

### Translational feedback regulation in Cyt *b*_6_*f*

**Fig. 3:**
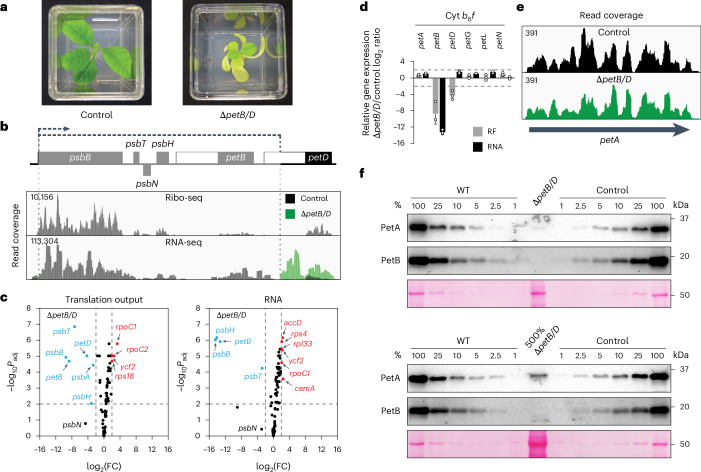
*petA* translation is not dependent on PetB or PetD accumulation. **a**, Pale green phenotype of the ∆*petB/D* mutant compared to the control line (Supplementary Table 1). **b**, Top: physical map of the relevant region in the chloroplast genome of the mutant. Genes above and below the line are transcribed from left to right and vice versa, respectively. The *aadA* cassette inserted deletes *psbN* and multiple genes in the *psbB* transcription unit including *psbB*, *psbT*, *psbH*, *petB* and the 5’ end of the *petD* reading frame (the deleted region is shaded). The dashed black arrow depicts the insertion site and transcription direction of the *aadA* cassette. Bottom: IGV screen captures of the RNA-seq and Ribo-seq analyses at the insertion site. The labelling is described in Fig. 2a. **c**, Volcano plot comparison of the translation output (Ribo-seq) and transcript abundance (RNA-seq) between mutant and control for all chloroplast reading frames. Data were collected from three biological replicates. Labelling as in Fig. 2b. **d**, Mean ± s.d. relative translation output (RF) and RNA accumulation of Cyt *b*_6_*f* genes in the Δ*petB/D* mutant in comparison to the control. *n* = 3 biological replicates shown as dots. **e**, IGV screen captures of the ribosome footprint distribution along the *petA* reading frame in the Δ*petB/D* mutant and the control. Labelling as in Fig. 2d. **f**, Immunoblot analysis to assess the accumulation of PetA and PetB in the Δ*petB/D* mutant, the wild type and the control. Top: samples of 5 µg total protein (100%) were separated by SDS–PAGE. Bottom: a 5-fold overloading of protein from the Δ*petB/D* mutant revealed a clear signal for PetA which accumulates to ~2–3% of the wild-type and control levels in the Δ*petB/D* mutant. The Ponceau-S-stained blot confirms the loading and illustrates the abundance of the large subunit of Rubisco. A biological replicate is shown in Supplementary Fig. 9. Source data

**Extended Data Fig. 7:**
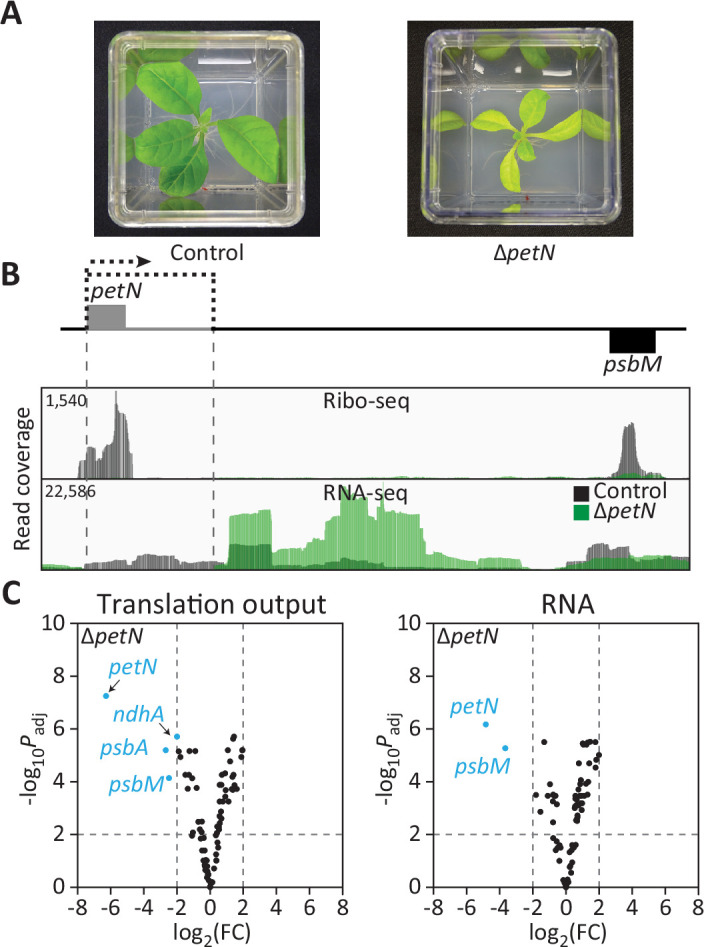
Ribo-Seq and RNA-Seq data of the ∆*petN* mutant. (**a**) Phenotypes of the heterotrophically grown Δ*petN* mutant and the control line (Supplementary Table 1). (**b**) Top panel: Physical map of the Δ*petN* mutant (for details, see Extended Data Fig. 6b). Bottom panel: Screenshots from IGV showing the ribosome footprint and RNA coverage at the *aadA* insertion site in the mutant and the control. The insertion of the *aadA* cassette causes transcriptional readthrough and overexpression of the downstream region (generating *psbM* antisense RNA and reducing *psbM* expression), an effect that has been previously described for other transplastomic lines^54^. Other details are described in Fig. 2b. (**c**) Volcano plots depicting the translation output and RNA accumulation in Δ*petN* in comparison to the control. Other details are as in Fig. 2b. Data represent three biological replicates.

Under diverse physiological growth conditions, the Cyt *b*_6_*f* complex is considered to be rate limiting for photosynthetic electron flow and depicts a homodimer, with each monomer consisting of eight protein subunits^42–46^. Little is known about the assembly of Cyt *b*_6_*f*, which is commonly assumed to occur by a pathway similar to that of mitochondrial cytochrome *bc*_1_ complexes^47–49^. Accordingly, assembly would initiate with membrane insertion of PetB (cytochrome *b*_6_) and PetD (subunit IV), followed by PetA (cytochrome *f*) and PetC (Rieske iron–sulfur protein)^44,49^. In Chlamydomonas, PetA is a CES subunit that autoregulates its synthesis depending on the availability of PetD or PetB^15,22,50,51^. To address whether a similar feedback mechanism controls Cyt *b*_6_*f* assembly in embryophytes as previously suggested^21^, a *petB*/*D*-*psbB*/*H*/*N*/*T* deletion mutant (hereafter called Δ*petB*/*D*) was examined by NGS-based ribosome and transcriptome profiling (Fig. 3 and Supplementary Table 1). As expected, the transcript accumulation and translation output of the deleted genes were decreased to background levels (Fig. 3b,c). In addition, we observed significantly increased transcript accumulation and translation output for several genes involved in chloroplast gene expression and a significant 7-fold decrease in *psbA* translation, the latter being a phenomenon that seems to be caused by any strong reduction of the Cyt *b*_6_*f* complex based on our results from another Cyt *b*_6_*f* knockout mutant and previous observations^25,49,52^ (Extended Data Figs. 5e and 7). Despite the complete absence of PetB and PetD synthesis in Δ*petB*/*D* mutant plants, ribosome coverage and distribution across the *petA* reading frame were unaltered, demonstrating that, in embryophytes, *petA* translation initiation and elongation do not require the accumulation of PetB or PetD, and occur independently of the assembly status of Cyt *b*_6_*f* (Fig. 3d,e). Previously published polysome analyses indicated a mild translational downregulation of *petA* upon deletion of *petB* or *petD* expression in tobacco^21^. However, these analyses may have suffered from technical challenges in polysome approaches in which the translational activities of *psaI*, *ycf4*, *cemA* and *petA* that are all mainly encoded on the same tetracistronic transcript^53^ are impossible to disentangle, a problem that has been solved by ribosome profiling (Fig. 3c,d).

In Chlamydomonas, PetA accumulates in the absence of PetB or PetD to ~10% of the wild-type level, which was attributed to high PetA stability (that is, low protein turnover) that may be needed to trigger auto-inhibitory feedback regulation^15^. However, in the Δ*petB*/*D* tobacco mutant, PetA accumulated only to ~2–3% of the protein level in the control and the wild type (Fig. 3f and Supplementary Fig. 9), which is in line with previous analyses of tobacco PetB and PetD knockout mutants and led to the suggestion that in land plants, different from Chlamydomonas, PetA has a very high turnover in the absence of either PetB or PetD^21^. To determine and compare PetA and PetB turnover, immunochase experiments were carried out in wild-type, control and *petA* overexpression lines (Supplementary Fig. 10; note that due to the very low abundance of PetA in Δ*petB*/*D*, we were unable to analyse PetA turnover in the mutant background). These experiments showed a similar turnover rate of PetA and PetB in all analysed genotypes (Supplementary Fig. 10). However, the unaltered translation output of *petA* in Δ*petB*/*D* (Fig. 3d) and the simultaneous strongly reduced PetA protein accumulation (Fig. 3f) suggest a very high PetA protein turnover in the absence of PetB or PetD in tobacco. This apparently strong difference in PetA turnover in the absence of PetB and PetD between land plants (high turnover) and Chlamydomonas (lower turnover) may potentially be due to different sequences in co-translational versus post-translational Cyt *b*_6_*f* complex assembly steps and the accompanying integration of haems in the respective apoproteins, the order of which awaits unravelling in both embryophytes and green algae.

**Fig. 4:**
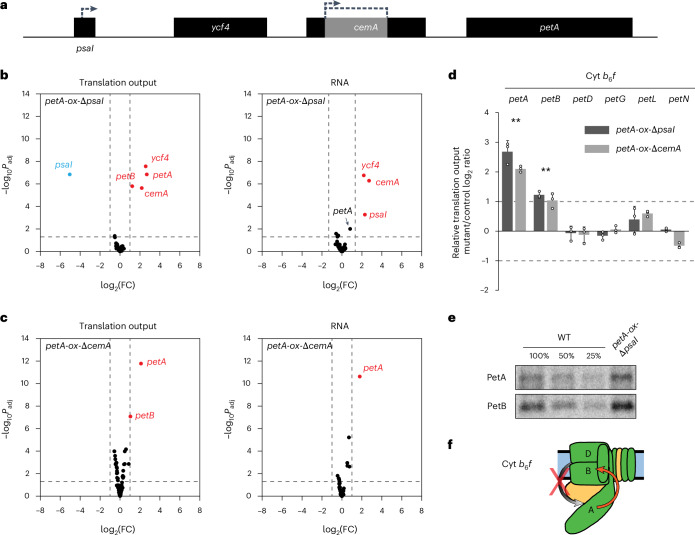
Induced expression of *petA* triggers translation of *petB* and thereby reveals translational feedback regulation in Cyt *b*_6_*f.* **a**, Physical map of the *petA*-ox-Δ*psaI* and *petA*-ox-Δ*cemA* mutants, created by inserting an *aadA* cassette in sense direction to disrupt the *psaI* and *cemA* reading frames, respectively. The resulting transcriptional readthrough causes *petA* overexpression. Dashed arrows represent the insertion sites and transcription direction of the *aadA* cassette. **b**,**c**, Volcano plot comparisons of ribosome footprint abundances and RNA accumulation for all chloroplast reading frames in the mutant relative to the control, *petA*-ox-Δ*psaI* (**b**) and *petA*-ox-Δ*cemA* (**c**) (Supplementary Table 1). Other details are described in Fig. 1b. **d**, Mean ± s.d. relative translation output of all plastid-encoded Cyt *b*_6_*f* genes in *petA*-ox-Δ*psaI* and *petA*-ox-Δ*cemA* mutants compared to control plants. Horizontal dashed lines represent the 2-fold change cut-off. Asterisks mark the significance of the change in translation output for the respective reading frames between both mutants and the control (*P*_adj_ ≤ 0.01; values shown in Supplementary Data 1). *n* = 3 biological replicates shown as dots. **e**, Pulse labelling coupled with immunoprecipitation analysis of PetA and PetB protein synthesis reveals the co-enhanced de novo synthesis of PetA and PetB in *petA*-ox-Δ*psaI*. A biological replicate is shown in Supplementary Fig. 11. **f**, Schematic representation of the structure of the Cyt *b*_6_*f* complex. The black arrow connects the assembly partner to the CES subunit (emphasized by the arrowhead), as reported in Chlamydomonas and tested in this work. The red cross reflects the absence of a Chlamydomonas-like feedback regulation from PetD and PetB to PetA (proteins labelled D, B and A, respectively), while the red arrow illustrates the potential positive feedback regulation from PetA to PetB identified in embryophytes. Source data

To further elucidate potential regulatory interactions within the Cyt *b*_6_*f* complex, the tobacco *petA* overexpression line Δ*psaI* was examined (hereafter called *petA*-ox-Δ*psaI*; Supplementary Table 1). In this line, *petA* is overexpressed by transcriptional readthrough originating from the *aadA* resistance marker inserted into the *psaI* reading frame that is located upstream of *petA*^53^ (Fig. 4a). *psaI* encodes a small non-essential PSI subunit and is part of the tetracistronic transcription unit that includes the downstream genes *ycf4* (also known as *pafII*, encoding a non-essential PSI assembly factor), *cemA* (also known as *ycf10*, encoding a non-essential chloroplast envelope membrane protein) and *petA* (Fig. 4a). As expected, transcriptome profiling revealed significantly increased transcript accumulation downstream of the *aadA* insertion site (Fig. 4b), including the 3’ region of *psaI* (5-fold), *ycf4* (5-fold), *cemA* (7-fold) and *petA* (2-fold). This observation is in line with previous notions of transcriptional readthrough originating from the *aadA* resistance marker^53,54^. The elevated transcript levels were accompanied by similar or even stronger significantly increased translation output of *petA*, *ycf4* and *cemA* (~6-fold, 5-fold and 4-fold, respectively) (Fig. 4b). This finding suggests that the elevated transcript levels of *petA* in *petA*-ox-Δ*psaI* did not deplete the RNA-binding proteins CRP1 or BSF, *trans* factors that promote *petA* translation^25,52,55,56^, to an extent that would affect *petA* translation (it should also be noted that the known additional CRP1 and BSF target mRNAs of *petD*, *ndhD* and *psaC* are not significantly or substantially affected at RNA or translational levels). Furthermore, similar overexpression levels of *petA* transcript and translation output strongly indicate that *petA* is not tightly translationally autoregulated as described in Chlamydomonas^22,51,57^. In both cases, limiting translational *trans* factor or autoregulation, *petA* transcript overaccumulation would cause translational downregulation that should result in unaltered (or limited effects on) *petA* translation output (that is, decreased translation efficiency of an increased transcript level), which we did not observe (Fig. 4b,c and Supplementary Fig. 3). These results, together with the absence of any translational downregulation of *petA* in the Δ*petB*/D mutant, the strongly diminished PetA protein accumulation in the Δ*petB*/D mutant, and the apparently different PetA protein turnover in Chlamydomonas and tobacco *petB*/*D* mutants, strongly suggest that *petA* is not feedback regulated in embryophytes as it is in Chlamydomonas.

**Extended Data Fig. 8:**
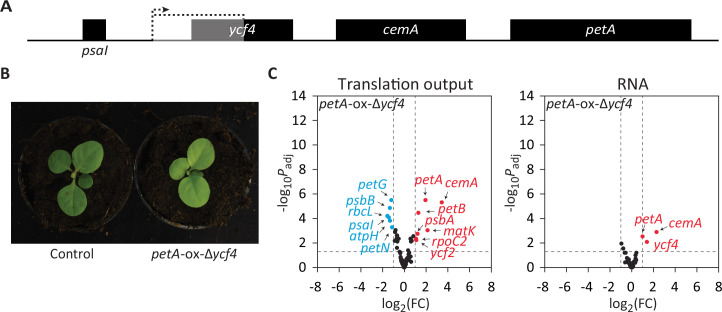
Co-enhanced translation of *petA* and *petB* in the *petA*-ox-Δ*ycf4* mutant. (**a**) Physical map of the *petA*-ox-Δ*ycf4* mutant created by replacing part of the *ycf4* reading frame by the *aadA* cassette (transcribed in sense direction; Supplementary Table 1). Labeling details are described in Fig. 4a. (**b**) Pale green phenotype of the *petA*-ox-Δ*ycf4* mutant in comparison to the control (Supplementary Table 1). (**c**) Volcano plot comparisons of the translation output and RNA accumulation for chloroplast reading frames in *petA*-ox-Δ*ycf4* relative to the control. Data were obtained from three biological replicates. The average translation output and transcript abundance per reading frame was calculated (see Material and Methods). Fold change ratios of mutant compared to control values were log_2_-transformed and plotted against the adjusted negative common logarithm of the *P* values (FDR test). Other details are as described in Fig. 1b.

Interestingly, *petA* overexpression in *petA*-ox-Δ*psaI* was accompanied by a significantly >2-fold increased translation output of *petB*, while *petB* transcript levels were not substantially altered (Fig. 4b). To rule out that this effect is related to the knockout of *psaI* (or any effect on PSI), two independent *petA* overexpression lines, Δ*cemA* and Δ*ycf4*, hereafter named *petA*-ox-Δ*cemA* and *petA*-ox-Δ*ycf4*, were analysed (Fig. 4c, Extended Data Fig. 8 and Supplementary Table 1). Both *petA*-ox-Δ*cemA* and *petA*-ox-Δ*ycf4* showed a similar transcriptional readthrough effect originating from *aadA* and causing *petA* transcript overaccumulation (Fig. 4a,c and Extended Data Fig. 8a,c). Furthermore, in both *petA*-ox-Δ*cemA* and *petA*-ox-Δ*ycf4*, the *petA* transcript overaccumulation induced increased *petA* translation output, which, in turn, was accompanied by elevated *petB* translation (Fig. 4c and Extended Data Fig. 8c). These findings (1) further substantiate the conclusion that PetA cannot be tightly translationally feedback-regulated in embryophytes, (2) validate our finding that overexpression of *petA* significantly increases the translational activity of *petB* (Fig. 4d) and thus (3) point to a regulatory connection between PetA synthesis (or protein accumulation) and *petB* translation in embryophytes. In addition, the translation output of numerous other genes was altered specifically in *petA*-ox-Δ*ycf4* (Extended Data Fig. 8c). However, these alterations cannot be related to *petA* overexpression and are likely to be a consequence of the *ycf4* knockout (and/or the strong photosynthetic deficiency in *ycf4* mutants)^58^, given that none of these changes was observed in either *petA*-ox-Δ*psaI* or *petA*-ox-Δ*cemA*. In addition, the co-occurrence of enhanced PetA and PetB synthesis was validated by protein isotope pulse-labelling experiments (Fig. 4e and Supplementary Fig. 11). If PetA synthesis or accumulation controls *petB* translation, a *petA* knockout may diminish *petB* translation. Nevertheless, previously published polysome analyses of a tobacco *petA* knockout mutant did not indicate detectable changes in *petB* translation^21^. However, conclusions on *petB* translation activity based on polysome analyses are very challenging because in land plants, *petB* is located in a large polycistronic transcription unit (Fig. 3b) whose transcripts are processed into a multitude of *petB*-containing isoforms, each of which is used as translation template^59,60^. Unfortunately, neither seeds nor tissue do exist anymore from the *petA* knockout line^21^. Hence, future attempts to examine *petB* translation activity in *petA* knockout lines in high resolution by ribosome profiling will require the generation of new transplastomic lines.

In sum, our findings strongly suggest that in embryophytes, PetA is not feedback regulated as it has been described in Chlamydomonas. Instead, our data indicate that, in tobacco, PetA synthesis and/or protein accumulation determine *petB* translation activity.

### No evidence for substantial feedback regulation in PSI

**Fig. 5:**
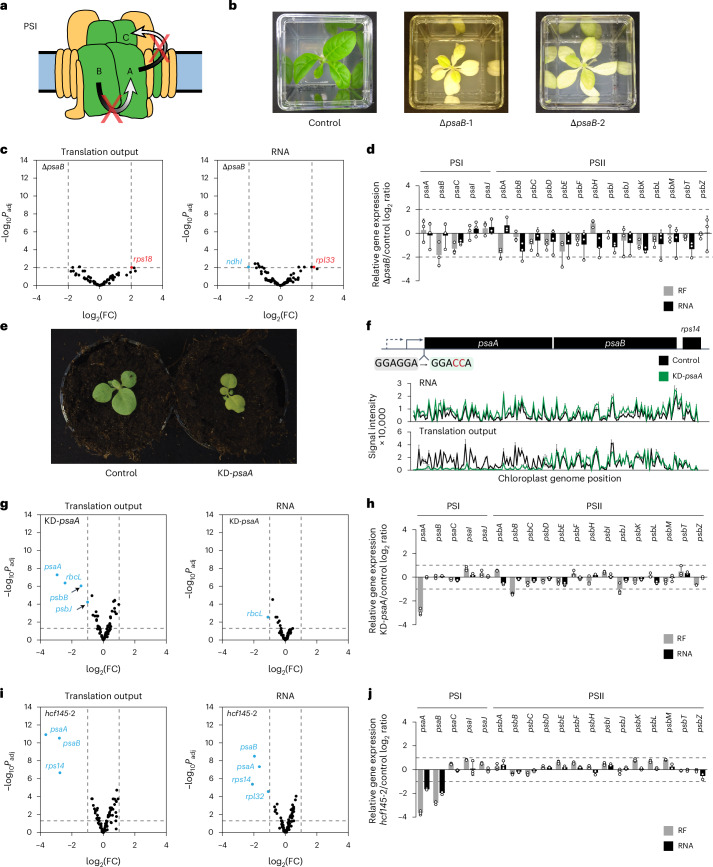
Impaired expression of *psaB* or *psaA* does not trigger CES-like regulation in embryophyte PSI genes. **a**, Schematic representation of the PSI complex with CES interactions identified in Chlamydomonas represented by black arrows (A, B, and C represent the PsaA, PsaB and PsaC proteins, respectively). The data obtained in this study do not provide evidence for a similar CES in tobacco (red crosses). **b**, The Δ*psaB*-1 and Δ*psaB*-2 mutant alleles generated in this work display very pale green phenotypes in comparison with the respective control (Supplementary Table 1). **c**, Volcano plots of the translation output and RNA accumulation of summarized results from Δ*psaB*-1 and Δ*psaB*-2 versus control (analyses of the single mutant alleles are shown in Extended Data Fig. 9). Due to similar effects on chloroplast gene expression in the Δ*psaB*-1 and Δ*psaB*-2 mutant alleles (shown in Extended Data Fig. 9), two biological replicates of Δ*psaB*-1 and one biological replicate of Δ*psaB*-2 were summarized. Other details as in Fig. 2b. **d**, Summarized mean ± s.d. relative translation output (RF) and RNA accumulation of PSI and PSII-related genes in the Δ*psaB* mutant alleles compared to the control line. *n* = 3 replicates (same as in **c**) shown as dots. **e**, The KD-*psaA* mutant harbouring a mutated *psaA* Shine–Dalgarno (SD) sequence displayed a pale green phenotype in comparison with the wild-type-like appearance of the control line (Supplementary Table 1). **f**, Top: physical map of the mutant showing the location of the selectable marker (*aadA* cassette) inserted upstream of the start codon of *psaA*. The SD was mutated from GGAGGA to GGACCA, causing strongly reduced translation initiation of *psaA* (wild-type and mutant SD sequences are shaded in grey and green, respectively). Bottom: the translation output of the downstream *psaB* and *rps14* reading frames is not affected, demonstrating uncoupled translation of the co-transcribed reading frames. **g**,**i**, Volcano plots comparing the translation output and RNA accumulation of all chloroplast reading frames in KD-*psaA* and the control (**g**), as well as the *hcf145*-2 mutant and its corresponding wild-type control (**i**). The results were obtained from three biological replicates for each of the genotypes. Other details as in Fig. 1b. **h**,**j**, Mean ± s.d. relative translation output and RNA accumulation of PSI and PSII genes in the KD-*psaA* (**h**) and *hcf145*-2 mutants (**j**) in comparison to their corresponding controls. *n* = 3 biological replicates shown as dots.

**Extended Data Fig. 9:**
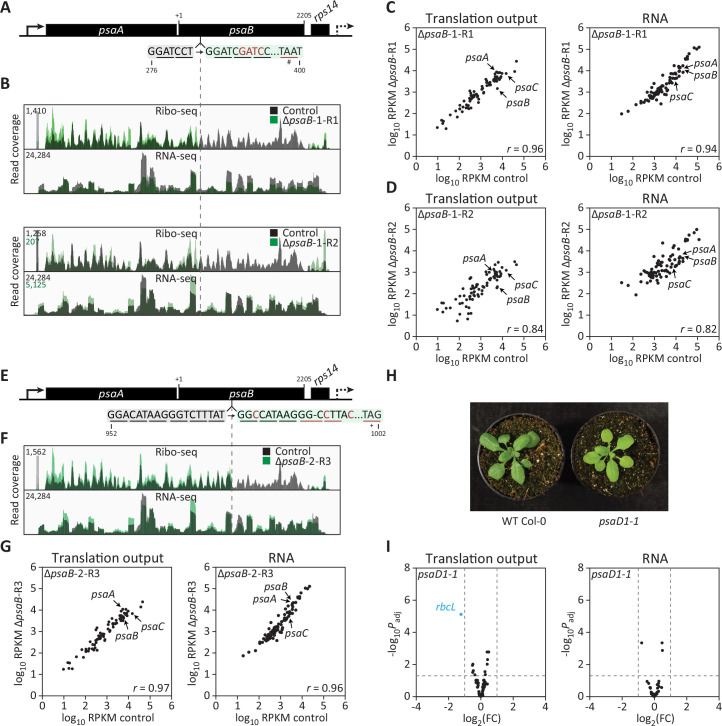
No evidence for PsaB- or PsaD-dependent CES in PSI of embryophytes. (**a**) Physical map of the Δ*psaB*-1 mutant allele, which was created by inserting the four nucleotides GATC 280 nucleotides downstream of the start codon, leading to a frame-shift and premature translation termination at an in-frame stop codon (the control and mutant DNA sequences are shown in gray and green shaded boxes below the map, respectively; the complete *psaB* sequences of the control line and mutant alleles are shown in Supplementary Table 3). The dashed arrow represents the *aadA* insertion site and indicates the transcriptional direction of the *aadA* cassette. (**b**) IGV snapshots of the mutation site that disrupts the *psaB* reading frame in Δ*psaB*-1, analyzed as replicates 1 and 2 in Fig. 5b-d (top and bottom panels, respectively). Other details are described in Fig. 2a. Evidence for the premature stop codon is provided by the missing downstream footprint coverage. Note that the obtained raw read coverage of Δ*psaB*-1 replicate 2 (Δ*psaB*-1-R2) was lower than for the control or Δ*psaB*-1 replicate 1 (Δ*psaB*-1-R1). Hence, we increased the y-axis scale to make the equal read distribution within *psaA* and *psaB* reading frames and the premature translation termination visible. All other diagrams shown in this figure and in Fig. 5 are based on normalized reads that are not influenced by differences in read coverage between samples (normalization described in the Methods). (**c**, **d**) Scatter plot comparisons of the footprint abundance and RNA accumulation for chloroplast reading frames in two biological replicates of Δ*psaB*-1 relative to the control. The averages of ribosome footprint and transcript abundance per reading frame were calculated. Other details are as described in Fig. 2b. (**e**) Physical map of the Δ*psaB*-2 mutant allele analyzed as replicate 3 in Fig. 5b-d. Deletion of a thymidine nucleotide at position 963 downstream of the start codon led to a frame-shift and premature translation termination at an in-frame stop codon at position 1002 relative to the start codon (in addition, the other shown point mutations were detected, whose consequences are likely irrelevant; labeling as in **a**). (**f**) IGV snapshot showing the read coverage at the mutation site. Evidence for the premature stop codon is provided by the absence of downstream footprint coverage. The translated 5’ *psaB* coding region in the Δ*psaB*-2 mutant allele shown in (**e**-**f**) is longer than that of the truncated *psaB* reading frame in the Δ*psaB*-1 mutant allele (shown in (**a**-**d**)). For both mutant alleles, more than half of the *psaB* reading frame remains untranslated due to the premature stop codons. (**g**) Scatter plot comparisons of the footprint abundance and RNA accumulation for chloroplast reading frames in Δ*psaB*-2 relative to the control. Other details are described in Fig. 2a. (**h**) Pale green phenotype of the *psaD1-1* T-DNA insertion mutant in comparison to wild-type Col-0 (WT). In Arabidopsis, PSAD is encoded by two paralogous and functionally redundant genes, *PSAD1* and *PSAD2*^70^. The knock out of *PSAD1* causes a 60% decrease in PSI^70^. (**i**) Comparison of the translation output and RNA accumulation of all chloroplast reading frames in *psaD1-1* represented in volcano plots. Data were obtained from three biological replicates. Other details are as in Fig. 1b.

The plastocyanin–ferredoxin oxidoreductase PSI contains 15 subunits and depicts the second largest multimeric protein complex in the thylakoid membrane^61,62^. PSI assembly initiates with co-translational thylakoid membrane insertion of PsaB and PsaA^29^, which is followed by PsaC attachment^63,64^. In Chlamydomonas, PsaB establishes a CES cascade, in which availability of PsaB controls *psaA* translation, and PsaA accumulation limits PsaC synthesis^16^. To check whether comparable feedback regulation is involved in the assembly of PSI in embryophytes, two independent transplastomic *psaB* knockout alleles Δ*psaB-1* and Δ*psaB-2* were generated by introducing frameshift-causing point mutations into the *psaB* reading frame (Fig. 5b–d, Extended Data Fig. 9a–g and Supplementary Table 3). Ribosome profiling analysis of these mutants confirmed that both mutant alleles cause premature *psaB* translation termination at positions that preclude functionality of the synthesized truncated PsaB protein (in that more than half of PsaB is missing), an assumption that is supported by the pale green phenotype and the inability of these mutants to grow autotrophically, a phenotype that resembles previously published PSI knockout mutants^65,66^ (Fig. 5b–d and Extended Data Fig. 9a–g). Because the observed mild changes in chloroplast gene expression were similar in both *psaB* mutant alleles (Extended Data Fig. 9a–g), we combined their analysis to identify potential PsaB-dependent feedback regulation. This examination showed that, apart from the premature translation termination in the *psaB* reading frame, the expression of no other chloroplast-encoded PSI gene was substantially and significantly altered at the transcript accumulation or translational levels (Fig. 5c,d, Extended Data Fig. 9a–g and Supplementary Data 1), arguing against a Chlamydomonas-like CES that would originate from PsaB and act in the assembly of embryophyte PSI. Although we noticed a mild reduction in *psaC* translation output (Fig. 5d), this effect was not validated in an independent Arabidopsis mutant with strongly diminished *psaB* expression (Fig. 5j).

To be able to detect potentially independent CES acting downstream of PsaB, a tobacco *psaA* translation knockdown mutant (KD-*psaA*) as well as the Arabidopsis *hcf145*-2 mutant that exhibits impaired *psaA*/*B*-*rps14* transcript accumulation were examined by ribosome profiling (Fig. 5e–j and Supplementary Table 1). KD-*psaA* and *hcf145*-2 mutants displayed the expected defects in *psaA* translation and *psaA*/*B*/*rps14* transcript accumulation, respectively (Fig. 5e–j). Most importantly, these defects did not induce significant >2-fold alterations in the translation output of *psaC* or any other plastid-encoded PSI gene, implying the absence of PsaA or PsaB-dependent CES. Interestingly, in *hcf145*, the *psaA* translation defect is more than four times as strong as the corresponding defect in transcript accumulation, suggesting that HCF145 promotes *psaA* translation, in addition to its known function in *psaA* transcript stabilization^67,68^. Such dual functions have been described before for other chloroplast RNA-binding proteins^69^. In KD-*psaA*, translation output of *psaB* and *rps14* were not significantly (or >2-fold) altered, despite their co-transcription with the upstream translationally strongly impaired *psaA* (Fig. 5f,h). This observation implies the uncoupled translation of *psaA* and *psaB*/*rps14*, all of which are located on the same tricistronic transcript.

Finally, a knockdown mutant of the peripheral PSI subunit PSAD (*psaD1-1*) was examined (Extended Data Fig. 9h,i and Supplementary Table 1). In Arabidopsis, the PSI subunit PSAD is encoded by the two paralogous and functionally redundant genes *PSAD1* and *PSAD2* (ref. ^70^). Although the knocked-out expression of *PSAD1* causes a 60% decrease in PSI accumulation^70^, no significant >2-fold alteration was observed in the expression of chloroplast PSI genes (Extended Data Fig. 9i). Similar observations were previously made in the knockout mutant of the peripheral non-essential PSI subunit PsaJ^54^.

Together, our results indicate that CES-like feedback regulation does not substantially contribute to the stoichiometric accumulation of PSI subunits in embryophytes.

### No indication for feedback regulation in the NDH complex

**Extended Data Fig. 10:**
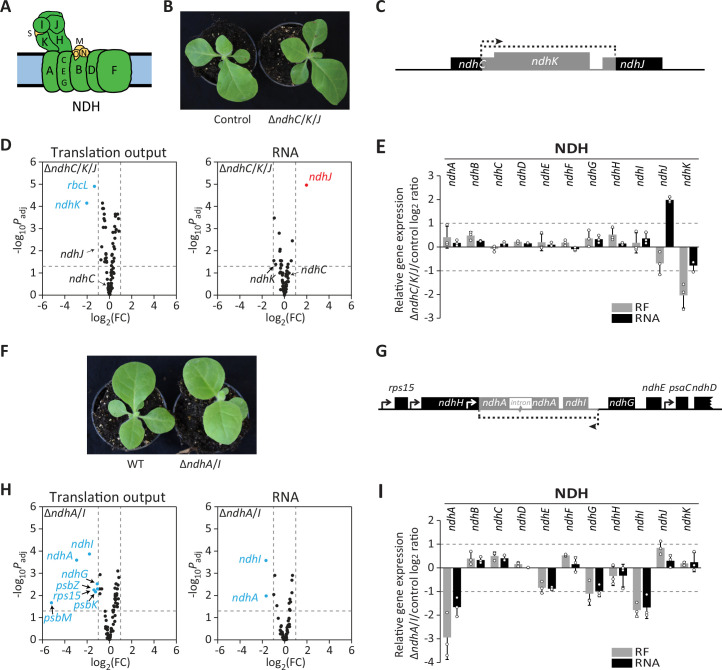
No evidence for CES in the chloroplast NDH complex. (**a**) Schematic representation of the NDH complex in the thylakoid membrane of embryophytes (protein subunits are labelled with their last letter; other details as described in Fig. 1f). The NDH complex does not exist in the chloroplast of Chlamydomonas and no CES has been described for this complex. (**b** and **F**) Wild-type-like phenotypes of the Δ*ndhC*/*K*/*J* and Δ*ndhA*/*I* mutants. (**c** and **g**) Physical map of the Δ*ndhC*/*K*/*J* (**c**) and Δ*ndhA*/*I* mutants (**g**) showing the insertion site of the *aadA* cassette and known promoters in the *ndhH*/*A*/*I*/*G*/*E*/*psaC*/*ndhD* transcription unit (details as described in Extended Data Fig. 6b). (**d** and **h**) Volcano plots comparing the translation output and the RNA accumulation in Δ*ndhC*/*K*/*J* (**d**) and Δ*ndhA*/*I* mutants (**h**) versus control and wild-type plants, respectively. Results are from three biological replicates. Other details are as in Fig. 1b. (**e** and **i**) Mean relative translation output (RF) and RNA accumulation of plastid-encoded NDH complex genes in Δ*ndhC*/*K*/*J* (**e**) and Δ*ndhA*/*I* mutants (**i**). Error bars represent standard deviations of three biological replicates (data points of replicates are shown as dots).

To examine potential CES in the NDH complex of land plants (a photosynthetic complex that is absent from the Chlamydomonas chloroplast), we analysed the tobacco Δ*ndhC*/*K*/*J* and Δ*ndhA*/*I* mutants, which simultaneously knock out three and two NDH subunits that are encoded in different genomic loci, respectively (Supplementary Results, Extended Data Fig. 10 and Supplementary Table 1). These analyses did not reveal any indication of feedback regulation in the chloroplast NDH complex.

### Potential feedback regulation in ATP synthase

**Fig. 6:**
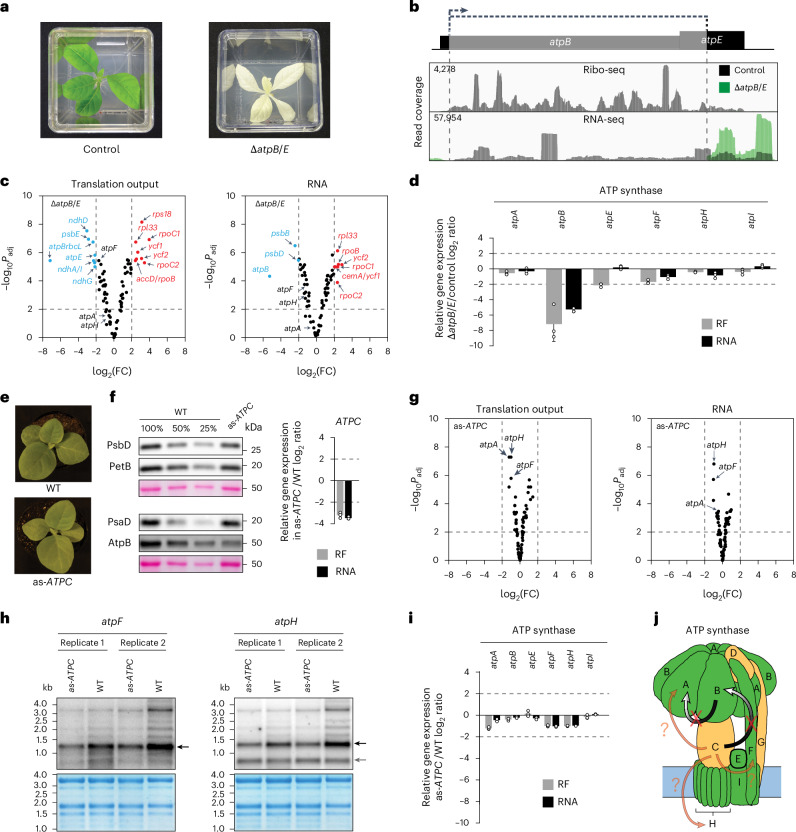
Potential feedback regulation in ATP synthase. **a**, White phenotype of the Δ*atpB*/*E* mutant in comparison to a control line (Supplementary Table 1). **b**, Top: physical map illustrating the deletion caused by the insertion of the *aadA* cassette within the *atpB* and *atpE* reading frames. Bottom: IGV screenshots of the coverage of the Ribo-seq and RNA-seq reads in the Δ*atpB*/*E* mutant and the control at the insertion site. Other details as in Fig. 2a. **c**, Volcano plots comparing the translation output and RNA accumulation in Δ*atpB*/*E* mutant versus control. Summary of three biological replicates is shown. Other details as in Fig. 2b. **d**, Mean ± s.d. relative translation output (RF) and RNA accumulation of plastid-encoded ATP synthase genes. *n* = 3 biological replicates shown as dots. **e**, as-*ATPC* mutant displaying a pale green phenotype in comparison with the wild type (WT). **f**, Left: immunoblot analyses to determine the accumulation of photosynthetic complexes in the as-*ATPC* mutant in comparison to the wild type (confirming the ATP synthase accumulation defect). The 100% sample represents 5 µg of total protein. Right: relative ratios of the *ATPC* translation output (RF) and RNA accumulation in as-*ATPC* compared to the wild type. Error bars represent standard deviations of three biological replicates shown as dots. **g**, Volcano plot comparison of the translation output and RNA accumulation in the as-*ATPC* mutant versus the wild-type control for all chloroplast reading frames. Other details as in Fig. 2b. **h**, RNA gel blot analyses with probes specific to *atpF* (left) and *atpH* (right). Methylene blue staining of rRNAs is shown as a loading control below each blot. RNA ladder sizes are labelled on the left side. The black and grey arrows indicate the dicistronic *atpH*-*atpF* and the monocistronic *atpH* transcripts, respectively. **i**, Mean ± s.d. relative translation output (RF) and RNA accumulation in the mutant compared to WT for the chloroplast-encoded ATP synthase genes. *n* = 3 biological replicates shown as dots. **j**, Scheme of the ATP synthase complex with the CES between ATPC, AtpB and AtpA reported in Chlamydomonas (ATP synthase complex subunits are labelled with their last letter). The red crosses reflect the absence of these CES interactions in the ATP synthase complex of embryophytes suggested by our experiments. Presumable regulatory interactions that did pass our threshold for significance (but not that for fold change) are shown in shaded colour with a question mark (in addition, a mild but significant *atpF* downregulation has been observed in Δ*atpB/E* but the origin of this effect could not be assigned unambiguously due to the knockout of two *atp* genes in this mutant). Source data

In chloroplasts, the thylakoid CF_0_–CF_1_ ATP synthase catalyses ATP synthesis on the basis of a proton gradient that is established by the photosynthetic light reactions. There is evidence that the chloroplast ATP synthase assembles modularly, similar to bacterial and mitochondrial ATP synthases, and some of the involved factors have been identified^71^. Different from other photosynthetic complexes, ATP synthase subunits are assembled in a non-equimolar stoichiometry (AtpA:AtpB:ATPC:ATPD:AtpE:AtpF:ATPG:AtpH:AtpI = 3:3:1:1:1:1:1:14:1)^44,71^. In embryophytes and Chlamydomonas, this stoichiometry is reflected by an accordingly adjusted expression of the respective genes with transcriptional and translational contributions, demonstrating the participation of translational regulation to stoichiometric ATP synthase assembly^11–13^. In addition, a CES cascade fine-tunes the translation of ATP synthase genes in Chlamydomonas^17^ in that ATPC is required for the translation of *atpB* which, in turn, triggers the synthesis of AtpA (Fig. 6j).

It has been previously shown in maize and tobacco that *atpB* knockdown does not affect *atpA* translation^12,25^. To rule out any mild feedback effects that may have gone undetected in the previously analysed hypomorphic mutant alleles, an *atpB*/*E* knockout mutant (Δ*atpB*/*E*) was examined by Ribo-seq and RNA-seq (Fig. 6a–d, Supplementary Data 1 and Supplementary Table 1). As expected, translation output of *atpB* and *atpE* were decreased to background levels in Δ*atpB*/*E* (Fig. 6b). In addition, expression of none of the other plastid-encoded ATP synthase subunits was significantly >4-fold affected (Fig. 6c,d). We noticed, however, that a mild but significant reduction was observed for *atpF* transcript accumulation and translation output (and could originate from *atpB* and/or *atpE* knockout). Interestingly, knockout of the ATP synthase induced substantial and significant alterations in the expression of many chloroplast genes (Fig. 6c). This molecular phenotype is in line with the visible white appearance of the Δ*atpB*/*E* mutant, a phenotypic effect that is more drastic than that of any of the tobacco PSI, PSII or Cyt *b*_6_*f* knockout plants (Figs. 3 and 5, Supplementary Fig. 4 and Extended Data Fig. 7). These drastic effects may be due to the massive alterations in energy status or proton gradient across the thylakoid membrane (and the resulting alterations in lumenal and stromal pH). In sum, and in line with previous observations^12,25^, the Δ*atpB*/*E* data do not provide evidence for feedback regulation between AtpB and AtpA as described in Chlamydomonas^10^.

The nucleus-encoded ATPC depicts the most upstream subunit in the CES cascade of the ATP synthase in Chlamydomonas^17^ (Fig. 6j). Consequently, an *ATPC* knockdown mutant (as-*ATPC*) was analysed by ribosome and transcriptome profiling (Supplementary Table 1). In line with the previous description of the mutant^72^, as-*ATPC* has a pale green phenotype that is caused by an ~11-fold decrease in *ATPC* transcript accumulation that provokes a similarly diminished translation output and a 4-fold reduction in ATP synthase accumulation (Fig. 6e,f). Interestingly, as-*ATPC* showed a significant, although <4-fold, reduction in the accumulation of dicistronic *atpH*-*atpF* transcripts (Fig. 6g–i). This defect in RNA accumulation caused a significantly (albeit only 2-fold) reduced translation output of *atpH* and *atpF* (Fig. 6g,i). Furthermore, *atpA* translation output was significantly (albeit only 2-fold) decreased in as-*ATPC*.

In sum, in embryophytes, the herein presented and previously published analyses did not reveal AtpB-dependent feedback regulation on *atpA* translation (or any substantial effect of *atpB*/*E* knockout on the expression of other chloroplast-encoded *atp* genes). Our data may, however, hint at a potential mild feedback regulation that depends on ATPC availability or synthesis and controls the transcript levels of *atpH* and *atpF* (and potentially translation of *atpA*) (Fig. 6g–j).

## Discussion

**Fig. 7:**
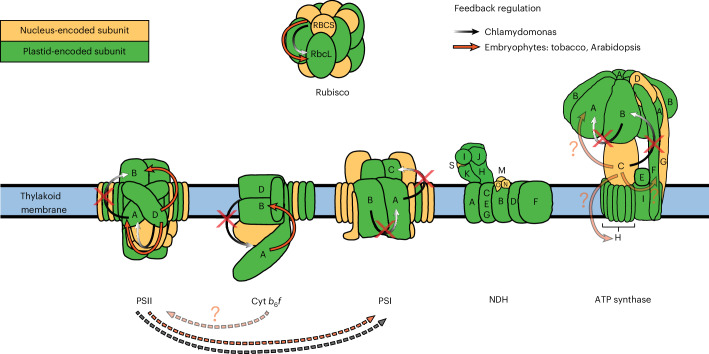
Comparative overview of translational feedback regulation in the assembly process of photosynthetic protein complexes of land plants and Chlamydomonas. Plastid-encoded subunits are represented in green and nucleus-encoded subunits are depicted in yellow (protein subunits of light reaction complexes are labelled with their last letter). Black arrows indicate CES interactions previously observed in Chlamydomonas^10^. Embryophyte CES interactions previously shown or discovered in this work are indicated with red arrows. Chlamydomonas CES that is not conserved in land plants is marked with red crosses. Presumable regulatory interactions that did not pass our thresholds for fold change (but showed significance) are shown in shaded colour with a question mark. Dashed arrows depict regulatory interactions between photosynthetic complexes (the control of PSI core subunit expression by PSII is well supported, whereas the control of *psbA* translation by Cyt *b*_6_*f* is less well supported and therefore shaded and shown with a question mark; note that other potential translational regulations across photosynthetic and chloroplast biogenesis-related protein complexes are suggested by the data presented in this work; for details see ‘Results’ and ‘Discussion’). The figure was adapted from ref. ^9^ and modified by incorporating the data obtained in this study.

Translational feedback regulation is widespread in the assembly of photosynthetic complexes in the chloroplast of Chlamydomonas but has been conclusively demonstrated in embryophytes only for Rubisco assembly^10^. Our systematic evaluation of chloroplast gene expression in embryophyte mutants with assembly defects in PSII, Cyt *b*_6_*f*, PSI, ATP synthase and the NDH complex revealed several cases of feedback regulation (Fig. 7). Similar to the Chlamydomonas CES, most of this feedback regulation was observed at the translational level (except for a potential transcript-level feedback regulation between ATP synthase subunits; Fig. 6). Therefore, our findings provide additional evidence for the general predominance of translational regulation in adjustments of chloroplast gene expression in embryophytes, which was previously described, for example, in response to changing light and temperature^13,23,69,73–76^. Similar to Chlamydomonas, all newly identified embryophyte feedback regulatory mechanisms in PSII, Cyt *b*_6_*f* and ATP synthase are milder than the primary, CES-triggering defect. For example, an 11-fold reduction in *psbD* expression induced only ~4-fold and 3-fold decreases in *psbB* and *psbA* translation, respectively (Fig. 1c). Likewise, mild effects were observed in the *psbD* knockout line and the analysed mutants in other photosynthetic complexes that induced feedback regulation, suggesting a limited dynamic range of the CES regulatory response (Figs. 2, 4 and 6). Together, these findings imply that, similar to Chlamydomonas^10^, CES in embryophytes on demand fine-tunes the stoichiometric synthesis of specific subunits during the assembly of photosynthetic complexes. In this role, the dynamic CES regulation acts in addition to the static control of expression levels for different chloroplast genes, the latter being determined by fixed *cis* elements of different strengths for transcription, transcript processing and translation, which usually already lead to translation output levels that very well reflect the stoichiometry of proteins in complexes (such as ATP synthase), although not perfectly under steady-state conditions (for example, refs. ^11–13^). Hence, the limited dynamic range of CES can be assumed to be fully sufficient to comply with assembly-accompanying differential changes in the turnover of specific photosynthetic complex subunits that may be induced, for instance, by environmental or developmental triggers. In addition to these different levels of gene expression regulation, proteolytic degradation of unassembled subunits also plays a role in the coordinated assembly of photosynthetic protein complexes. If a CES subunit is less feedback downregulated than its CES-triggering assembly partner but both proteins accumulate to similarly low levels, as observed in both embryophytes and Chlamydomonas^10^ (Figs. 1, 2 and 6 and Extended Data Figs. 3 and 4), proteolysis must account for the difference between protein synthesis rate and protein accumulation level (the same holds true for artificially upregulated genes for which the encoded proteins do not accumulate to a level suggested by the increase in translation output as observed for PetA in the *petA* overexpression lines; Fig. 4 and Supplementary Fig. 10). However, the contribution of proteolysis to stoichiometric subunit accumulation may be strongly overestimated if assessed on the basis of mutants that artificially knockout, knockdown or overexpress specific photosynthetic proteins, a situation that obviously never occurs naturally in complex assembly.

The observed regulation in embryophyte PSII and the potential feedback regulation in ATP synthase assembly depict negative feedback control mechanisms similar to most CES mechanisms described in Chlamydomonas^10^. In those cases, the reduced synthesis or accumulation of a specific photosynthetic protein (or parts thereof) causes the downregulated expression of its assembly partner(s) (Fig. 7). However, in Cyt *b*_6_*f* assembly, the increased expression of *petA* promoted the translation of *petB* (Fig. 4 and Extended Data Fig. 8), suggesting that PetA synthesis and/or accumulation limit, to some extent, the translation of *petB* and probably the accumulation of the whole Cyt *b*_6_*f* complex, which may reflect a positive feedback control (which can potentially also act as negative feedback if PetA becomes limiting, a hypothesis that can be tested in future with suitable *petA* mutants). Our observations are in line with the previous description of increased Cyt *b*_6_*f* complex accumulation in one of the *petA* overexpression lines analysed here^53^. Similar to the identified negative feedback regulation in PSII and ATP synthase, the effect of the potential positive feedback regulation in Cyt *b*_6_*f* is rather mild in that a >4-fold overexpression of *petA* led to a ~2-fold increase in *petB* translation and an even milder overaccumulation of Cyt *b*_6_*f*^53^ (Fig. 4 and Extended Data Fig. 8). This may not be surprising, considering that upon *petA* overexpression, Cyt *b*_6_*f* accumulation is certainly still limited by other factors involved in Cyt *b*_6_*f* biogenesis (for example, factors serving gene expression, assembly, co-factor binding, targeting and so on of Cyt *b*_6_*f* subunits).

In future experiments, transcriptional overexpressors of chloroplast-encoded proteins can provide suitable study objects to disprove or identify translational feedback regulation (similar to the *petA* overexpression lines herein). If for a specific transcript substantial overaccumulation is induced and this directly causes a similarly increased level of translation output/protein synthesis, this result would largely disprove translational feedback regulation. This is the case because a tight translational feedback regulation would reduce translational efficiency of an increased transcript level and thereby keep translation output constant (or at least limit its increase). Following this logic, *psbD*, *psbC*, *petG*, *petA*, *psaJ*, *rpl33*, *rps18, ycf4* and *cemA* are unlikely to be tightly translationally feedback regulated because we have shown herein or previously that their artificially induced transcript overaccumulation is accompanied by a comparable increase in translation output^54,77^ (Fig. 4 and Extended Data Fig. 8). Furthermore, overexpression approaches can disclose subunits that limit the accumulation of a complex (or the expression of its subunits) as shown for PetA herein and previously disproven for PsbD and PsbC^53,77^ (Fig. 4 and Extended Data Fig. 8). If, on the other hand, substantially elevated transcript levels do not induce increased translation output (that is, decreased translation efficiencies are observed for increased transcript levels), this could (1) indicate the depletion of transcript-specific *trans* factors needed for translation activity and/or (2) suggest a translational feedback regulation—two options that can be mutually related but need to be unravelled carefully in a case by case manner with appropriate molecular analyses.

In this work, we provide evidence for some similarities between chloroplast feedback regulation in the assembly of photosynthetic subunits in plants and the CES processes described in Chlamydomonas (for example, the PsbD-dependent translational regulation of *psbA*). However, there are also notable differences (Fig. 7). For PSII, our data indicate that PsbD controls both *psbA* and *psbB* translation. This suggests that there is no CES cascade in embryophyte plants, but rather a branched regulation controlled by PsbD synthesis and/or accumulation (Fig. 2e). The observed absence of a regulatory connection between PsbA and PsbB is also supported by previous analysis of embryophyte mutants with PsbA synthesis defects that did not exhibit co-reduced PsbB synthesis (for example, refs. ^36–39^). A possible interpretation of our data is that the N-terminal region of PsbD needs to be expressed for PsbB synthesis, whereas the C-terminal region controls PsbA synthesis (Fig. 2). This hypothesis is supported by the known interactions of the N- and C-terminal parts of PsbD with PsbB and PsbA, respectively, within assembled PSII^28,78^. Since these interactions between PSII subunits and their assembly are conserved in embryophytes and Chlamydomonas, it remains to be determined why partially different regulatory connections have evolved in green algae and embryophytes. The recently suggested assembly-dependent auto-feedback regulation of *psbA* translation during PSII repair^23,24^ seems to be sufficient to also explain the regulatory connection between the assembly partners PsbD and PsbA in PSII biogenesis of embryophytes observed in this work. However, although most of the known *trans* factors involved in PsbA repair synthesis are conserved between embryophytes and Chlamydomonas, not all molecular details of *psbA* translational regulation are fully understood, and there also seem to be differences in PSII repair between embryophytes and the green alga^79^. Alternative to the model shown in Fig. 2e, one may speculate that potentially different *psbD* expression levels and/or PsbD turnover rates between the herein analysed *psbD* mutant alleles may trigger the observed dissimilar responses in *psbA* and *psbB* translation (Figs. 1 and 2).

Similar to PSII, different regulatory connections were also found between Chlamydomonas and embryophytes for the assembly of Cyt *b*_6_*f* (Fig. 7). In Chlamydomonas, the protein factors MCA1 and TCA1 are cooperatively associated with the 5’UTR of the *petA* mRNA, and stabilize the transcript and stimulate its translation, respectively^10^. A C-terminal domain of the unassembled PetA protein has been shown to bind to MCA1 and cause its proteolytic degradation^22^. This leads to the downregulation of *petA* gene expression on transcript and translation levels, and ensures adequate synthesis levels of PetA during assembly of the Cyt *b*_*6*_*f* complex (that is, according to the availability of PetB and PetD). Although the C-terminal PetA domain is conserved in embryophytes^10^, the MCA1 and TCA1 expression factors are not found in land plants, and embryophyte *petA* translation depends on the *trans* factors CRP1 and BSF^25,52,55,56^, which do not seem to be strictly limiting for *petA* translation (as proven herein by increased *petA* translation output in transcriptional *petA* overexpression lines; Fig. 4 and Extended Data Fig. 8). Also different from Chlamydomonas, in embryophytes, *petB*/*D* knockout does not cause reduced *petA* expression (Fig. 3c–e), and PetA does only accumulate to ~2–3% of the wild type in the absence of PetB or PetD (Fig. 3f), the latter being presumably caused by a high PetA turnover rate in the absence of PetB and PetD. Together, this strongly suggests that different regulatory connections were established in embryophytes for which PetA accumulation and/or synthesis triggers *petB* translation (Fig. 7).

Our data provide some evidence that in the ATP synthase assembly of embryophytes, ATPC controls to some extent *atpA* translation and the accumulation of a specific dicistronic *atpF*/*atpH* transcript isoform, which would depict regulatory connections that differ substantially from the CES cascade described in the Chlamydomonas ATP synthase^17^ (Fig. 7). Indeed, for the regulation of chloroplast *atp* gene expression, the known involved *trans* factors also differ between the green alga and embryophytes^71^. Overall, these results may therefore suggest the independent but partially convergent evolution of somehow similar but not identical regulatory connections in the photosynthetic complex assembly of the green alga and land plants.

Different from previous polysome or protein pulse labelling analyses, our genome-wide analyses enabled a comprehensive examination of regulation in chloroplast gene expression in response to defects in the assembly of specific protein complexes. Thereby, we revealed that severe PSII defects caused reduced expression of the PSI core subunit genes *psaA* and *psaB*, and to some extent also *psaC*—a regulation that seems to be achieved on both transcript and/or translational levels with differing contributions that depend on mutant background and respective PSII defect (Figs. 1c and 2c and Extended Data Figs. 3a and 5a). This regulation across complexes has been previously observed in embryophytes and Chlamydomonas (for example, refs. ^12,33–36,80^), and may play a role in adjusting PSII:PSI stoichiometry^81^. This could support the protection of PSI from irreversible photooxidative damage under conditions of decreased PSII assembly and/or repair^82^. In addition, our data indicate that the loss of Cyt *b*_6_*f* function in Δ*petN* and Δ*petB*/*D* mutants induced the downregulation of *psbA* translation (Extended Data Fig. 5e), an effect that has previously been observed in other Cyt *b*_6_*f* mutants^25,49,52^ and may also be caused by a cross-complex feedback regulation (note that all herein and also previously analysed Cyt *b*_6_*f* mutants have different primary and secondary defects but share a reduced Cyt *b*_6_*f* complex accumulation that is accompanied by reduced *psbA* translation; Extended Data Fig. 5e). Furthermore, many (but not all) mutants with severely compromised light reactions of photosynthesis exhibit reduced *rbcL* expression (Figs. 1g and 4g and Extended Data Figs. 2d, 8c and 9i), which may point to a regulatory connection between functional light reactions (or the resulting energy, pH and/or redox balances) and *rbcL* expression. This connection may simply act through a retrograde signalling-triggered downregulation of *RBCS*^83^ that could then control *rbcL* translation through CES (Fig. 7). Finally, our data indicate that severely impaired photosynthesis may induce the expression of some chloroplast biogenesis-related genes such as *rpoC1*, *rpoC2*, *ycf2*, *rpl33* and *rps18* (Figs. 2b, 3c and 5c and Extended Data Fig. 8), the reason for which remains to be determined.

In sum, our work revealed several plastid-autonomous, assembly-dependent regulatory processes in embryophytes that (1) feedback control the accumulation of subunits within a given photosynthetic protein complex, (2) can fine-tune the stoichiometry between photosynthetic complexes and (3) disclose similarities to and differences from the CES feedback regulation described in Chlamydomonas^10^. Such regulatory feedback loops of plastid-encoded proteins can explain evolutionary pressures that led to the preservation of certain genes within plastomes^6,7^. In future, the detailed elucidation of molecular mechanisms of the feedback regulation revealed in this work will require the identification of the *trans* factors and *cis* elements involved, and the analysis of their molecular mode of interaction.

## Methods

### Plant material and growth conditions

Depending on the extent of their photosynthetic deficiency, tobacco and Arabidopsis mutants were grown side by side with respective control plants either autotrophically on soil or heterotrophically on Murashige and Skoog (MS) medium supplemented with sugar. The growth conditions were adjusted according to the molecular defects of the mutants as detailed below and in Supplementary Tables 1 and 2.

### Cultivation of tobacco on soil (autotrophic growth)

Seeds were germinated in soil moisturized with water supplemented with 0.15% (v/v) Previcur fungicide and grown under 150 μmol photons m^−2^ s^−1^ for 16 h/8 h light/dark cycles at 22 °C/18 °C and 70% humidity. During the first 9 days, the seedlings were covered with a plastic cultivation dome. Eight days after sowing, seedlings were transplanted to individual pots (6 cm diameter) and kept for an additional 2 days under the same conditions before they were transferred to the conditions shown in Supplementary Table 2. After 3–4 weeks, at a developmental stage with 4 true leaves, the aerial parts of the plants were harvested and immediately flash frozen in liquid nitrogen. At this developmental stage, large parts of the plant are actively undergoing chloroplast biogenesis, including the assembly of photosynthetic complexes. The plants were harvested 30 min after the onset of light because chloroplast gene expression peaks early after the start of illumination^75^.

### Cultivation of tobacco on vermiculite (autotrophic growth)

For cotyledon-stage harvesting, seeds were sown directly on a nylon net placed on vermiculite and grown for 7 days under the conditions shown in Supplementary Table 2. The seedlings were harvested 30 min after the onset of light and immediately flash frozen in liquid nitrogen.

### Plant cultivation on sterile media (heterotrophic growth)

Tobacco knockout mutants of essential photosynthetic genes were grown heterotrophically in Magenta boxes containing agar-solidified MS medium supplemented with 3% sucrose under the conditions listed in Supplementary Table 2 (ref. ^84^). Tissue was harvested once the equivalent developmental stage of a 4-leaf-old tobacco seedling was reached. Arabidopsis *hcf173*-*2* and *hcf145*-*2* mutants were grown heterotrophically on MS medium with 1.5% sucrose and harvested after 4 weeks growth under the conditions mentioned in Supplementary Table 2.

### Cultivation of Arabidopsis on soil (autotrophic growth)

Arabidopsis was grown for 2 weeks after sowing on MS medium supplemented with 1.5% sucrose under long-day conditions (16 h light/8 h dark, ~100 µmol photons m^−2^ s^−1^ at 21 °C/16 °C and 60–75% humidity). Afterwards, the seedlings were transplanted into soil and kept in the same condition for 1 week before they were moved to the greenhouse (growth conditions given in Supplementary Table 2). Seedlings were harvested 4 weeks after sowing, before inflorescence.

### Generation of transplastomic mutant lines

Chloroplast transformation was performed as described in ref. ^85^. For cloning, plastome regions were amplified from genomic DNA, with ~400-bp flanks at both sides included to ensure homologous recombination into the plastid genome (Supplementary Fig. 12). An *aadA* cassette was inserted for transformant selection on spectinomycin. Biolistic bombardment of sterile tobacco leaves with gold particles coated with plasmid DNA was followed by a selection of spectinomycin-resistant cell lines^85^. Successful transformation of chloroplasts was confirmed by a test for resistance to the two aminoglycoside antibiotics spectinomycin and streptomycin. The chloroplast transformants were converted into a homoplastomic state by additional regeneration cycles under selection pressure. Homoplasmy was tested by segregation analysis of the next generation on spectinomycin as well as restriction fragment length polymorphism (RFLP) analyses.

For Δ3’-*psbD*, a DNA fragment flanked by EcoRV and SpeI restriction sites (position 32,747–38,311) was amplified and cloned into pBS KS vector (Supplementary Fig. 12a). The region from 35,211–36,464 was removed from the subclone using the restriction enzymes BamHI and BstEII, and replaced by the *aadA* cassette. SpeI was selected as enzyme for the RFLP analysis. This resulted in a fragment size of 6.8 kb and 4.6 kb for wild-type and transplastomic chloroplast genomes, respectively.

For Δ*petB/D*, a DNA fragment flanked by HindIII and SalI restriction sites (position 72,710–82,121) was cloned into pUC19 vector (Supplementary Fig. 12b). The region from 74,952–79,334 was removed from the subclone using the restriction enzymes NcoI and PstI, and replaced by the *aadA* cassette. SalI was selected as enzyme for the RFLP analysis. This resulted in a fragment size of 11.3 kb and 8.1 kb for wild-type and transplastomic chloroplast genomes, respectively.

The Δ*psaB* mutant alleles were created as follows (Supplementary Fig. 12c): for Δ*psaB*-2, the vector described in ref. ^66^ was modified by inserting the point mutations shown in Extended Data Fig. 9e. For Δ*psaB*-1, the vector used to create Δ*psaB*-2 was digested with BamHI, followed by Klenow fill-in to generate blunt ends. This resulted in a frameshift and a premature stop codon ~0.4 kb downstream of the start codon (Δ*psaB*-1) (Extended Data Fig. 9a). BglI and StyI were used as RFLP enzymes, resulting in a 2.4-kb fragment in the wild type and a 2.5-kb band for the transplastomic lines.

For Δ*atpB/E*, a DNA fragment flanked by KpnI and HindIII restriction sites (position 52,377–58,584) was cloned into pUC19 vector (Supplementary Fig. 12d). The region from 55,112–56,719 was removed from the subclone using the restriction enzymes SmaI and HpaI, and replaced by the *aadA* cassette. NdeI was used as enzyme for the RFLP analysis. This resulted in a fragment size of 4.6 kb and 4.2 kb for wild-type and transplastomic chloroplast genomes, respectively.

For Δ*ndhC/K/J*, a DNA fragment flanked by SalI and SacI restriction sites (position 49,842–56,047) was cloned into pUC19 vector (Supplementary Fig. 12e). The region from 51,365–52,423 was removed from the subclone using the restriction enzymes AvaI and NcoI, and replaced by the *aadA* cassette. SpeI was selected as enzyme for the RFLP analysis. This resulted in a fragment size of 7 kb and 6.5 kb for wild-type and transplastomic chloroplast genomes, respectively.

For *petA*-ox-Δ*cemA*, a DNA fragment flanked by SpeI and SnaBF restriction sites (position 62,543–64,894) was cloned into pBS SK+ vector (Supplementary Fig. 12f). The region from 63,521–63,873 was removed from the subclone using the restriction enzymes BstEII and HindIII, and replaced by the *aadA* cassette. BstBI was selected as enzyme for the RFLP analysis. This resulted in a fragment size of 1.3 kb and 2.2 kb for wild-type and transplastomic chloroplast genomes, respectively.

### Microarray-based ribosome and transcriptome profiling

Ribosome footprints and total RNA isolation, labelling and hybridization to custom tiling microarrays were performed as described in ref. ^12^. Data analysis was performed as described previously^12^, with minor modifications. Briefly, the data were analysed with GenePix Pro 7.2 software. Low-quality spots on the microarray were manually removed after visual inspection. Only probes for which at least two out of four technical replicate spots showed sufficient quality were considered in the analysis and are represented in the figures. The background was subtracted using the local subtraction method. Afterwards, the median value from technical replicates was calculated for each probe for both channels (F635-B635 or F532-B532; Supplementary Data 1) with values ≤100 considered below background and set to zero. The signal of all probes covering protein-coding regions in ribosome and transcriptome profiling were normalized to the arbitrary value of 4,000 to exclude biases caused by technical variations, such as different overall labelling and hybridization efficiencies between replicates and different experiments. Subsequently, the average value of normalized probe signals in each reading frame was calculated. Next, the relative abundances of ribosome footprints and total RNA were calculated by normalizing the average signal of each reading frame to the average signal of all reading frames on a logarithmic scale. To compare the relative changes in the transcript accumulation and translation output between mutants and controls, the log_2_-transformed relative abundance values of the ribosome footprints and the total RNA in the control were subtracted from the corresponding relative abundance values in the mutant for each biological replicate and each reading frame. The average of relative changes of ribosome footprints (RF, translation output) and total RNA were calculated for each reading frame on the basis of three biological replicates. Translation efficiencies were calculated as the ratio of changes in translation output and RNA levels (Supplementary Data 1 and Fig. 3). Significance was assessed using the empirical Bayes method in the limma package^86^ and the *P* values were adjusted according to the false discovery rate (FDR) procedure^87^. The applied cut-off of 2-fold changes to define substantially altered gene expression is justified by the 5th percentile in the distribution of fold changes across all microarray data (Supplementary Fig. 2). Whole chloroplast genome microarray data are shown as volcano plots throughout the manuscript.

### NGS-based ribosome and transcriptome profiling

For Ribo-seq, the ribosome footprints were isolated as described in ref. ^88^ with some minor modifications. RNase I was applied at 250 U ml^−1^ of plant lysate (derived from 100 mg of plant fresh weight) for the generation of ribosome footprints. The RNase I-treated lysate was layered on a sucrose cushion to purify monosomes. Ribosome footprints from ~20 nt to ~39 nt were size selected from polyacrylamide gels. Ribosomal (r)RNA fragments were removed with biotinylated DNA oligonucleotides as described in ref. ^88^. Libraries were prepared as previously described^88^. For total RNA samples, RNA was isolated with the TRIzol Reagent (Thermo Fisher) according to manufacturer instructions, co-purified genomic DNA was removed using TURBO DNase and libraries were prepared using Zymo-Seq RiboFree total RNA library kit according to manufacturer instructions. Note that we have verified that the enzymatic rRNA depletion of the Zymo-Seq RiboFree total RNA library kit does not affect the NGS-based quantification of abundant chloroplast mRNAs (for example, *psbA* and *rbcL*).

### NGS data analysis

The quality of the obtained footprint reads was checked with FastQC (http://www.bioinformatics.babraham.ac.uk/projects/fastqc/). Adapters were removed using cutadapt^89^. All alignments were performed with STAR aligner (v.2.7.1a)^90^, using a sequential mapping approach similar to that described in ref. ^11^. Reads were sequentially mapped to (1) rRNA and transfer (t)RNA contaminants, (2) chloroplast genome, (3) mitochondrial genome and (4) nuclear genome^91^. Only unmapped reads from the previous alignment(s) were used as input for succeeding alignment(s). This sequential mapping approach prevents misalignment of genuine chloroplast reads to inactive copies of chloroplast genes in mitochondrial or nuclear genomes. The following sequences were obtained from NCBI (with following annotations): 5.8S rRNA (AJ300215.1), 5S rRNA (AJ222659.1), 18S rRNA (AJ236016.1) and 26S rRNA (AF479172.1), chloroplast genome (Z00044.2), mitochondrial genome (NC_006581.1). Sequences for the nuclear genome and tRNAs were obtained from solgenomics (ftp://ftp.solgenomics.net/genomes/Nicotiana_tabacum/). Additional processing and sorting of alignment data were done using samtools (v.1.11)^92^ and bedtools (v.2.30.0)^93^. Reads mapping to the protein-coding region (CDS) of genes were summarized using featureCounts^94^. For chloroplast data, reads mapping to the first 10 and the last 30 nucleotides of the CDS that derive from initiating and terminating ribosomes were excluded (due to the much slower dynamics of initiation and termination compared with elongation). Chloroplast-encoded intron-containing genes were given special treatment since unspliced mRNAs make up a certain fraction of the RNA pool and these mRNAs can undergo translation that potentially terminates in introns^25^. For Ribo-seq data, only reads mapping to the last exon were counted. In the RNA-seq data, reads spanning intron–exon junctions by more than 52 bp were excluded as they reflect unspliced mRNA. The averages of relative changes in ribosome footprints (translation output) and total RNA were calculated for each reading frame on the basis of three biological replicates. Translation efficiencies were calculated as the ratio of changes in translation output and RNA levels (Supplementary Data 1 and Fig. 3). The applied cut-off of 4-fold changes to define substantially altered gene expression is justified by the 5th percentile in the distribution of fold changes across all NGS data (Supplementary Fig. 2). Whole chloroplast genome NGS data are shown as volcano plots throughout the manuscript.

### Protein extraction and immunoblot analysis

Total protein was extracted according to ref. ^95^. Depending on the follow-up experiment (described below), the separation of proteins by SDS–PAGE was performed according to either Laemmli^96^ or Schägger^97^. For western blot, 5 μg protein was mixed with 2× SDS protein sample buffer (125 mM Tris-HCl pH 6.8, 4% (w/v) SDS, 20% (v/v) glycerol, 25 mM EDTA pH 8.0, 0.04% (w/v) bromophenol blue, 2% (v/v) 2-mercaptoethanol), denatured for 10 min at 70 °C and loaded onto 0.75-mm thick 4–20% (w/v) precast polyacrylamide gradient gels. For in vivo protein pulse labelling experiments, tricine gels were used according to Schägger^97^. Protein samples were mixed with 2× tricine sample buffer (100 mM Tris-HCl pH 6.8, 24% glycerine (v/v), 12.5% SDS (w/v), 0.02% Coomassie brilliant blue G250 (w/v), 2% (v/v) 2-mercaptoethanol), denatured for 10 min at 70 °C and separated on 1-mm thick 4–10% tris-tricine gradient gels. Gel-separated proteins were transferred to nitrocellulose membranes in wet blot tanks. Ponceau S staining served as loading control. For attempts to detect the truncated PsbD protein (Supplementary Fig. 7), both abovementioned gel systems were tested in combination with nitrocellulose and PVDF membranes. All used antibodies are listed in Supplementary Table 4.

### Thylakoid isolation and BN–PAGE

Thylakoid isolation and BN–PAGE were performed as described in ref. ^49^.

### In vivo pulse labelling of chloroplast proteins

^35^S pulse labelling of newly synthesized chloroplast proteins was done in tobacco plants at two different developmental stages: 3–4-week-old seedlings (4-true-leaf stage) (Extended Data Fig. 1c and Supplementary Fig. 11) and 8–12-day-old seedlings (cotyledon stage) (Fig. 1d and Supplementary Fig. 6). The experiment was performed as follows: 12 leaf discs (0.5-cm diameter) of the 4-true-leaf-stage plants or 25 seedlings of the cotyledon stage plants were soaked in 400 μl labelling buffer supplemented with 440 μCi of EasyTagTM EXPRESS35S Protein Labelling Mix (PerkinElmer; 11 mCi ml^−1^, >1,000 Ci mmol^−1^; ^35^S-labelled methionine and cysteine) supplemented with 20 μg ml^−1^ cycloheximide. The samples were vacuum infiltrated three times for 20 s, followed by incubation for 20 min under ~80 μmol photons m^−2^ s^−1^ light intensity. Total proteins were extracted as described in ref. ^95^ and either directly loaded on gels or separated into soluble and thylakoid membrane proteins by pelleting the thylakoid membrane via centrifugation for 15 min at 13,000 *g*. To obtain a simplified pattern of labelled proteins, cytosolic translation was blocked by addition of cycloheximide. ^35^S-labelled proteins that equal 500,000 cpm (counts per minute) for total proteins, 1,000,000 cpm for soluble proteins or 100,000 cpm for thylakoid membrane proteins were separated on tricine gels. Coomassie blue staining was used as loading control for steady-state protein amounts. Signals of abundant de novo synthesized proteins were detected by autoradiography using Typhoon TRIO+ Variable Mode Imager (Amersham Biosciences).

For the pulse-immunoprecipitation assay, an equivalent of 200,000 cpm of the labelled membrane proteins was used for immunoprecipitation with antibodies against PetA and PetB. Precipitated proteins were separated by SDS–PAGE and detected by autoradiography as described above.

### Immunochase

Leaf discs were submerged in liquid MS media including 500 µg ml^−1^ lincomycin (+lincomycin; to switch off chloroplast protein synthesis) or excluding lincomycin (−lincomycin) and vacuum infiltrated three times for 20 s. Following an overnight incubation at 4 °C, the leaf discs were transferred to the following conditions: 16 h light at 350 µmol photons m^−2^ s^−1^ light intensity and 8 h dark, at 22 °C and 18 °C, respectively. Tissue was sampled at the following time points: 0 min, 2 h, 6 h, 12 h, 1 d, 2 d and 4 d. Total proteins were extracted with phenol and quantified using the PIERCE BCA Protein Assay kit (Thermo Fisher). Protein gel blot analyses to detect PetA, PetB and PsbA accumulation were performed as described above.

### RNA and DNA gel blot analyses

RNA was isolated using TRIzol Reagent following manufacturer instructions (Thermo Fisher) and the pellet was resuspended in 20 μl RNase-free water. Gel electrophoresis and gel blotting were performed with 2 µg RNA as described previously^98^. Primers used for generating probes for RNA gel blot analysis are listed in Supplementary Table 5.

DNA gel blot analyses were performed as previously described^99^. Genomic DNA (3 µg) was digested with the abovedescribed restriction enzyme(s) and size separated by electrophoresis on 0.8% agarose gel. Primers for generating the probe for *psaB* DNA gel blot analysis are listed in Supplementary Table 5. Probes for all other DNA gel analyses were generated by restriction digestion of chloroplast transformation vectors using the enzymes shown in Supplementary Fig. 12.

### Reporting summary

Further information on research design is available in the Nature Portfolio Reporting Summary linked to this article.

## Supplementary information

Supplementary Information Supplementary Results, Tables 1–5, Figs. 1–12 and References.

Reporting Summary

Supplementary Data 1 Ribosome profiling and transcript profiling data and statistics.

Supplementary Data 2 Source data for supplementary figures.

## Source data

Source Data Fig. 1 Statistical source data and unprocessed pulse labelling and northern blots.

Source Data Fig. 3 Unprocessed western blots.

Source Data Fig. 4 Unprocessed pulse labelling blots.

Source Data Fig. 6 Unprocessed western and northern blots.

Source Data Extended Data Fig. 1 Statistical source data and unprocessed pulse labelling blots.

Source Data Extended Data Fig. 4 Unprocessed western blots.

Source Data Extended Data Fig. 6 Unprocessed western blots.

## Data Availability

Ribosome profiling and transcriptome profiling data are available under the GEO accession GSE286011. Statistics are detailed in Supplementary Data 1. Source data are provided with this paper.
