# Supplementary material for: Assembly-dependent translational feedback regulation of photosynthetic proteins in land plants
Source: Nat Plants. 2025 Aug 18;11(9):1920–38. doi: 10.1038/s41477-025-02074-x (PMC12449265; doi:10.1038/s41477-025-02074-x)
Supplement: Supplementary file 1 — Supplementary Results, Tables 1–5, Figs. 1–12 and References. [file 41477_2025_2074_MOESM1_ESM.pdf]

nature plants

Supplementary information https://doi.org/10.1038/s41477-025-02074-x

Assembly-dependent translational feedback regulation of photosynthetic proteins in land plants

In the format provided by the authors and unedited

Supplemental Material

Assembly-dependent translational feedback regulation of photosynthetic proteins in land plants

Rabea Ghandour[1*], Yang Gao[1], Stephanie Ruf[1], Ralph Bock[1], Reimo Zoschke[1#]

[1]Max Planck Institute of Molecular Plant Physiology, Potsdam-Golm, Germany

*Current address:

Friedrich Schiller University, Institute of Microbiology, Jena, Germany

[#]Corresponding author:

Reimo Zoschke

E-mail: zoschke@mpimp-golm.mpg.de

Phone: +49 331 5678375

Max Planck Institute of Molecular Plant Physiology

Am Mühlenberg 1

14476 Potsdam-Golm, Germany

E-mail addresses of co-authors:

Rabea Ghandour	rabea.ghandour@uni-jena.de
Yang Gao	ygao@mpimp-golm.mpg.de
Stephanie Ruf	sruf@mpimp-golm.mpg.de
Ralph Bock	rbock@mpimp-golm.mpg.de

This supplement contains:

Supplemental Results

Tables S1-S4

Figures S1-S12

Supplemental Material Table of Contents

APPENDIX

Supplemental Results **4**

Ribosome profiling readily detects translational feedback regulation in Rubisco 4

No evidence for CES in the chloroplast NDH complex 5

Supplemental Tables **7**

Table S1: Overview of the analyzed tobacco and Arabidopsis mutants 7

Table S2: Growth conditions of the analyzed mutants 13

Table S3: Sequence overview of Δ*psaB* mutant alleles 15

Table S4: Overview of used antibodies 18

Table S5: Overview of used primers 19

Supplemental Figures **20**

Fig. S1. Summary of the previously known translational feedback regulation during the assembly of photosynthetic protein complexes in Chlamydomonas. 20

Fig. S2. Fold change distribution of all translatome and transcriptome data. 21

Fig. S3 Comparison of translation efficiencies for chloroplast reading frames in each mutant analyzed in this study. 22

Fig. S4. Phenotypes of *psbD* mutants. 24

Fig. S5. *psbA* transcript accumulation defect and PSI and PSII gene expression in the *hcf173-2* mutant. 25

Fig. S6. Pulse labeling showing the reduction of *de novo* synthesized PsbA in KD-*psbD* at the cotyledon stage. 26

Fig. S7. Attempts to detect a C-terminally truncated PsbD protein in the Δ*3'-psbD* mutant. 27

Fig. S8. PSII complex assembly defects in KD-*psbD* and Δ3'-*psbD* mutants. 29

Fig. S9. Western blot analysis to determine PetA accumulation in the Δ*petB/D* mutant. 30

Fig. S10. Comparative determination of PetA and PetB turnover in tobacco. 31

Fig. S11. Pulse-IP of PetA and PetB in the *petA*-ox-Δ*psaI* mutant. 33

Fig. S12. Cloning strategies of chloroplast transformation vectors and restriction fragment length polymorphism (RFLP) analyses of mutants that were not previously described. 34

References **36**

Supplemental Results

Ribosome profiling readily detects translational feedback regulation in Rubisco

We reasoned that ribosome profiling[1,2] should enable the efficient transcriptome-wide detection of translational feedback regulation in mutants with impaired photosynthetic complex assembly. To validate this assumption, we aimed for a confirmation of CES involved in Rubisco assembly by ribosome profiling[3,4]. Rubisco consists of eight small subunits, encoded by the nuclear *RBCS* gene family and eight large subunits, encoded by the single-copy chloroplast gene *rbcL* (Extended Data Fig. 1*A*). CES in Chlamydomonas and tobacco adjusts translation of *rbcL* according to the availability of RBCS (Extended Data Fig. 1*A*)[4,5].

A tobacco *RBCS* antisense RNA mutant (as-*RBCS*) was chosen for analysis and exhibited a pale green, growth-impaired phenotype caused by a more than 50% reduction of RBCS levels that was accompanied by a more than 50% decrease of RbcL levels (Extended Data Fig. 1*A*-*C*), which is consistent with the previous mutant description[6]. ^{35}S-radiolabeling of newly synthesized chloroplast proteins confirmed the expected reduction in RbcL protein synthesis (Extended Data Fig. 1*C* and *D*). Next, chloroplast gene expression in as-*RBCS* was examined by microarray-based chloroplast ribosome profiling[2]. Average ribosome footprint and transcript abundances (as proxy for translation output and mRNA accumulation) were determined and relative changes between mutant and control lines were calculated for each chloroplast reading frame[2] (Extended Data Fig. 1*E*). Since each ribosome footprint is generated by an elongating ribosome, average footprint abundances reflect translation output in a given reading frame and thereby provide a proxy for protein synthesis levels. In addition, transcript levels were determined to distinguish between regulation on translation and transcript levels, both of which can adjust translation output. Ribosome profiling revealed a significant, more than elevenfold reduction in ribosome coverage of the *rbcL* transcript in as-*RBCS* (Extended Data Fig. 1*E*, Supplementary Data 1). At the same time, the *rbcL* transcript level was significantly but only threefold reduced (Extended Data Fig. 1*E*). Together, this demonstrates that *rbcL* translation is substantially diminished when RBCS levels are reduced, thereby validating previous findings[4] (Extended Data Fig. 1*C*). Our calculations of translation efficiencies (translation output normalized to transcript levels) validate these findings (Fig. S3).

Together these data confirm the known CES in tobacco Rubisco assembly[4,5] and demonstrate that ribosome profiling provides an efficient tool to screen for assembly-dependent feedback regulation.

No evidence for CES in the chloroplast NDH complex

The non-essential chloroplast NDH complex is specific for embryophytes and absent in Chlamydomonas and other algae, suggesting a role in adaptation of photosynthesis to land colonization[7]. The NDH complex consists of at least 25 subunits (Extended Data Fig. 10*A*, in tobacco 11 of them are plastid-encoded; NdhA/B/C/D/E/F/G/H/I/J/K), which are homologous to the genes of the mitochondrial respiratory complex I, and the NDH complex forms a supercomplex with PSI that mediates cyclic electron transport beside other functions[8-11]. There is still little known about the assembly of the chloroplast NDH complex[9-12].

However, to obtain first insights into potential feedback regulation in NDH complex assembly, a Δ*ndhC*/*K*/*J* mutant was examined by ribosome profiling[13] (Extended Data Fig. 10*B*-*E*, Supplementary Data 1). The chloroplast NDH complex is not essential and the knockout of NDH subunits has no phenotypic consequences (Extended Data Fig. 10*B*). In Δ*ndhC*/*K*/*J*, the *ndhK* reading frame is completely deleted, causing reduced transcript accumulation and translation output (Extended Data Fig. 10*C*-*E*), though, due to the naturally low transcript levels of *ndhK*, the fold change is mild. *ndhC* is only truncated at its 3' end in Δ*ndhC*/*K*/*J* and, accordingly, overall *ndhC* transcript accumulation and translation were largely unaffected (Extended Data Fig. 10*D* and *E*). The *ndhJ* 5' end is deleted in Δ*ndhC*/*K*/*J*. Due to its start codon deletion, *ndhJ* translation output was decreased and its transcript over-accumulated (certainly caused by transcriptional read-through from upstream sense direction-inserted *aadA*)[14]. Apart from these primary defects in gene expression, no additional, more than twofold significant changes were observed in the expression of NDH genes, suggesting the absence of measurable feedback regulation downstream of NdhK and NdhJ synthesis and/or accumulation.

In addition, we analyzed a Δ*ndhA*/*I* mutant (Extended Data Fig. 10*F*-*I*)[15]. In Δ*ndhA*/*I*, the *ndhA* and *ndhI* reading frames are completely deleted, causing the observed significantly reduced transcript accumulation and translation output of these genes (Extended Data Fig. 10*H* and *I*). As expected, the deletion of the very 3' end of the *ndhH* gene has little consequences for its expression levels (Extended Data Fig. 10*I*). However, we observed an approximately twofold decrease for *ndhE* and *ndhG* transcript accumulation and a mild reduction in *ndhH* RNA accumulation all of which caused an equally pronounced diminution in the translation output of the respective reading frames (Extended Data Fig. 10*I*). Based on data from Arabidopsis and maize we expect that transcription of the tobacco *ndhH*, *ndhA*, *ndhI*, *ndhG* and *ndhE* genes is driven by promoters that are located upstream of *rps15*, *ndhH* and *ndhA*, respectively (Extended Data Fig. 10*G*)[16-19]. Transcription from these promoters is likely strongly diminished by *aadA* transcription in antisense direction (Extended Data Fig. 10*G*), similar to the *aadA* antisense effect impairing *psbD* transcription and transcript

accumulation in the KD-*psbD* mutant (Fig. 1*A*-*C*). Consequently, *rps15*, *ndhH*, *ndhG* and *ndhE* transcript accumulation and translation output are reduced in Δ*ndhA*/*I* in addition to the defects of the deleted *ndhA* and *ndhI* genes (Extended Data Fig. 10*H* and *I*). In contrast, the transcription of the downstream *psaC* and *ndhD* genes is unaffected because they are served by an additional promoter that is located downstream of the *aadA* insertion site and upstream of *psaC* (Extended Data Fig. 10*G*)[16,17,20]. In sum, the observed transcript accumulation and translation output defects for *rps15*, *ndhH*, *ndhA*, *ndhI*, *ndhG* and *ndhE* are certainly a direct consequence of the *aadA* transgene insertion and antisense transcription induced by the *aadA* gene. Beside these primary defects, we did not detect any additional significant or more than twofold change in the expression of other NDH genes (Extended Data Fig. 10*H* and *I*). For all NDH genes whose expression is not directly affected by the *aadA* insertion in Δ*ndhA*/*I* (and CES could therefore not be verified), this suggests the absence of significant feedback regulation downstream of the knocked out NdhA or NdhI subunits.

Supplemental Tables

Table S1: Overview of the analyzed tobacco and Arabidopsis mutants

Analyzed photosynthetic mutants ordered by protein complexes.

Complex impaired in assembly	Mutant	Species	Type of mutation	Gene localization	Function of the gene	Correspon-ding control	Analyzed by	Autotrophic growth	References
**Rubisco**	as-*RBCS*	*Nicotiana tabacum*	*RBCS* antisense-RNA	Nucleus	Small subunit of Rubisco	SR1	Microarray	+	[6,21]
**PSII**	KD-*psbD*	*Nicotiana tabacum*	5' UTR insertion transcriptional knockdown	Chloroplast	Essential core subunit, PsbD, of PSII	*aadA* control (pRB8c)	Illumina Sequencing	+	[22,23]
	hcf173-2	*Arabidopsis thaliana*	T-DNA insertion	Nucleus	RNA-binding protein, stabilization/translation factor of *psbA* mRNA	Wild type Col0	Microarray	-	[24]

	Δ3'-*psbD*	*Nicotiana tabacum*	Insertion knockout	Chloroplast	Essential core subunits, PsbD (D2) and PsbC (CP43) of PSII	*aadA* control (pRB8c)	Illumina Sequencing	-	[13,23]
	Δ*psbJ*	*Nicotiana tabacum*	Insertion knockout	Chloroplast	Essential subunit of PSII, PsbJ	*aadA* control (pRB8c)	Illumina Sequencing	-	[23,25]
**Cyt b_6f**	Δ*petB*/*D*	*Nicotiana tabacum*	Insertion knockout	Chloroplast	Essential core subunits, PetB and PetD, of Cyt b_6f, and PsbB, PsbT, PsbN and PsbH subunits from PSII	*aadA* control (pRB8c)	Illumina Sequencing	-	[13,23]
	petA-ox-Δ*psaI*	*Nicotiana tabacum*	Insertion knockout of non-essential PSI gene *psaI* causing overexpress	Chloroplast	Non-essential subunit of PSI (overexpressed gene *petA* encodes the essential subunit cytochrome *f* of the Cyt b_6f complex)	*aadA* control (pRB8c)	Microarray	+	[23,26]

			ion of downstream *petA*						
	petA-ox-Δ*cemA*	*Nicotiana tabacum*	Insertion knockout of the non-essential *cemA* gene causing overexpression of downstream *petA*	Chloroplast	Non-essential envelope membrane protein (overexpressed gene *petA* encodes the essential subunit cytochrome *f* of the Cyt b_6f complex)	*aadA* control (pRB8c)	Microarray	+	[23,27]
	petA-ox-Δ*ycf4*	*Nicotiana tabacum*	Insertion knockout of the non-essential PSI assembly factor Ycf4 causing overexpress	Chloroplast	non-essential PSI assembly factor (overexpressed gene *petA* encodes the essential subunit cytochrome *f* of the Cyt b_6f complex)	*aadA* control (pRB8c)	Microarray	+	[23,28]

			ion of downstream *petA*						
	Δ*petN*	*Nicotiana tabacum*	Insertion knockout	Chloroplast	Essential subunit for assembly of Cyt b_6f	*aadA* control (pRB8c)	Illumina Sequencing	-	[13,23]
**PSI**	Δ*psaB*	*Nicotiana tabacum*	Frame-shift mutation causing premature translation termination	Chloroplast	Essential core subunit, PsaB, of PSI	*aadA* control (pRB70)	Illumina Sequencing	-	[29,30], this work
	KD-*psaA*	*Nicotiana tabacum*	Shine-Dalgarno mutation (knockdown)	Chloroplast	Essential core subunit, PsaA, of PSI	*aadA* control (aadA cassette inserted at the same location as the mutant)	Microarray	+	[28]

	hcf145-2	*Arabidopsis thaliana*	T-DNA insertion	Nucleus	RNA-binding protein, stabilization/translation factor of *psaA/B*, *rps14*	Wild type Col0	Microarray	-	[31,32]
	psaD1-1	*Arabidopsis thaliana*	T-DNA insertion (knockout of one of two gene copies encoding PSAD)	Nucleus	Essential stromal subunit of PSI (PSI accumulation reduced to ~40 % of the wild type)	Wild type Col0	Microarray	+	[33]
**NDH**	Δ*ndhC*/*K*/*J*	*Nicotiana tabacum*	Insertion knockout	Chloroplast	NdhC subunit from the subcomplex M and NdhK and NdhJ subunits from subcomplex A of the non-essential NDH complex	*aadA* control (pRB8c)	Microarray	+	[13,23]
	Δ*ndhA*/*I*	*Nicotiana tabacum*	Insertion knockout	Chloroplast	NdhA subunit from the subcomplex M and NdhI subunit	Wild type	Microarray	+	[15]

					from subcomplex A of the non-essential NDH complex				
**ATP synthase**	Δ*atpB*/*E*	*Nicotiana tabacum*	Insertion knockout	Chloroplast	Essential β-subunit and ε-subunit of ATP synthase	*aadA* control (pRB8c)	Illumina Sequencing	-	[13,23]
	as-*ATPC* (line atpC1)	*Nicotiana tabacum*	*ATPC* antisense-RNA	Nucleus	Essential γ-subunit of ATP synthase (*ATPC* transcript accumulation and translation output reduced to ~10% of the wild type, ATP synthase level reduced to ~25%, Fig. 6*F*)	wild type SNN	Illumina Sequencing	+	[34]

Table S2: Growth conditions of the analyzed mutants

**Complex**	**Mutant**	**Growth condition**
Rubisco	as-*RBCS*	Autotrophic growth (see Material and Methods).<br>150 µmol photons m^{-2} s^{-1} for 16h/8 h light/dark at 22°/18° C and 70% humidity, then transferred to 350 µmol photons m^{-2} s^{-1} for 16h/8 h light/dark at 22°/18° C and 75/70% humidity.
PSII	KD-*psbD*	Autotrophic growth (see Material and Methods).<br>150 µmol photons m^{-2} s^{-1} for 16h/8 h light/dark at 22°/18° C and 70% humidity, then transferred to 350 µmol photons m^{-2} s^{-1} for 16h/8 h light/dark at 22°/18° C and 75/70% humidity.
	hcf173-2	Heterotrophic growth (see Material and Methods).<br>25 µmol photons m^{-2} s^{-1} for 16h/8 h light/dark at 22°/18° C.
	Δ3'-*psbD/C*	Heterotrophic growth (see Materials and Methods).<br>5-10 µmol photons m^{-2} s^{-1} for 16h/8 h light/dark at 22°/18° C.
	Δ*psbJ*	Heterotrophic growth (see Material and Methods).<br>5-10 µmol photons m^{-2} s^{-1} for 16h/8 h light/dark at 22°/18° C.
Cyt b_6f	Δ*petB/D*	Heterotrophic growth (see Material and Methods).<br>5-10 µmol photons m^{-2} s^{-1} for 16h/8 h light/dark at 22°/18° C.
	petA-ox-Δ*psaI*	Autotrophic growth (see Material and Methods).<br>150 µmol photons m^{-2} s^{-1} for 16h/8 h light/dark at 22°/18° C and 70% humidity then transferred to 350 µmol photons m^{-2} s^{-1} for 16h/8 h light/dark at 22°/18° C and 75/70% humidity.
	petA-ox-Δ*cemA*	
	petA-ox-Δ*ycf4*	Autotrophic growth (see Material and Methods).<br>50 µmol photons m^{-2} s^{-1} for 16h/8 h light/dark at 25°/22° C then transferred to 70 µmol photons m^{-2} s^{-1} for 16h/8 h light/dark at 22°/18° C and 75/70% humidity.
	Δ*petN*	Heterotrophic growth (see Material and Methods).<br>5-10 µmol photons m^{-2} s^{-1} for 16h/8 h light/dark at 22°/18° C.

PSI	Δ*psaB*	Heterotrophic growth (see Material and Methods).<br>5-10 µmol photons m^{-2} s^{-1} for 16h/8 h light/dark at 22°/18° C.
	KD-*psaA*	Autotrophic growth (see Material and Methods).<br>150 µmol photons m^{-2} s^{-1} for 16h/8 h light/dark at 22°/18° C and 70% humidity.
	hcf145-2	Heterotrophic growth (see Material and Methods).<br>25 µmol photons m^{-2} s^{-1} for 16h/8 h light/dark at 22°/18° C.
	psaD1-1	Autotrophic growth (see Material and Methods).<br>120 µmol photons m^{-2} s^{-1} for 16h/8 h light/dark at 25°/20° C (greenhouse).
NDH	Δ*ndhC*/*K*/*J*	Autotrophic growth (see Material and Methods).<br>150 µmol photons m^{-2} s^{-1} for 16h/8 h light/dark at 22°/18° C and 70% humidity.
	Δ*ndhA*/*I*	
ATP synthase	Δ*atpB*/*E*	Heterotrophic growth (see Material and Methods).<br>5-10 µmol photons m^{-2} s^{-1} for 16h/8 h light/dark at 22°/18° C.
	as-*ATPC*	Autotrophic growth (see Material and Methods).<br>150 µmol photons m^{-2} s^{-1} for 16h/8 h light/dark at 22°/18° C and 70% humidity then transferred to 350 µmol m^{-2} s^{-1} light for 16h/8 h light/dark at 22 °/18 °C and 75/70% humidity.

Table S3: Sequence overview of Δ*psaB* mutant alleles

WT	ATG GCA TTA CGA TTT CCA AGG TTT AGC CAA GGC TTA GCT CAG GAC CCC ACT ACT CGT CGT ATT TGG TTT GGT
Δ*psaB*-1	ATG GCA TTA CGA TTT CCA AGG TTT AGC CAA GGC TTA GCT CAG GAC CCC ACT ACT CGT CGT ATT TGG TTT GGT
Δ*psaB*-2	ATG GCA TTA CGA TTT CCA AGG TTT AGC CAA GGC TTA GCT CAG GAC CCC ACT ACT CGT CGT ATT TGG TTT GGT
	Start
WT	ATT GCT ACC GCA CAT GAC TTC GAG AGT CAT GAT GAT ATT ACT GAG GAA CGT CTT TAT CAG AAT ATT TTT GCT
Δ*psaB*-1	ATT GCT ACC GCA CAT GAC TTC GAG AGT CAT GAT GAT ATT ACT GAG GAA CGT CTT TAT CAG AAT ATT TTT GCT
Δ*psaB*-2	ATT GCT ACC GCA CAT GAC TTC GAG AGT CAT GAT GAT ATT ACT GAG GAA CGT CTT TAT CAG AAT ATT TTT GCT
WT	TCT CAC TTT GGT CAA TTA GCA ATA ATT TTT CTG TGG ACT TCC GGA AAT CTG TTT CAT GTA GCT TGG CAA GGA
Δ*psaB*-1	TCT CAC TTT GGT CAA TTA GCA ATA ATT TTT CTG TGG ACT TCC GGA AAT CTG TTT CAT GTA GCT TGG CAA GGA
Δ*psaB*-2	TCT CAC TTT GGT CAA TTA GCA ATA ATT TTT CTG TGG ACT TCC GGA AAT CTG TTT CAT GTA GCT TGG CAA GGA
WT	AAT TTT GAG TCG TGG GTA CAG GAC CCT TTA CAT GTA AGA CCT ATT GCT CAT GCA ATT TGG GAT CCT CAT TTT
Δ*psaB*-1	AAT TTT GAG TCG TGG GTA CAG GAC CCT TTA CAT GTA AGA CCT ATT GCT CAT GCA ATT TGG GAT CGA TCC TCA
Δ*psaB*-2	AAT TTT GAG TCG TGG GTA CAG GAC CCT TTA CAT GTA AGA CCT ATT GCT CAT GCA ATT TGG GAT CCT CAT TTT
WT	GGT CAA CCG GCC GTG GAA GCT TTT ACT CGA GGG GGT GCT CTT GGC CCA GTG AAT ATC GCT TAT TCT GGT GTT
Δ*psaB*-1	TTT TGG TCA ACC GGC CGT GGA AGC TTT TAC TCG AGG GGG TGC TCT TGG CCC AGT GAA TAT CGC TTA TTC TGG
Δ*psaB*-2	GGT CAA CCG GCC GTG GAA GCT TTT ACT CGA GGG GGT GCT CTT GGC CCA GTG AAT ATC GCT TAT TCT GGT GTT
WT	TAT CAG TGG TGG TAT ACA ATC GGT TTA CGC ACT AAT GAA GAT CTT TAT ACT GGT GCT CTT TTT CTA TTA TTT
Δ*psaB*-1	TGT TTA TCA GTG GTG GTA TAC AAT CGG TTT ACG CAC TAA Stop
Δ*psaB*-2	TAT CAG TGG TGG TAT ACA ATC GGT TTA CGC ACT AAT GAA GAT CTT TAT ACT GGT GCT CTT TTT CTA TTA TTT
WT	CTT TCT GCC ATA TCC TTA ATA GCA GGT TGG TTA CAC CTA CAA CCG AAA TGG AAA CCG AGC GTT TCC TGG TTC
Δ*psaB*-1	
Δ*psaB*-2	CTT TCT GCC ATA TCC TTA ATA GCA GGT TGG TTA CAC CTA CAA CCG AAA TGG AAA CCG AGC GTT TCC TGG TTC
WT	AAA AAT GCC GAA TCT CGT CTG AAT CAT CAT TTG TCA GGA CTC TTT GGC GTA AGT TCC TTG GCT TGG ACA GGG
Δ*psaB*-1	
Δ*psaB*-2	AAA AAT GCC GAA TCT CGT CTG AAT CAT CAT TTG TCA GGA CTC TTT GGC GTA AGT TCC TTG GCT TGG ACA GGG
WT	CAT TTA GTT CAT GTT GCT ATT CCT GCA TCC AGA GGG GAG TAC GTT CGG TGG AAT AAT TTC TTA GAT GTA TTA
Δ*psaB*-1	
Δ*psaB*-2	CAT TTA GTT CAT GTT GCT ATT CCT GCA TCC AGA GGG GAG TAC GTT CGG TGG AAT AAT TTC TTA GAT GTA TTA
WT	CCG CAT CCC CAA GGG TTA GGC CCA CTT TTT ACA GGT CAA TGG AAT CTT TAT GCT CAA AAC CCC GAT TCA AGT
Δ*psaB*-1	
Δ*psaB*-2	CCG CAT CCC CAA GGG TTA GGC CCA CTT TTT ACA GGT CAA TGG AAT CTT TAT GCT CAA AAC CCC GAT TCA AGT
WT	AGT CAT TTA TTT GGT ACC GCC CAA GGG GCG GGA ACT GCC ATT CTA ACT CTT CTC GGG GGA TTC CAT CCA CAA
Δ*psaB*-1	
Δ*psaB*-2	AGT CAT TTA TTT GGT ACC GCC CAA GGG GCG GGA ACT GCC ATT CTA ACT CTT CTC GGG GGA TTC CAT CCA CAA

WT ACG CAA AGT TTA TGG CTG ACT GAT ATT GCC CAT CAC CAT TTA GCT ATT GCA TTT ATT TTT CTC GTT GCT GGT

Δ*psaB*-1

Δ*psaB*-2 ACG CAA AGT TTA TGG CTG ACT GAT ATT GCC CAT CAC CAT TTA GCT ATT GCA TTT ATT TTT CTC GTT GCT GGT

WT CAT ATG TAT AGA ACC AAT TTC GGG ATT GGG CAC AGT ATG AAA GAC CTT TTA GAT GCA CAT ATT CCC CCG GGG

Δ*psaB*-1

Δ*psaB*-2 CAT ATG TAT AGA ACC AAT TTC GGG ATT GGG CAC AGT ATGAAA GAC CTT TTA GAT GCA CAT ATT CCC CCG GGG

WT GGA CGA TTG GGG CGT GGA CAT AAG GGT CTT TAT GAC ACA ATC AAT AAT TCG CTT CAT TTT CAA TTA GGC CTT

Δ*psaB*-1

Δ*psaB*-2 GGA CGA TTG GGG CGT GGC CAT AAG GGC CTT ACG ACA CAA TCA ATA ATT CGC TTC ATT TTC AAT **TAG Stop**

WT GCT CTA GCT TCT TTA GGG GTT ATT ACT TCT TTG GTA GCT CAA CAC ATG TAC TCT TTA CCT GCT TAT GCA TTC

Δ*psaB*-1

Δ*psaB*-2

WT ATA GCA CAA GAC TTT ACT ACT CAA GCT GCA TTA TAT ACC CAC CAC CAA TAT ATC GCA GGA TTC ATC ATG ACA

Δ*psaB*-1

Δ*psaB*-2

WT GGA GCT TTT GCT CAT GGA GCT ATA TTT TTC ATT AGA GAT TAC AAT CCG GAG CAA AAT GAA GAT AAT GTA TTG

Δ*psaB*-1

Δ*psaB*-2

WT GCA AGA ATG TTA GAG CAT AAA GAA GCT ATC ATA TCT CAT TTA AGT TGG GCC AGC CTC TTT CTG GGA TTC CAT

Δ*psaB*-1

Δ*psaB*-2

WT ACC CTG GGA CTT TAT GTT CAT AAT TTT GCT CAA TGG ATA CAA TCC GCT CAT GGT AAA ACT TCA TAT GGG TTC

Δ*psaB*-1

Δ*psaB*-2

WT GAT GTA CTT TTA TCT TCA ACG AGT GGT CCA GCA TTC AAT GCG GGT CGA AGC ATC TGG TTG CCG GGT TGG TTA

Δ*psaB*-1

Δ*psaB*-2

WT AAT GCT GTT AAT GAA AAT AGT AAT TCA TTA TTT TTA ACA ATA GGT CCT GGA GAC TTT TTG GTT CAT CAT GCT

Δ*psaB*-1

Δ*psaB*-2

WT ATT GCT CTT GGT TTA CAT ACA ACT ACA TTG ATC TTA GTA AAA GGT GCT TTA GAT GCA CGT GGT TCC AAG TTA

Δ*psaB*-1

Δ*psaB*-2

WT ATG CCA GAT AAA AAG GAT TTC GGT TAT AGT TTT CCG TGC GAT GGC CCA GGA CGA GGC GGT ACT TGT GAT ATT

Δ*psaB*-1

Δ*psaB*-2

WT TCG GCA TGG GAC GCG TTT TAT TTG GCA GTT TTT TGG ATG TTA AAT ACT ATT GGA TGG GTT ACT TTT TAT TGG
Δ*psaB*-1
Δ*psaB*-2

WT CAT TGG AAG CAC ATC ACA TTA TGG CAG GGT AAC GTT TCA CAG TTT AAT GAA TCT TCC ACT TAT TTG ATG GGC
Δ*psaB*-1
Δ*psaB*-2

WT TGG TTA AGG GAT TAT TTA TGG TTA AAC TCT TCA CAA CTT ATC AAC GGA TAT AAT CCT TTT GGT ATG AAT AGT
Δ*psaB*-1
Δ*psaB*-2

WT TTA TCG GTT TGG GCA TGG ATG TTC TTA TTT GGA CAT CTT GTT TGG GCT ACT GGA TTT ATG TTC TTA ATT TCT
Δ*psaB*-1
Δ*psaB*-2

WT TGG CGT GGA TAT TGG CAG GAA TTG ATT GAA ACT TTA GCA TGG GCT CAT GAA CGC ACA CCT TTG GCC AAT TTG
Δ*psaB*-1
Δ*psaB*-2

WT ATT CGC TGG AGA GAT AAA CCA GTG GCC CTT TCC ATT GTA CAA GCA AGA TTG GTT GGA TTA GCT CAC TTT TCT

Δ*psaB*-1

Δ*psaB*-2

WT GTA GGT TAT ATA TTC ACT TAT GCG GCT TTC TTG ATT GCC TCT ACG TCG GGC AAA TTT GGT **TAA Stop**

Δ*psaB*-1

Δ*psaB*-2

Table S4: Overview of used antibodies

IgG: Immunoglobulin G

**Antibody against**	**Target size (kDa)**	**Working dilution**	**Source, Supplier, additional information**
AtpB	54	1:5000	Agrisera, Vännäs, Sweden (AS05085)
PetB	24	1:5000	Agrisera Vännäs, Sweden (AS184169)
PetA	34	1:2000	Agrisera Vännäs, Sweden (AS06119)
PsaD	20	1:1000	Agrisera, Vännäs, Sweden (AS09461)
PsbA	38 (runs at 25-30)	1:10,000	Agrisera, Vännäs, Sweden (AS10704)
PsbB	56	1:2000	Agrisera, Vännäs, Sweden (AS04038)
PsbC	45	1:3000	Agrisera, Vännäs, Sweden (AS111787)
PsbD	39.5 (runs at 25-30)	1:10,000	Agrisera, Vännäs, Sweden (AS06146), used for all PsbD westerns (except those shown in Fig. S7)
PsbD	39.5 (runs at 25-30)	1:2000	Abcam, Cambridge, UK (ab65392), used for attempts to detect truncated PsbD in Δ3'-*psbD/C* (Fig. S7)
PsbD	39.5 (runs at 25-30)	1:1000	PhytoAB, San Jose, CA, USA (PHY0090A), used for attempts to detect truncated PsbD in Δ3'-*psbD/C* (Fig. S7)
PsbH	8	1:5000	Agrisera, Vännäs, Sweden (AS06157)
Rabbit IgG	n/a	1: 10,000	Bio-Rad Laboratories GmbH (Hercules, CA, USA) (170-6515)

Table S5: Overview of used primers

Name	Sequence 5' to 3'	Description
f-*psbA*	CCTTGACTGTCAACTACAGATTGG	Forward primer for *psbA* PCR probe
r-*psbA*	TACTTGGCGTAGCTTGTTACATGG	Reverse primer for *psbA* PCR probe
f-*atpH*	TTAAACAAAAGGATTCGCA	Forward primer for *atpH* PCR probe
r-*atpH*	ATGAATCCACTGATTTCTG	Reverse primer for *atpH* PCR probe
f-*atpF*	CTTTCATTGTTCCCAACATGC	Forward primer for *atpF* PCR probe
r-*atpF*	GAAATTCAGAAGAACTGCGTG	Reverse primer for *atpF* PCR probe
f-*psaB*	CCCAAGGGGCGGGAACTGC	Forward primer for *psaB* PCR probe
r-*psaB*	CCCAGAAAGAGGCTGGCCC	Reverse primer for *psaB* PCR probe

Supplemental Figures

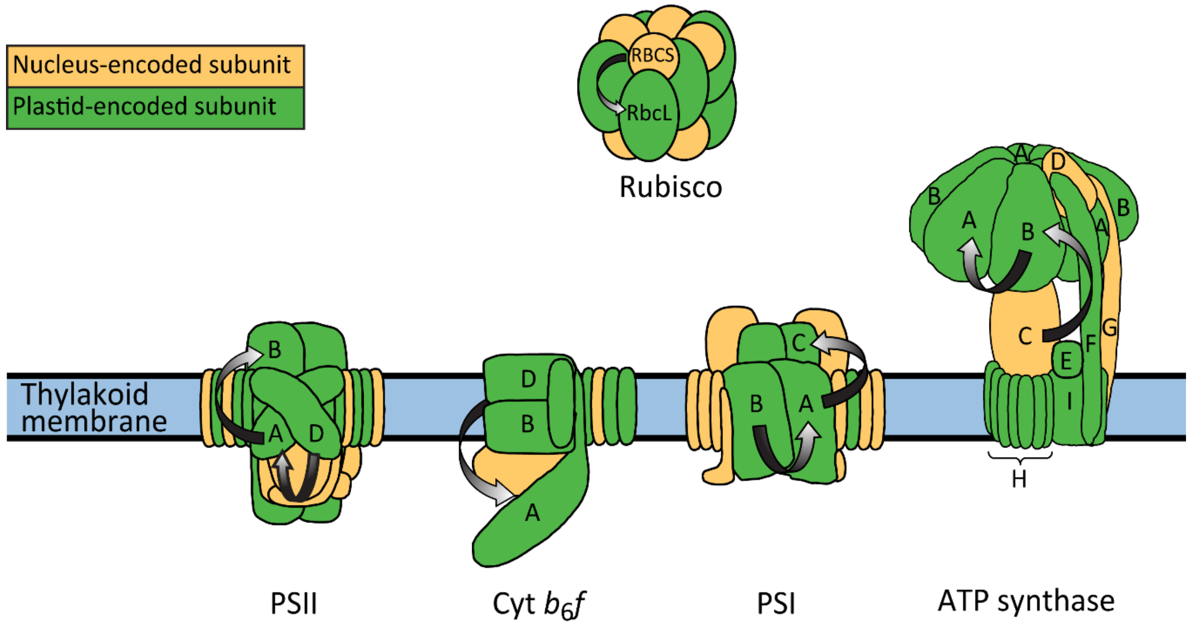

Fig. S1. Summary of the previously known translational feedback regulation during the assembly of photosynthetic protein complexes in Chlamydomonas. Plastid-encoded subunits are shown in green, while nucleus-encoded subunits are displayed in yellow. Black arrows represent CES interactions previously identified in Chlamydomonas[35]. The figure is adapted from Choquet and Wollman[36].

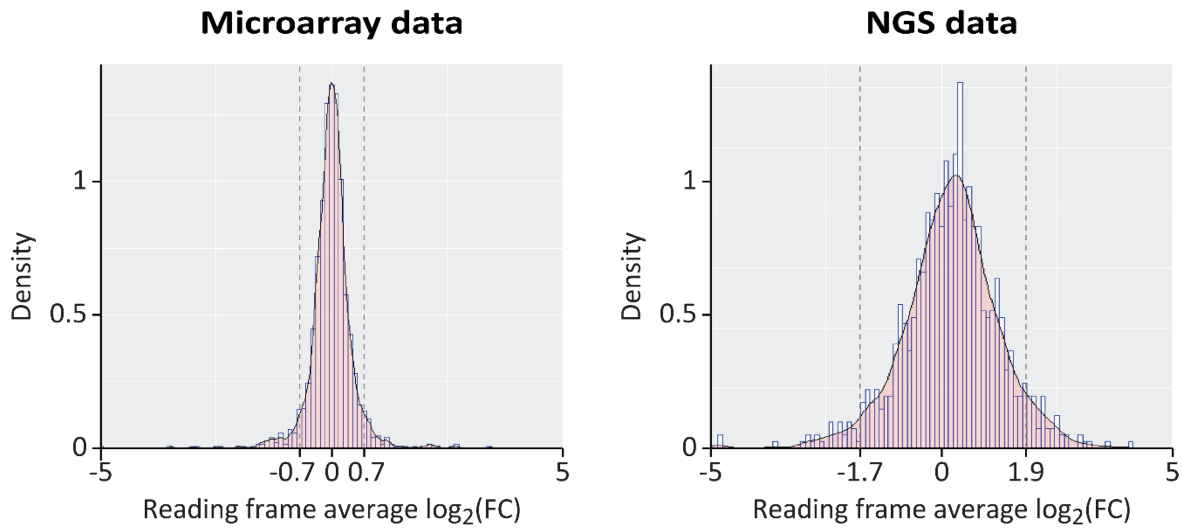

Fig. S2. Fold change distribution of all translatome and transcriptome data. Log_2 fold change of reading frame averages calculated from the combined translation output and RNA abundance data for all chloroplast-encoded reading frames in all mutants analyzed by microarray (left) or NGS (right) were plotted. Data generated with either approach show a normal distribution. Note the rather narrow distribution of the microarray data compared to the broader distribution of the NGS data, which may be attributable to (i) technical differences, including the different sensitivity and dynamic detection range of the methods, and/or (ii) the stronger impairment of knockout mutant alleles analyzed by NGS compared to the milder hypomorphic mutant alleles analyzed by microarrays. According to the 5th percentile of the distributions, a cut-off of twofold and fourfold change was chosen for the microarray and NGS data, respectively, to define chloroplast reading frames whose expression is substantially altered on transcript or translational levels. Vertical dashed lines represent the 5th and 95th percentile values.

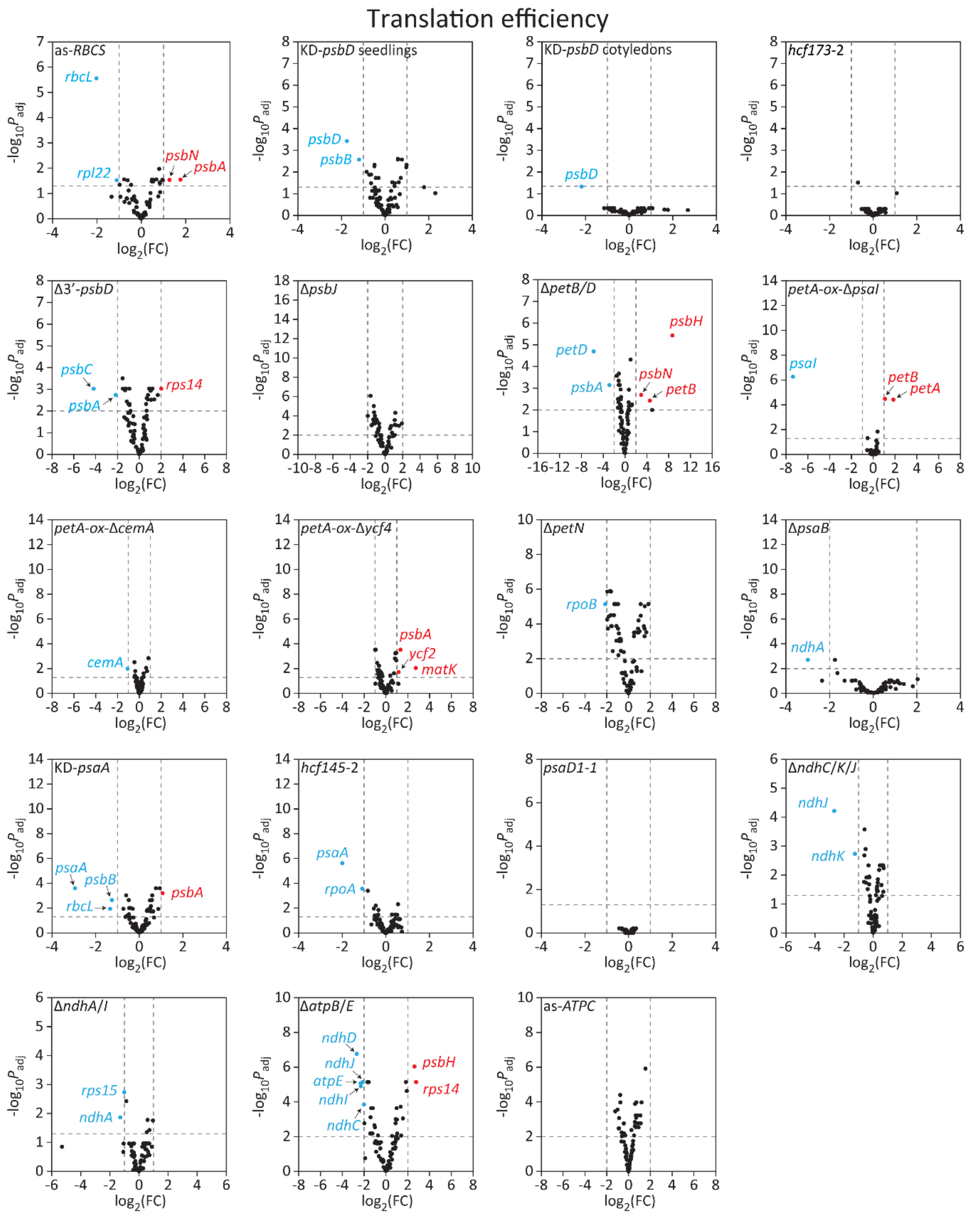

Fig. S3 Comparison of translation efficiencies for chloroplast reading frames in each mutant analyzed in this study. Translation efficiencies were calculated as ratios of translation output to transcript abundance, providing a normalized measure that accounts for changes in transcript levels and enables the distinction between regulation at transcriptional and translational levels. The results are presented as volcano plots, with the mutant name indicated in the upper left corner of each panel. Data from three biological replicates were

summarized, and Log_2-fold changes between mutant and control plants were plotted in volcano plots against the negative common logarithm of the adjusted *P* values (False Discovery Rate (FDR))[37]. Thresholds are the same as in all other data analyses (see Methods): Genes showing at least twofold or fourfold downregulation and upregulation with adjusted *P* values of ≤ 0.05 or ≤ 0.01 for microarray-based or NGS-based data are highlighted in blue and red, respectively. Vertical and horizontal dashed lines indicate the fold-change and adjusted P value cut-off, respectively.

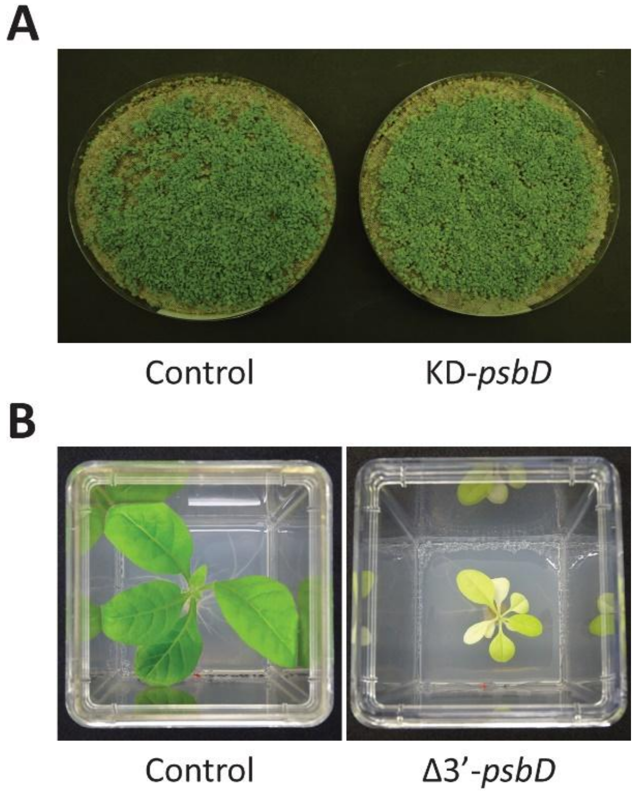

Fig. S4. Phenotypes of *psbD* mutants. (A) KD-*psbD* at the cotyledon stage. (B) Δ3'-*psbD* grown under heterotrophic conditions.

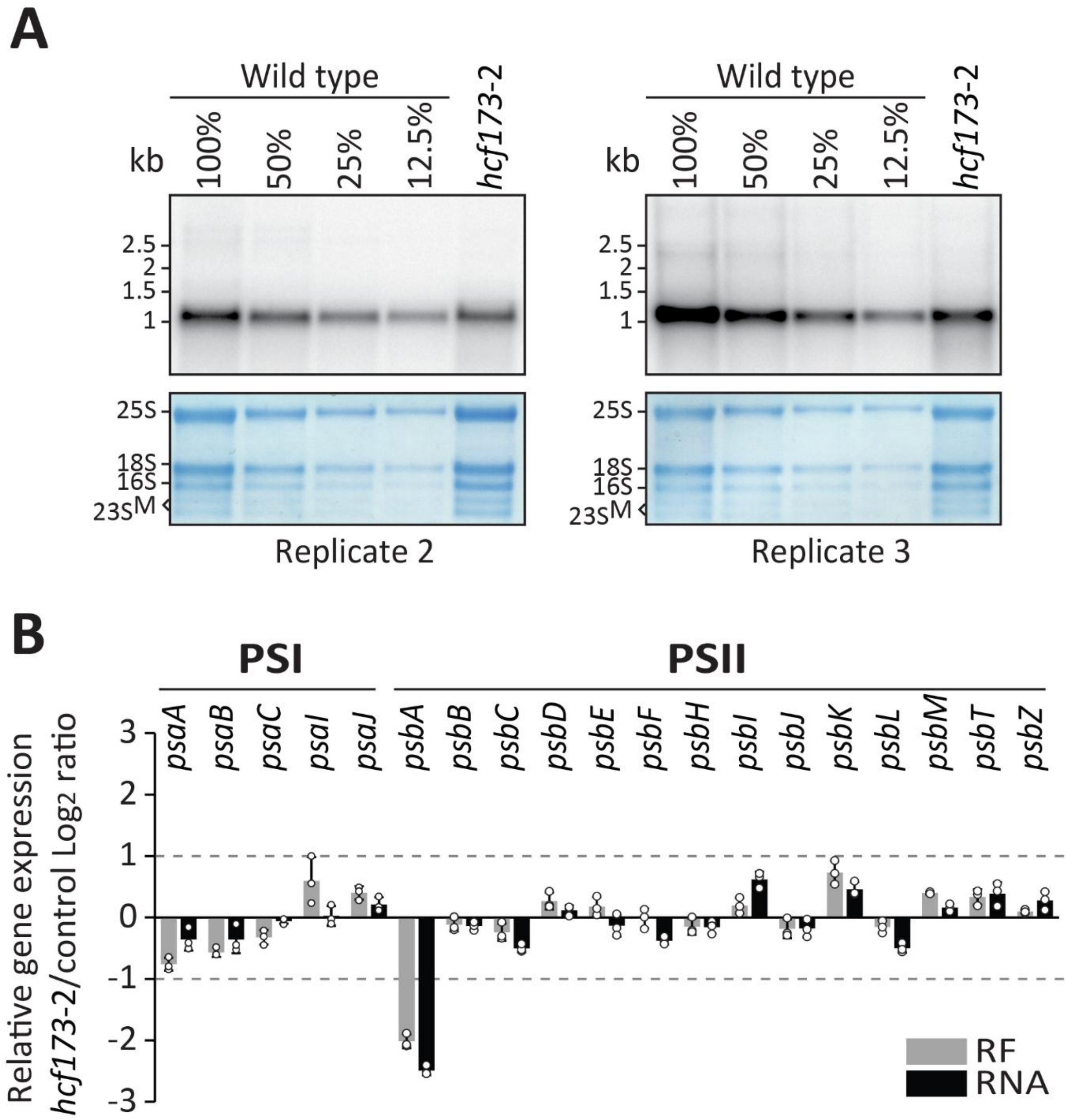

Fig. S5. ***psbA*** **transcript accumulation defect and PSI and PSII gene expression in the** ***hcf173-2*** **mutant.** (A) Biological replicates 2 and 3 of RNA gel blot analysis with a *psbA* probe (replicate 1 is shown in Fig. 1*H*). Other details are as in Fig. 1*H*. (B) Translation output (RF) and RNA accumulation of chloroplast PSI and PSII genes in the Arabidopsis *hcf173* mutant relative to the wild type (Table S1). Error bars represent standard deviations of three biological replicates (data points of replicates are shown as dots).

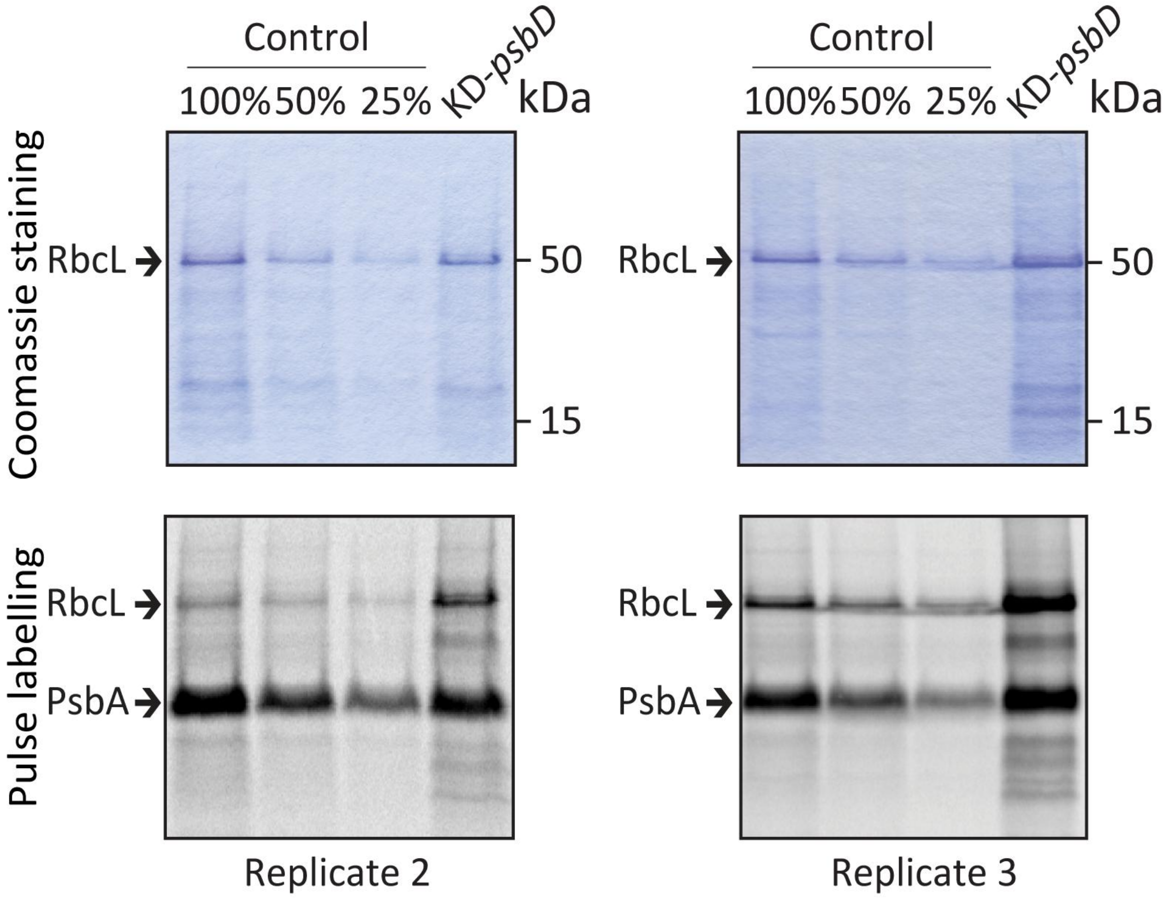

Fig. S6. Pulse labeling showing the reduction of *de novo* synthesized PsbA in KD-*psbD* at the cotyledon stage. Shown are biological replicates 2 and 3 (biological replicate 1 is shown in Fig. 1*D*). Quantification of the three biological replicates is shown in Fig. 1*E*. Note that, different from replicate 1 shown in Fig. 1*D*, for replicates 2 and 3, the lane with the protein that derives from KD-*psbD* contains more labeled protein, which is visible by the strong RbcL bands and the increased background signal. To exclude effects of unequal loading and labeling efficiencies from the analysis, for all replicates, the PsbA signal intensity was quantified using Image Lab software (Bio-Rad) and normalized to the background signal excluding the RbcL band.

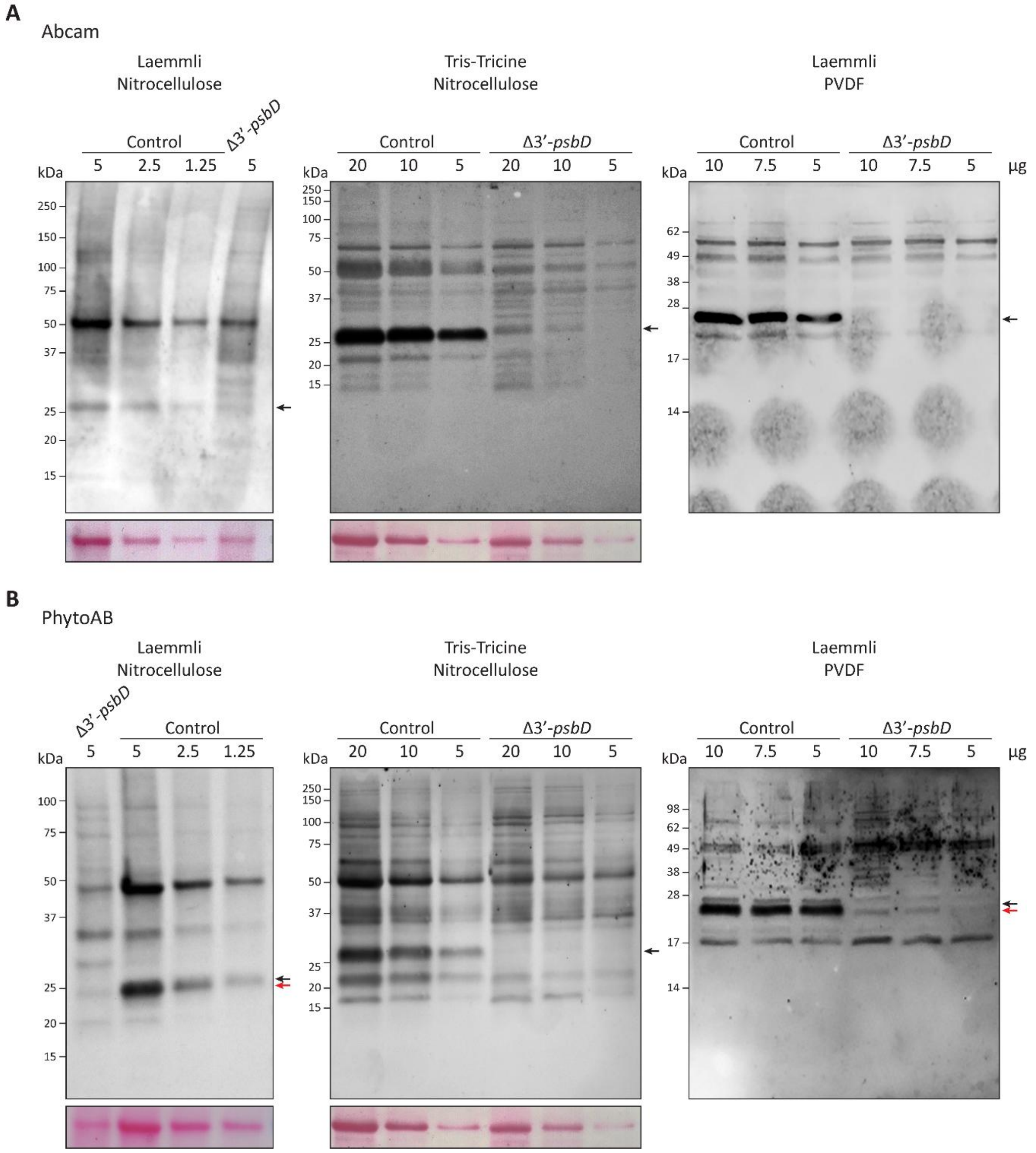

Fig. S7. Attempts to detect a C-terminally truncated PsbD protein in the Δ*3'*-*psbD* mutant. (A) Detection of PsbD using an Abcam antibody raised against a synthetic peptide corresponding to amino acids 230-240 (sequence: ANTFRAFNPTQ) of PsbD. Three different western blotting techniques were applied to evaluate the detection of the truncated PsbD (for details see Methods): Laemmli SDS-polyacrylamide gel electrophoresis (PAGE) with transfer to nitrocellulose (left panel), Tris-tricine SDS-PAGE with transfer to nitrocellulose (middle panel), and Laemmli SDS-PAGE with transfer to PVDF membrane (right panel, note that PVDF is not stainable by Ponceau S). Molecular weight markers are labeled on the left. The black arrow indicates the full length PsbD (confirmed by the absence of the signal in the

Δ3'-psbD mutant). (B) Detection of PsbD using a PhytoAB antibody targeting 15 amino acids from the N-terminus of the protein. Similar blotting approaches were applied as in *A*. The black arrow indicates the full length PsbD (confirmed by the absence of the signal in the *Δ3'-psbD* mutant). The red arrow labels a band that is specifically detected in the *Δ3'-psbD* mutant with the PsbD antibody from PhytoAB and may represent a truncated version of PsbD. By visual inspection this additional band seems to be too close to the mature PsbD (visible in the control line) to represent the substantially truncated version of PsbD in the *Δ3'-psbD* mutant (104 C-terminal amino acids of the 353 amino acids of PsbD are deleted in Δ*3'psbD*). On the other hand, the truncation deletes only one of the five transmembrane domains (TMDs) of PsbD. Since TMDs are very hydrophobic, they strongly influence the running behavior of proteins in gels (making the 39.5 kDa proteins PsbA and PsbD running at 25-30 kDa). One could speculate that deleting only one of these five TMDs at the C-terminus may not strongly change the running behavior of the truncated PsbD compared to full-length PsbD.

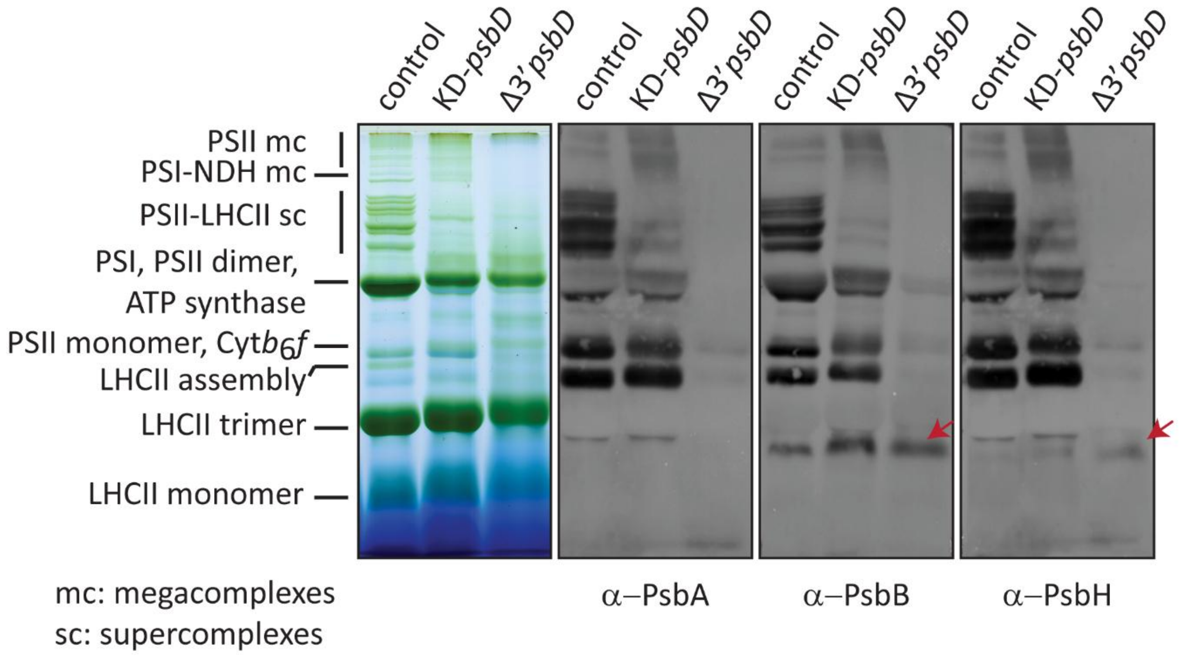

Fig. S8. PSII complex assembly defects in KD-*psbD* and Δ3'-*psbD* mutants. Thylakoid membranes were isolated from the control and the KD-*psbD* as well as the Δ3'-*psbD* mutants (Table S1), all of which were grown heterotrophically at low light intensity (Table S2). Samples of 8 µg thylakoid protein were solubilized in β-DDM (n-dodecyl β-D-maltoside) and separated by electrophoresis in a native 5-12.5% gradient polyacrylamide gel. Major resolved photosynthetic complexes are labeled on the left side. Immunoblot analysis of the first dimension with antibodies against PSII subunits confirmed the expected severe defect in PSII assembly in both mutants. In the Δ3'-*psbD* mutant, PsbA cannot be detected in any of the major PSII-containing complexes. However, in the lower molecular weight range, PsbB and PsbH signals are visible in the Δ3'-*psbD* mutant (marked with a red arrow), which likely correspond to a PSII assembly intermediate that contains these two (and possibly other) proteins.

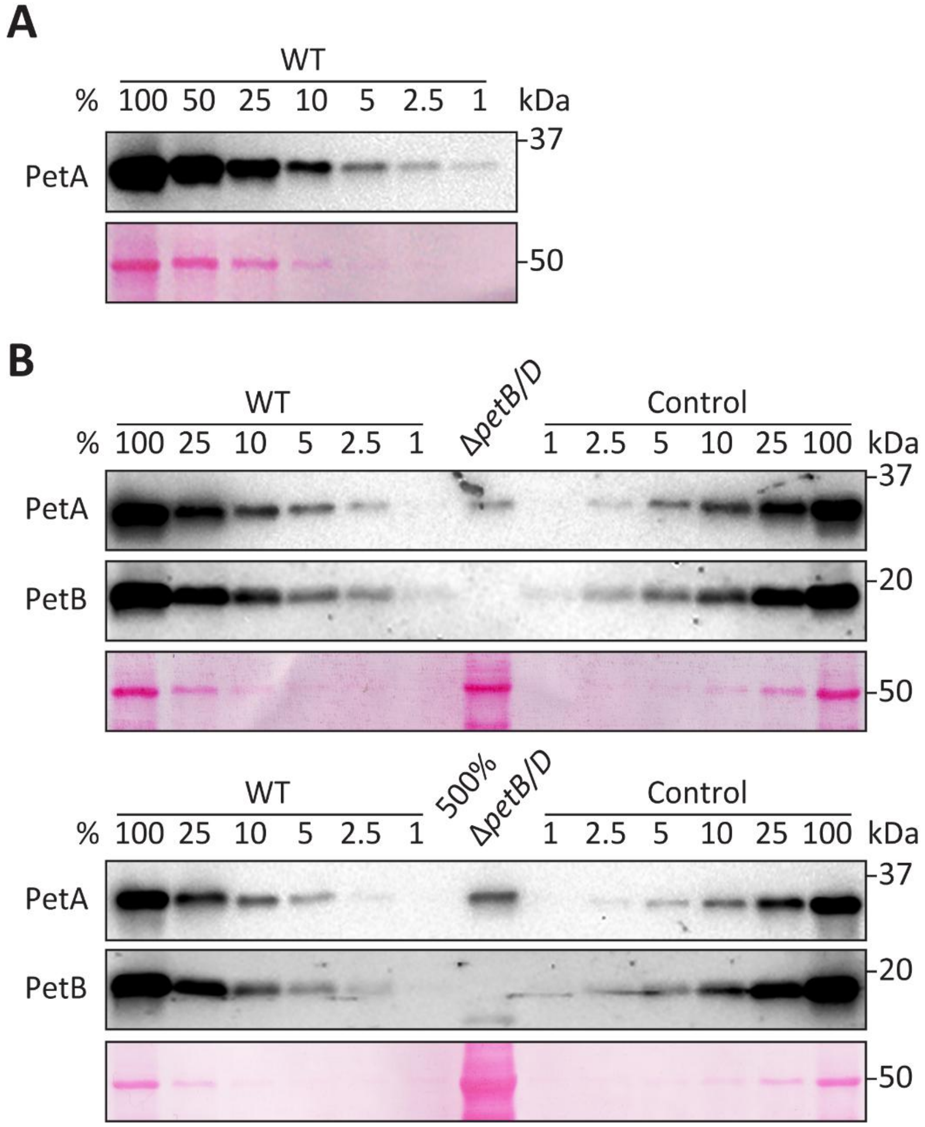

Fig. S9. Western blot analysis to determine PetA accumulation in the Δ*petB*/*D* mutant. (A) Dilution series to assess the PetA antibody sensitivity. The 100% sample represents 5 µg of total protein from tobacco wild-type plants that were separated by SDS-PAGE and probed with a PetA antibody. Ponceau-S-staining confirms the serial dilution tested. A faint signal was still detectable at 1% dilution, confirming the high sensitivity of the PetA antibody. (B) Biological replicate of the PetA and PetB immunoblot analysis shown in Fig. 3*F*. Other details are as in Fig. 3*F*.

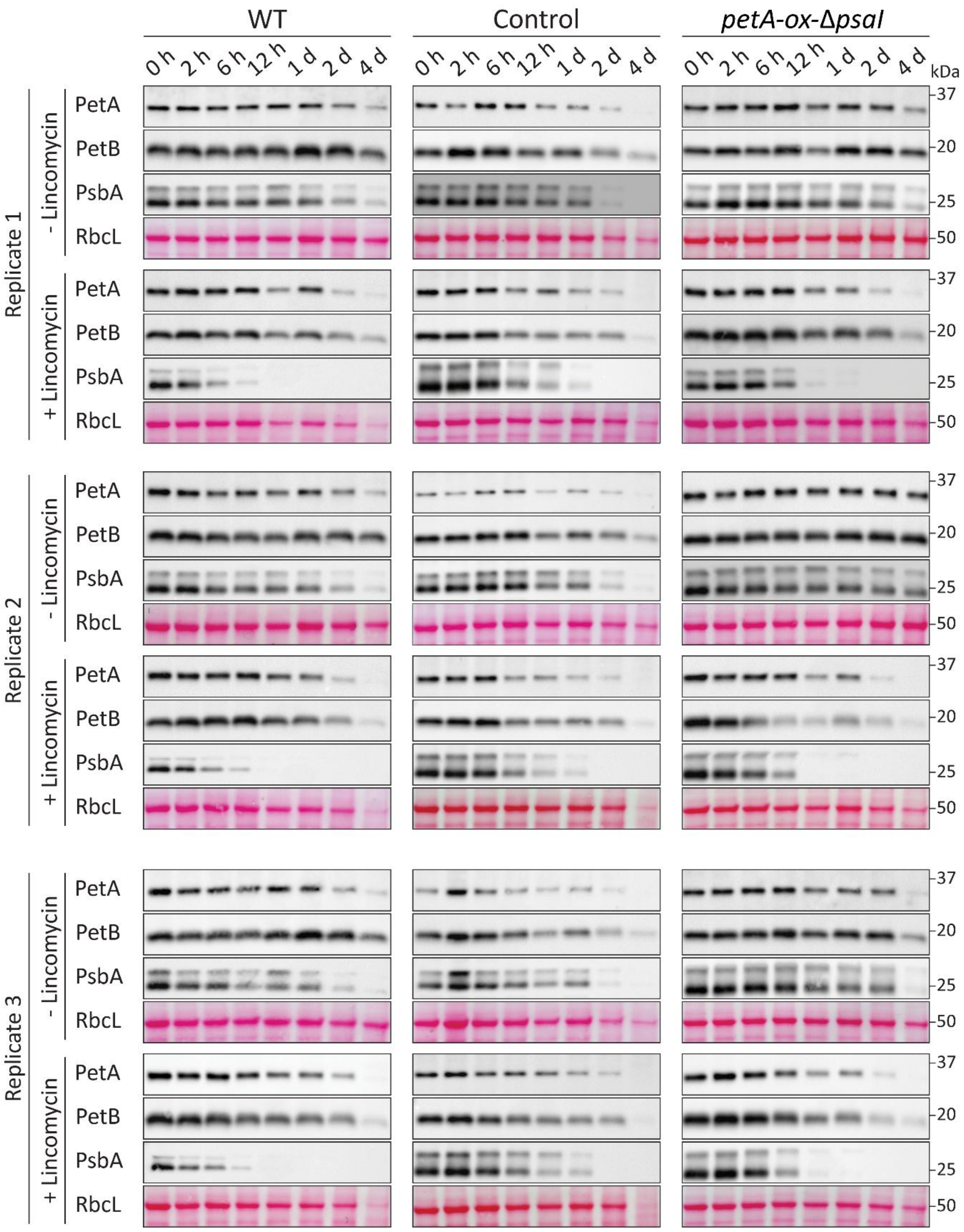

Fig. S10. Comparative determination of PetA and PetB turnover in tobacco. Leaf discs from wild type, control plants and the *petA*-ox-Δ*psaI* mutant were vacuum-infiltrated with lincomycin and incubated overnight before collecting the tissue for protein extraction at selected time points. Control sets of leaf discs from all genotypes were similarly treated with buffers excluding lincomycin. The extracted proteins were separated by SDS-PAGE. Loading was based on equal total protein amount. Subsequently, the turnover of PetA, PetB and PsbA

was visualized by immunoblot analysis. Ponceau-S-staining confirms the equal loading and estimates the accumulation of RbcL. Analyses were performed in three biological replicates (all biological replicates are shown).

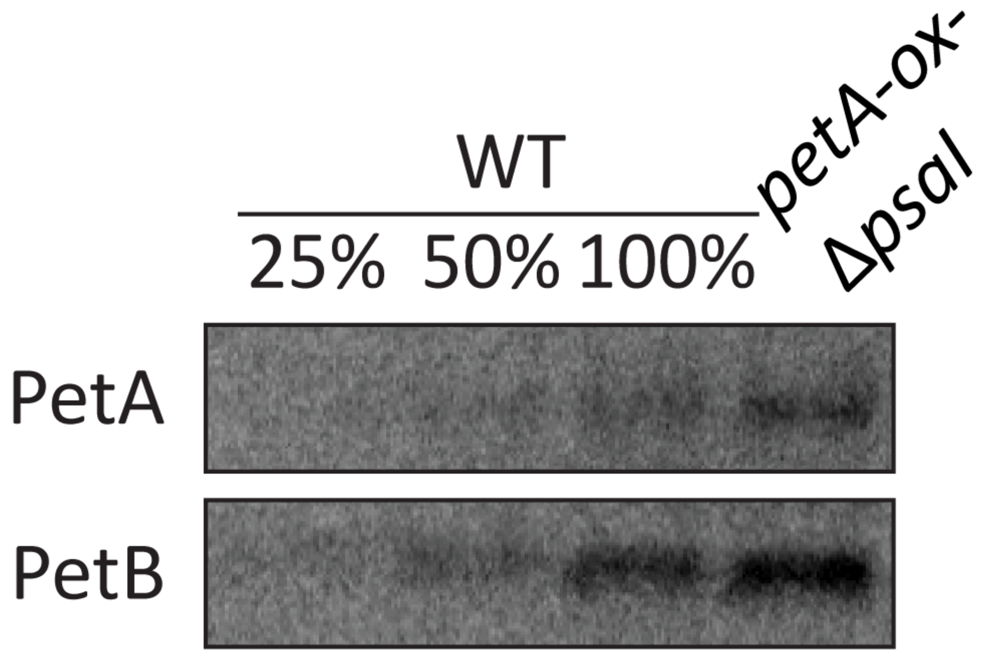

Fig. S11. Pulse-IP of PetA and PetB in the *petA*-ox-Δ*psal* mutant. After radiolabeling of *de novo*-synthesized proteins, immunoprecipitation with antibodies against PetA and PetB was performed with equal amounts of labeled proteins (see Material and Methods). Proteins were then separated by SDS-PAGE. The experiment was done in two biological replicates and the other replicate is shown in Fig. 4*E*.

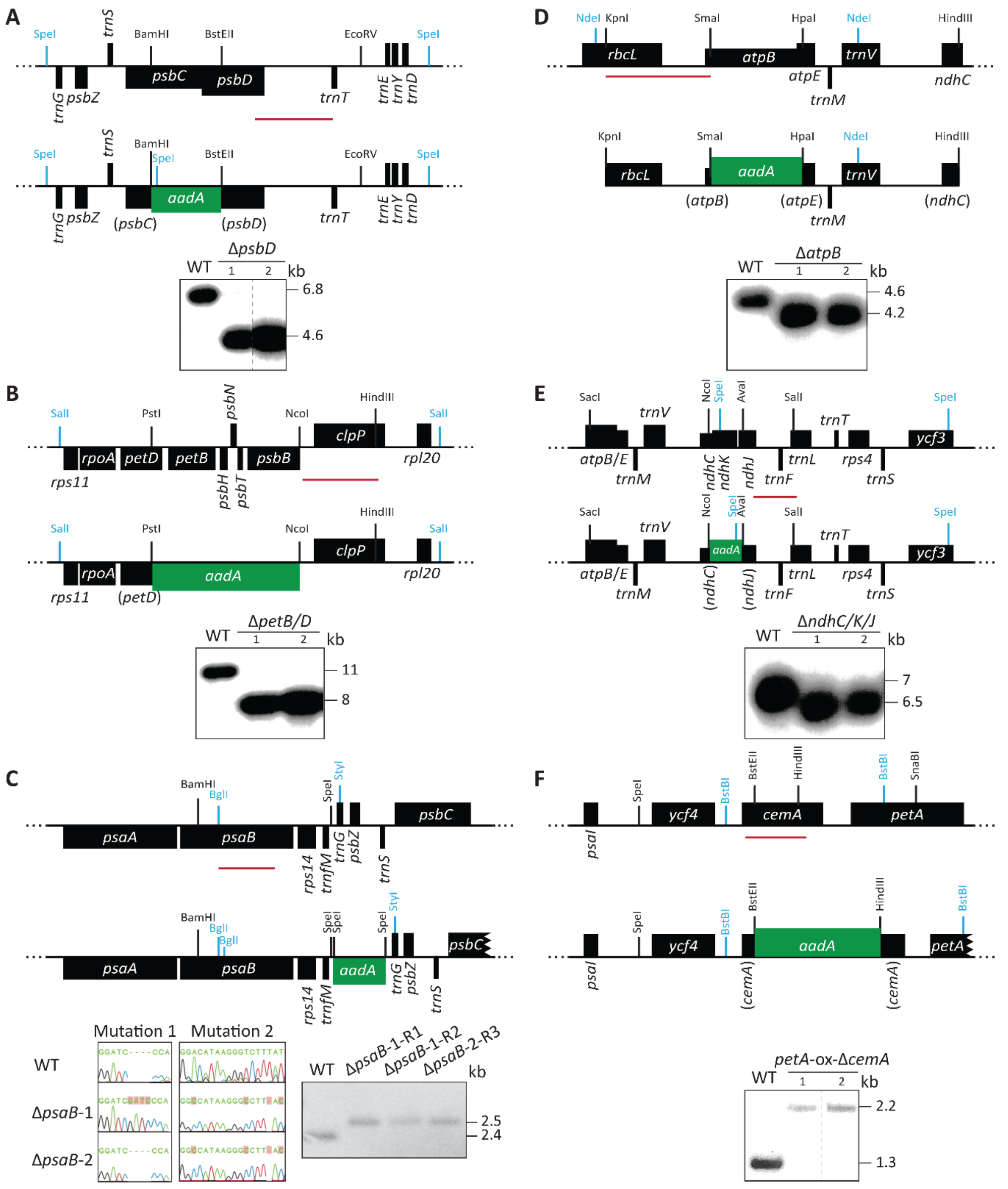

Fig. S12. Cloning strategies of chloroplast transformation vectors and restriction fragment length polymorphism (RFLP) analyses of mutants that were not previously described. Details of the cloning strategies and the RFLP analyses are provided in the Methods. 3 µg genomic DNA extracted from several independently generated transplastomic lines (i.e., transformation events) was fragmented with restriction enzymes whose recognition sites are labeled in blue, separated by agarose gel electrophoresis, transferred to nylon membranes, and hybridized to radiolabeled probes whose genomic position is

represented by red bars. Bands of expected sizes are indicated. For some blots, irrelevant lanes were removed (indicated by black dotted vertical lines). (A) Δ3'-*psbD*, (B) Δ*petB/D*, (C) Δ*psaB*-1 and Δ*psaB*-2 (note that the point mutation inserted in Δ*psaB*-2 creates an additional BglI restriction site) (D) Δ*atpB*/*E*, (E) Δ*ndhC*/*K*/*J*, (F) *petA*-ox-Δ*cemA*. (C) Chromatograms from Sanger sequencing of the two independent transplastomic alleles, Δ*psaB*-1 and Δ*psaB*-2, are shown to illustrate the homoplasmy of the introduced point mutations.

References

1 Ting, M. K. Y. *et al.* Optimization of ribosome profiling in plants including structural analysis of rRNA fragments. *Plant Methods* **20**, 143, doi:10.1186/s13007-024-01267-3 (2024).

2 Trösch, R. *et al.* Commonalities and differences of chloroplast translation in a green alga and land plants. *Nat Plants* **4**, 564-575, doi:10.1038/s41477-018-0211-0 (2018).

3 Khrebtukova, I. & Spreitzer, R. J. Elimination of the *Chlamydomonas* gene family that encodes the small subunit of ribulose-1,5-bisphosphate carboxylase/oxygenase. *Proc Natl Acad Sci USA* **93**, 13689-13693 (1996).

4 Wostrikoff, K. & Stern, D. Rubisco large-subunit translation is autoregulated in response to its assembly state in tobacco chloroplasts. *Proc Natl Acad Sci USA* **104**, 6466-6471, doi:10.1073/pnas.0610586104 (2007).

5 Wietrzynski, W., Traverso, E., Wollman, F. A. & Wostrikoff, K. The state of oligomerization of Rubisco controls the rate of synthesis of the Rubisco large subunit in *Chlamydomonas reinhardtii*. *Plant Cell* **33**, 1706-1727, doi:10.1093/plcell/koab061 (2021).

6 Rodermel, S. R., Abbott, M. S. & Bogorad, L. Nuclear-organelle interactions: nuclear antisense gene inhibits ribulose bisphosphate carboxylase enzyme levels in transformed tobacco plants. *Cell* **55**, 673-681 (1988).

7 Shikanai, T. Chloroplast NDH: A different enzyme with a structure similar to that of respiratory NADH dehydrogenase. *Biochim Biophys Acta* **1857**, 1015-1022, doi:10.1016/j.bbabio.2015.10.013 (2016).

8 Ma, M., Liu, Y., Bai, C. & Yong, J. W. H. The significance of chloroplast NAD(P)H dehydrogenase complex and its dependent cyclic electron transport in photosynthesis. *Front Plant Sci* **12**, 661863, doi:10.3389/fpls.2021.661863 (2021).

9 Introini, B., Hahn, A. & Kuhlbrandt, W. Cryo-EM structure of the NDH-PSI-LHCI supercomplex from *Spinacia oleracea*. *Nat Struct Mol Biol*, doi:10.1038/s41594-024-01478-1 (2025).

10 Su, X. *et al.* Supramolecular assembly of chloroplast NADH dehydrogenase-like complex with photosystem I from *Arabidopsis thaliana*. *Mol Plant* **15**, 454-467, doi:10.1016/j.molp.2022.01.020 (2022).

11 Ifuku, K., Endo, T., Shikanai, T. & Aro, E. M. Structure of the chloroplast NADH dehydrogenase-like complex: nomenclature for nuclear-encoded subunits. *Plant Cell Physiol* **52**, 1560-1568, doi:10.1093/pcp/pcr098 (2011).

12 Peng, L., Fukao, Y., Fujiwara, M. & Shikanai, T. Multistep assembly of chloroplast NADH dehydrogenase-like subcomplex A requires several nucleus-encoded proteins, including CRR41 and CRR42, in *Arabidopsis*. *Plant Cell* **24**, 202-214, doi:10.1105/tpc.111.090597 (2012).

13 Hager, M. Anwendung reverser Genetik zur Funktionsanalyse plastidenkodierter Gene. *Albert-Ludwigs-Universität Freiburg* (2002).

14 Ghandour, R. *et al.* Transgene insertion into the plastid genome alters expression of adjacent native chloroplast genes at the transcriptional and translational levels. *Plant Biotechnol J* **21**, 711-725, doi:10.1111/pbi.13985 (2023).

15 Kofer, W., Koop, H. U., Wanner, G. & Steinmüller, K. Mutagenesis of the genes encoding subunits A, C, H, I, J and K of the plastid NAD(P)H-plastoquinone-oxidoreductase in tobacco by polyethylene glycol-mediated plastome transformation. *Mol Gen Genet* **258**, 166-173, doi:10.1007/s004380050719 (1998).

16 Meteignier, L. V. *et al.* The Arabidopsis mTERF-repeat MDA1 protein plays a dual function in transcription and stabilization of specific chloroplast transcripts within the *psbE* and *ndhH* operons. *New Phytol* **227**, 1376-1391, doi:10.1111/nph.16625 (2020).

17 Zoschke, R., Watkins, K. P., Miranda, R. G. & Barkan, A. The PPR-SMR protein PPR53 enhances the stability and translation of specific chloroplast RNAs in maize. *Plant J* **85**, 594-606, doi:10.1111/tpj.13093 (2016).

18 Castandet, B., Germain, A., Hotto, A. M. & Stern, D. B. Systematic sequencing of chloroplast transcript termini from *Arabidopsis thaliana* reveals >200 transcription initiation sites and the extensive imprints of RNA-binding proteins and secondary structures. *Nucleic Acids Res* **47**, 11889-11905, doi:10.1093/nar/gkz1059 (2019).

19 Maria del Campo, E., Sabater, B. & Martin, M. Characterization of the 5'- and 3'-ends of mRNAs of *ndhH*, *ndhA* and *ndhI* genes of the plastid ndhH-D operon. *Biochimie* **88**, 347-357, doi:10.1016/j.biochi.2005.09.005 (2006).

20 Del Campo, E. M., Sabater, B. & Martin, M. Post-transcriptional control of chloroplast gene expression. Accumulation of stable *psaC* mRNA is due to downstream RNA cleavages in the *ndhD* gene. *J Biol Chem* **277**, 36457-36464, doi:10.1074/jbc.M204500200 (2002).

21 Maliga, P., Sz-Breznovits, A. & Marton, L. Streptomycin-resistant plants from callus culture of haploid tobacco. *Nat New Biol* **244**, 29-30, doi:10.1038/newbio244029a0 (1973).

22 Fu, H. Y. *et al.* The availability of neither D2 nor CP43 limits the biogenesis of photosystem II in tobacco. *Plant Physiol* **185**, 1111-1130, doi:10.1093/plphys/kiaa052 (2021).

23 Bock, R., Kössel, H. & Maliga, P. Introduction of a heterologous editing site into the tobacco plastid genome: the lack of RNA editing leads to a mutant phenotype. *EMBO J* **13**, 4623-4628 (1994).

24 Schult, K. *et al.* The nuclear-encoded factor HCF173 is involved in the initiation of translation of the *psbA* mRNA in *Arabidopsis thaliana*. *Plant Cell* **19**, 1329-1346, doi:10.1105/tpc.106.042895 (2007).

25 Hager, M., Hermann, M., Biehler, K., Krieger-Liszkay, A. & Bock, R. Lack of the small plastid-encoded PsbJ polypeptide results in a defective water-splitting apparatus of photosystem II, reduced photosystem I levels, and hypersensitivity to light. *J Biol Chem* **277**, 14031-14039, doi:10.1074/jbc.M112053200 (2002).

26 Schöttler, M. A. *et al.* The plastid-encoded PsaI subunit stabilizes photosystem I during leaf senescence in tobacco. *J Exp Bot* **68**, 1137-1155, doi:10.1093/jxb/erx009 (2017).

27 Brück, D. Molekulare Analyse von Genfunktionen im Plastidengenom der Tabakpflanze. *Westfälische Wilhelms-Universität Münster* (2003).

28 Krech, K. *et al.* The plastid genome-encoded Ycf4 protein functions as a nonessential assembly factor for photosystem I in higher plants. *Plant Physiol* **159**, 579-591, doi:10.1104/pp.112.196642 (2012).

29 Bock, R. & Maliga, P. In vivo testing of a tobacco plastid DNA segment for guide RNA function in psbL editing. *Mol Gen Genet* **247**, 439-443 (1995).

30 Ruf, S., Hermann, M., Berger, I. J., Carrer, H. & Bock, R. Stable genetic transformation of tomato plastids and expression of a foreign protein in fruit. *Nat Biotechnol* **19**, 870-875, doi:10.1038/nbt0901-870 (2001).

31 Manavski, N. *et al.* HIGH CHLOROPHYLL FLUORESCENCE145 binds to and stabilizes the *psaA* 5' UTR via a newly defined repeat motif in embryophyta. *Plant Cell* **27**, 2600-2615, doi:10.1105/tpc.15.00234 (2015).

32 Lezhneva, L. & Meurer, J. The nuclear factor HCF145 affects chloroplast *psaA-psaB-rps14* transcript abundance in *Arabidopsis thaliana*. *Plant J* **38**, 740-753, doi:10.1111/j.1365-313X.2004.02081.x (2004).

33 Ihnatowicz, A. *et al.* Mutants for photosystem I subunit D of *Arabidopsis thaliana*: effects on photosynthesis, photosystem I stability and expression of nuclear genes for chloroplast functions. *Plant J* **37**, 839-852, doi:10.1111/j.1365-313x.2004.02011.x (2004).

34 Rott, M. *et al.* ATP synthase repression in tobacco restricts photosynthetic electron transport, CO_2 assimilation, and plant growth by overacidification of the thylakoid lumen. *Plant Cell* **23**, 304-321, doi:10.1105/tpc.110.079111 (2011).

35 Choquet, Y. & Wollman, F.-A. in *The Chlamydomonas Sourcebook (Third Edition)* (eds Arthur R. Grossman & Francis-André Wollman) 615-646 (Academic Press, 2023).

36 Choquet, Y. & Wollman, F. A. in *Chlamydomonas Source Book* (eds E. Harris, D.B. Stern, & G. Whitman) 1027–1064 (Academic Press/Elsevier, 2009).

37 Benjamini, Y. & Hochberg, Y. Controlling the false discovery rate: A practical and powerful approach to multiple testing. *Journal of the Royal Statistical Society: Series B (Methodological)* **57**, 289-300, doi:10.1111/j.2517-6161.1995.tb02031.x (1995).
