# Supplementary material for: Assembly-dependent translational feedback regulation of photosynthetic proteins in land plants
Source: Nat Plants. 2025 Aug 18;11(9):1920–38. doi: 10.1038/s41477-025-02074-x (PMC12449265; doi:10.1038/s41477-025-02074-x)
Supplement: Supplementary file 2 — Reporting Summary [file 41477_2025_2074_MOESM2_ESM.pdf]

Corresponding author(s): Reimo Zoschke
Last updated by author(s): Apr 16, 2025

Reporting Summary

Nature Portfolio wishes to improve the reproducibility of the work that we publish. This form provides structure for consistency and transparency in reporting. For further information on Nature Portfolio policies, see our Editorial Policies and the Editorial Policy Checklist.

Statistics

For all statistical analyses, confirm that the following items are present in the figure legend, table legend, main text, or Methods section.

n/a	Confirmed	
☐	☒	The exact sample size (*n*) for each experimental group/condition, given as a discrete number and unit of measurement
☐	☒	A statement on whether measurements were taken from distinct samples or whether the same sample was measured repeatedly
☐	☒	The statistical test(s) used AND whether they are one- or two-sided<br>*Only common tests should be described solely by name; describe more complex techniques in the Methods section.*
☐	☒	A description of all covariates tested
☐	☒	A description of any assumptions or corrections, such as tests of normality and adjustment for multiple comparisons
☐	☒	A full description of the statistical parameters including central tendency (e.g. means) or other basic estimates (e.g. regression coefficient) AND variation (e.g. standard deviation) or associated estimates of uncertainty (e.g. confidence intervals)
☐	☒	For null hypothesis testing, the test statistic (e.g. *F*, *t*, *r*) with confidence intervals, effect sizes, degrees of freedom and *P* value noted<br>*Give P values as exact values whenever suitable.*
☐	☒	For Bayesian analysis, information on the choice of priors and Markov chain Monte Carlo settings
☒	☐	For hierarchical and complex designs, identification of the appropriate level for tests and full reporting of outcomes
☒	☐	Estimates of effect sizes (e.g. Cohen's *d*, Pearson's *r*), indicating how they were calculated

Our web collection on statistics for biologists contains articles on many of the points above.

Software and code

Policy information about availability of computer code

Data collection	NGS data were collected from Illumina NovaSeq 6000 with BCL Converter 2.20.0.422. Microarray data were collected with GenePix Pro 7.2.29.002.
Data analysis	NGS data: For NGS data analysis STAR aligner v2.7.1a, samtools v1.11, bedtools v2.30.0, featureCounts v2.0.1 were used with settings described in the Methods.<br>Microarray data: raw data extraction was done with GenePix Pro 7.2.29.002, all further data analysis was done with MS Excel version 2108. Statistical analysis was done in R v3.5.3 with settings described in the Methods section.

For manuscripts utilizing custom algorithms or software that are central to the research but not yet described in published literature, software must be made available to editors and reviewers. We strongly encourage code deposition in a community repository (e.g. GitHub). See the Nature Portfolio guidelines for submitting code & software for further information.

Data

Policy information about availability of data

All manuscripts must include a data availability statement. This statement should provide the following information, where applicable:
- Accession codes, unique identifiers, or web links for publicly available datasets
- A description of any restrictions on data availability
- For clinical datasets or third party data, please ensure that the statement adheres to our policy

NGS data are available under the GEO accession GSE286011. https://www.ncbi.nlm.nih.gov/geo/

Research involving human participants, their data, or biological material

Policy information about studies with human participants or human data. See also policy information about sex, gender (identity/presentation), and sexual orientation and race, ethnicity and racism.

Reporting on sex and gender	n/a
Reporting on race, ethnicity, or other socially relevant groupings	n/a
Population characteristics	n/a
Recruitment	n/a
Ethics oversight	n/a

Note that full information on the approval of the study protocol must also be provided in the manuscript.

Field-specific reporting

Please select the one below that is the best fit for your research. If you are not sure, read the appropriate sections before making your selection.

☒ Life sciences ☐ Behavioural & social sciences ☐ Ecological, evolutionary & environmental sciences

For a reference copy of the document with all sections, see nature.com/documents/nr-reporting-summary-flat.pdf

Life sciences study design

All studies must disclose on these points even when the disclosure is negative.

Sample size	No sample size calculation was performed. For each analysis three independent biological replicates were used to enable statistical analyses. Each biological replicate was including two to three individual plants. Altogether, 6 to 9 individual plants were analyzed in 3 biological replicates, which is above the standards for sample size in the field of molecular plant biology.
Data exclusions	No data were excluded from the analysis.
Replication	For each analysis three independent biological replicates were used to enable statistical analyses. All attempts at replication were successful.
Randomization	For the different developmental stages and the various growth conditions applied, all analyzed genotypes (i.e., mutants and control lines) were randomly distributed in the respective growth chamber. The same number of compared mutant and control plants were simultaneously grown under the same condition.
Blinding	Data collection and analysis was not blinded because no subjective parameters were measured. Hence, the data analysis would not benefit from blinding.

Reporting for specific materials, systems and methods

We require information from authors about some types of materials, experimental systems and methods used in many studies. Here, indicate whether each material, system or method listed is relevant to your study. If you are not sure if a list item applies to your research, read the appropriate section before selecting a response.

Materials & experimental systems

n/a	Involved in the study	
☐	☒	Antibodies
☒	☐	Eukaryotic cell lines
☒	☐	Palaeontology and archaeology
☒	☐	Animals and other organisms
☒	☐	Clinical data
☒	☐	Dual use research of concern
☐	☒	Plants

Methods

n/a	Involved in the study	
☒	☐	ChIP-seq
☒	☐	Flow cytometry
☒	☐	MRI-based neuroimaging

Antibodies

Antibodies used

α-AtpB (Agrisera, AS05085), α-PetA (Agrisera, AS06119), α-PetB (Agrisera, AS184169), α-PsaD (Agrisera, AS09461), α-PsbA (Agrisera, AS10704), α-PsbB (Agrisera, AS04038), α-PsbC (Agrisera, AS111787), α-PsbD (Agrisera, AS06146), α-PsbD (Abcam, ab65392), α-PsbD (PhytoAB, PHY0090A), α-PsbH (Agrisera, AS06157), Rabbit IgG (Biorad, 170-6515).

Validation

All antibodies are distributed by commercial suppliers. The validation is available from the supplier: α-AtpB (https://www.agrisera.com/en/artiklar/atpb-beta-subunits-of-atp-synthase-global-antibody.html), α-PetA (https://www.agrisera.com/en/artiklar/cytochrome-f-cyt-f-protein-peta-of-thylakoid-cytb6_f-complex.html), α-PetB (https://www.agrisera.com/en/artiklar/cyt-b6-petb-thylakoid-membrane-cytochrome-b6-protein-n-terminal.html), α-PsaD (https://www.agrisera.com/en/artiklar/psad-psi-d-subunit-of-photosystem-i-2.html), α-PsbA (https://www.agrisera.com/en/artiklar/psba-d1-protein-of-psii-de-loop.html), α-PsbB (https://www.agrisera.com/en/artiklar/psbb-cp47-global-antibody.html), α-PsbC (https://www.agrisera.com/en/artiklar/psbc-cp43-protein-of-psii-2.html), α-PsbD (https://www.agrisera.com/en/artiklar/psbd-d2-global-antibody.html), α-PsbD (https://www.abcam.com/en-us/products/unavailable/psbd-antibody-ab65392), α-PsbD (https://www.phytoab.com/psbd%20antibody), α-PsbH (https://www.agrisera.com/en/artiklar/psbh-h-protein.html).

Dual use research of concern

Policy information about dual use research of concern

Hazards

Could the accidental, deliberate or reckless misuse of agents or technologies generated in the work, or the application of information presented in the manuscript, pose a threat to:

No	Yes	
☒	☐	Public health
☒	☐	National security
☒	☐	Crops and/or livestock
☒	☐	Ecosystems
☒	☐	Any other significant area

Experiments of concern

Does the work involve any of these experiments of concern:

No	Yes	
☒	☐	Demonstrate how to render a vaccine ineffective
☒	☐	Confer resistance to therapeutically useful antibiotics or antiviral agents
☒	☐	Enhance the virulence of a pathogen or render a nonpathogen virulent
☒	☐	Increase transmissibility of a pathogen
☒	☐	Alter the host range of a pathogen
☒	☐	Enable evasion of diagnostic/detection modalities
☒	☐	Enable the weaponization of a biological agent or toxin
☒	☐	Any other potentially harmful combination of experiments and agents

Plants

Seed stocks	Nicotiana tabacum cv. Petit Havana, kept as lab stock for 20 years.
Novel plant genotypes	Previously unpublished transplastomic lines were generated by biolistic transformation via homologous recombination and spectinomycin selection as described in the Methods. All vectors used for transformation are described in detail in the Supplementary Information.
Authentication	Homoplasmy of all previously unpublished transplastomic lines was confirmed by RFLP analyses that are shown in the Supplementary Information.
