# Supplementary material for: Assembly-dependent translational feedback regulation of photosynthetic proteins in land plants
Source: Nat Plants. 2025 Aug 18;11(9):1920–38. doi: 10.1038/s41477-025-02074-x (PMC12449265; doi:10.1038/s41477-025-02074-x)

Source data for Fig. S6

Replicate II

Coomassie

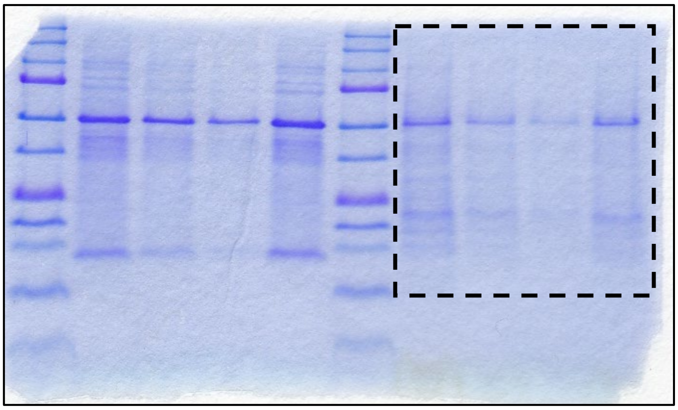

Pulse Labeling

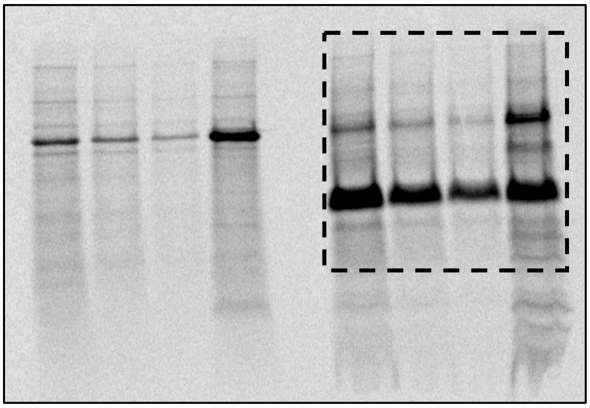

Replicate III

Coomassie

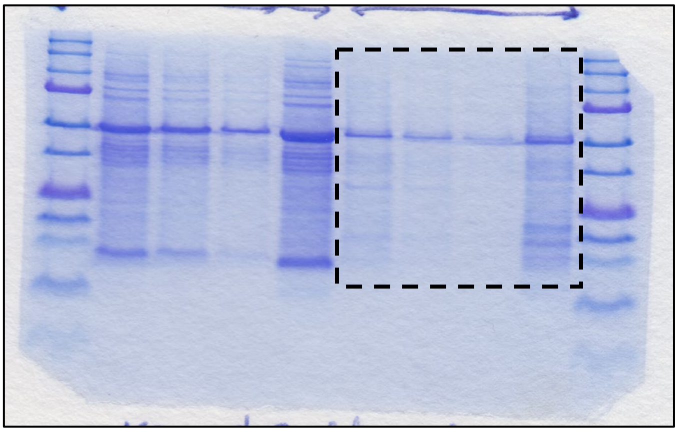

Pulse Labeling

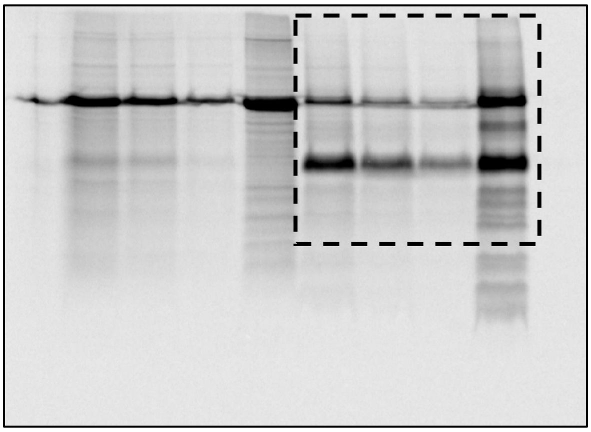

Supplement: Supplementary file 4 — Source data for supplementary figures. [file 41477_2025_2074_MOESM4_ESM.zip › Source_Data_for_Supplemental_Figures/Source Data Fig.S6.pdf]
