# Supplementary material for: Assembly-dependent translational feedback regulation of photosynthetic proteins in land plants
Source: Nat Plants. 2025 Aug 18;11(9):1920–38. doi: 10.1038/s41477-025-02074-x (PMC12449265; doi:10.1038/s41477-025-02074-x)

Source data for Fig. S9A

PetA

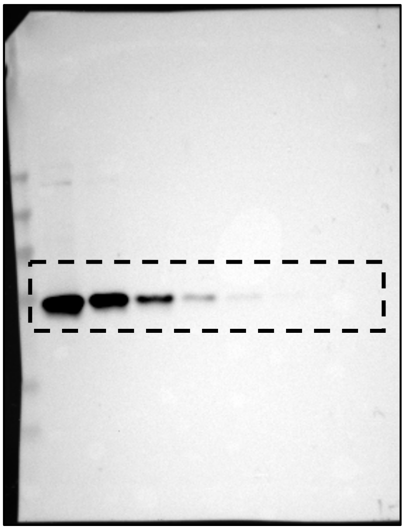

Ponceau

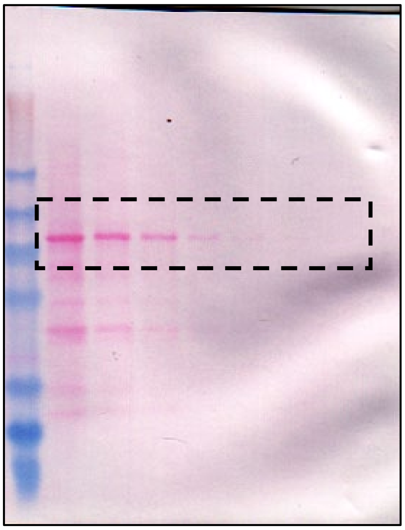

Source data for Fig. S9B

Replicate II
5 µg total protein (100%)

PetA

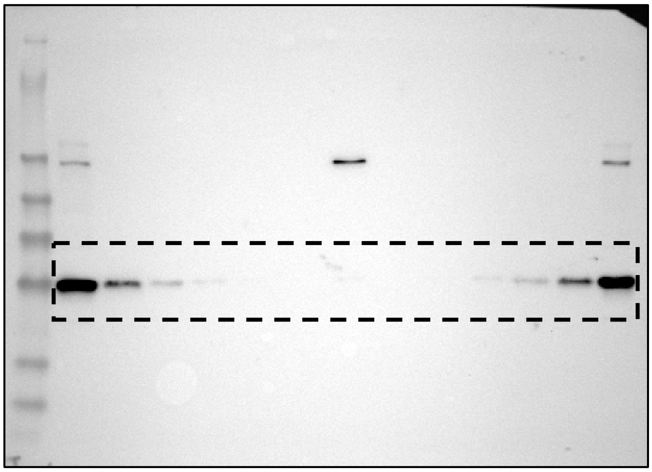

PetB

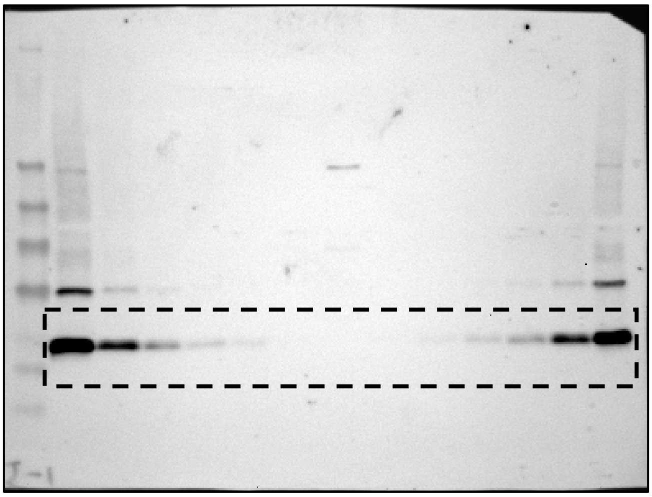

Ponceau

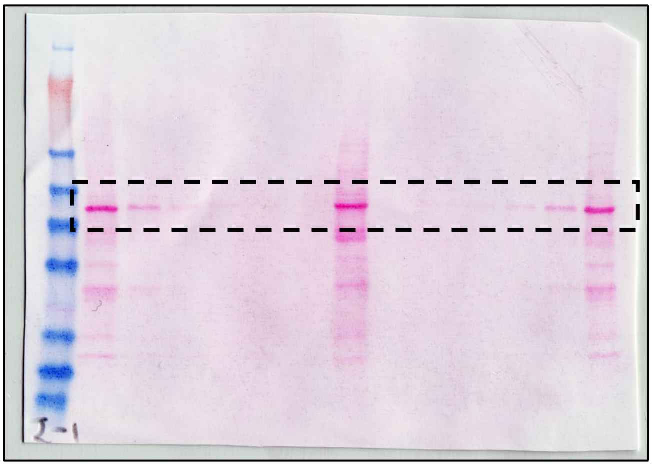

Source data for Fig. S9B

Replicate II
25 µg total protein (500%)

PetA

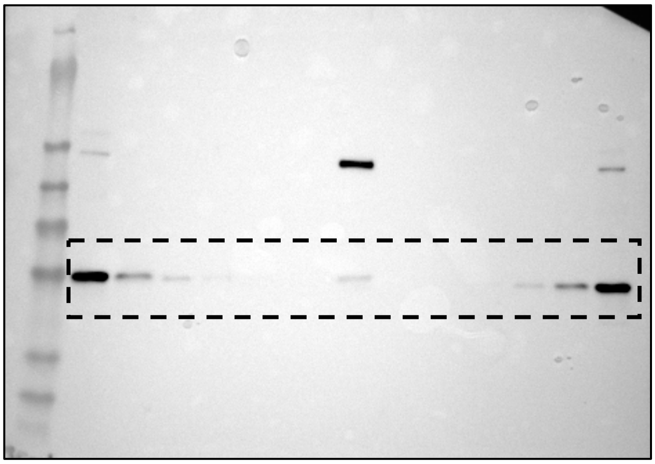

PetB

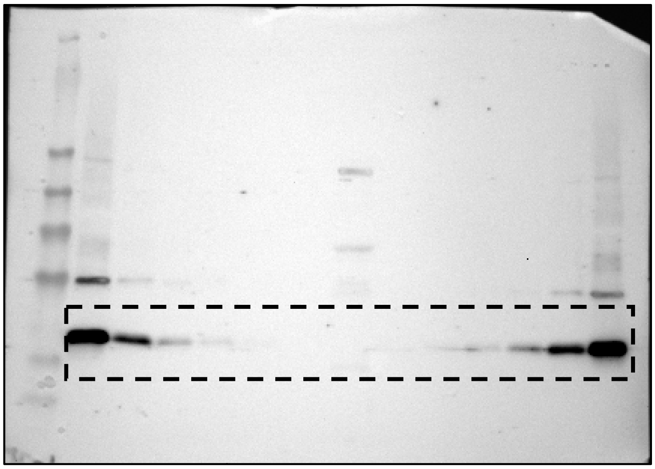

Ponceau

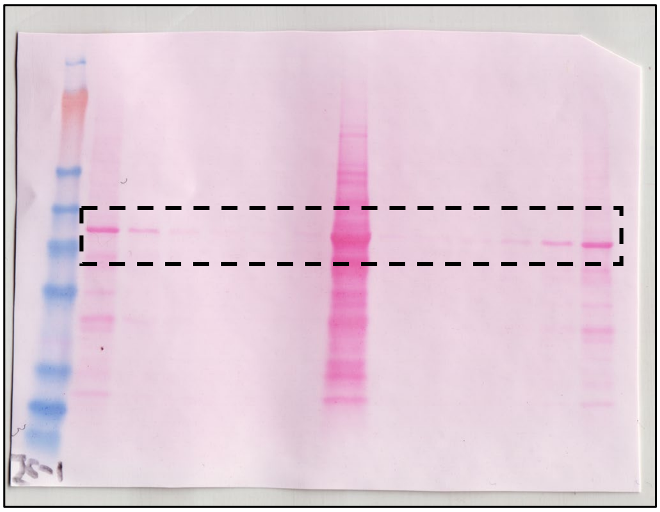

Supplement: Supplementary file 4 — Source data for supplementary figures. [file 41477_2025_2074_MOESM4_ESM.zip › Source_Data_for_Supplemental_Figures/Source Data Fig.S9.pdf]
