# Supplementary material for: Assembly-dependent translational feedback regulation of photosynthetic proteins in land plants
Source: Nat Plants. 2025 Aug 18;11(9):1920–38. doi: 10.1038/s41477-025-02074-x (PMC12449265; doi:10.1038/s41477-025-02074-x)

Source data for Fig. 1D
Replicate I

Coomassie

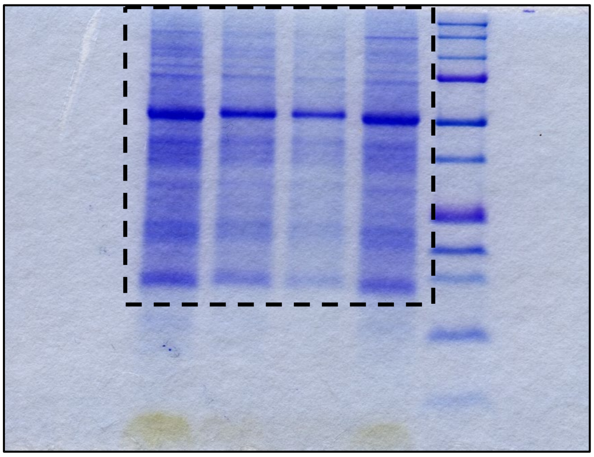

Pulse Labeling

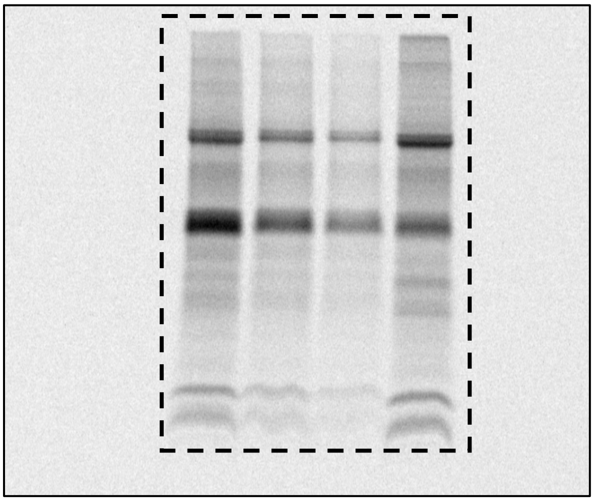

Source data for Fig. 1E

Total Band Volume

	Replicate 1	Replicate 2	Replicate 3
Control 100%	20090316	20090316	5578368
Control 50%	15119689	15119689	4046328
Control 25%	10293545	10293545	2637366
KD-psbD	24135672	24135672	3177471

Backround excluding PsbA and RbcL bands

	Replicate 1					Replicate 2					Replicate 3				
	lower part	middle part	upper part	SUM	Normalization factor	lower part	middle part	upper part	SUM	Normalization factor	lower part	middle part	upper part	SUM	Normalization factor
Control 100%	919944	176824	358344	1455112	0.46	1837401	727605	1087233	3652239	0.22	1415543	124215	419750	1959508	0.44
Control 50%	294768	100316	272548	667632	0.21	1226234	500490	641994	2368718	0.14	390960	139944	446904	977808	0.22
Control 25%	285336	99634	174363	559333	0.18	850967	447359	472350	1770676	0.11	546203	111972	165927	824102	0.18
KD-psbD	1687249	859940	593160	3140349	1	7874658	5506974	3204366	16585998	1	3410852	120912	929363	4461127	1

Adjusted to the Background

	Replicate 1	Replicate 2	Replicate 3
Control 100%	25107236	91236620	12700029
Control 50%	17473539	52934780	9230433
Control 25%	7453793	24105019	3569226
KD-psbD	8582318	24135672	3177471

Control set to 1

	Replicate 1	Replicate 2	Replicate 3	Average	std deviation
Control 100%	1	1	1	1	0
Control 50%	0.70	0.58	0.73	0.67	0.08
Control 25%	0.30	0.26	0.28	0.28	0.02
KD-psbD	0.34	0.26	0.25	0.29	0.05

Source data for Fig. 1H

Replicate I

Methylene Blue

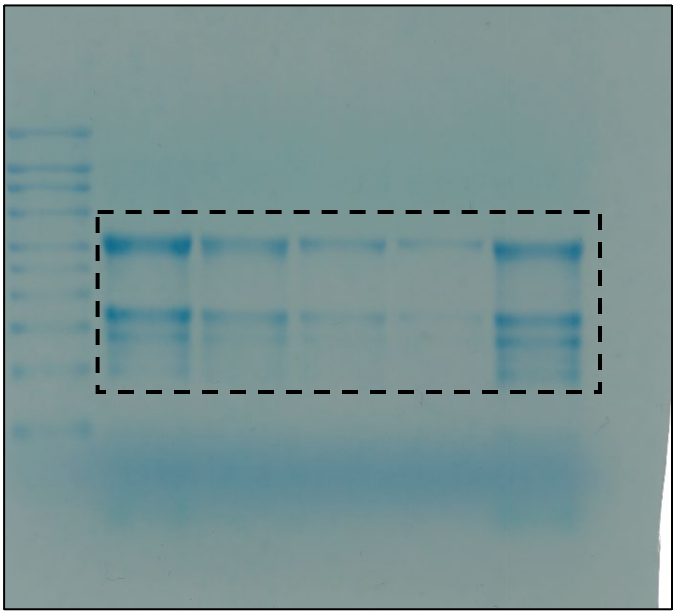

Northern Blot

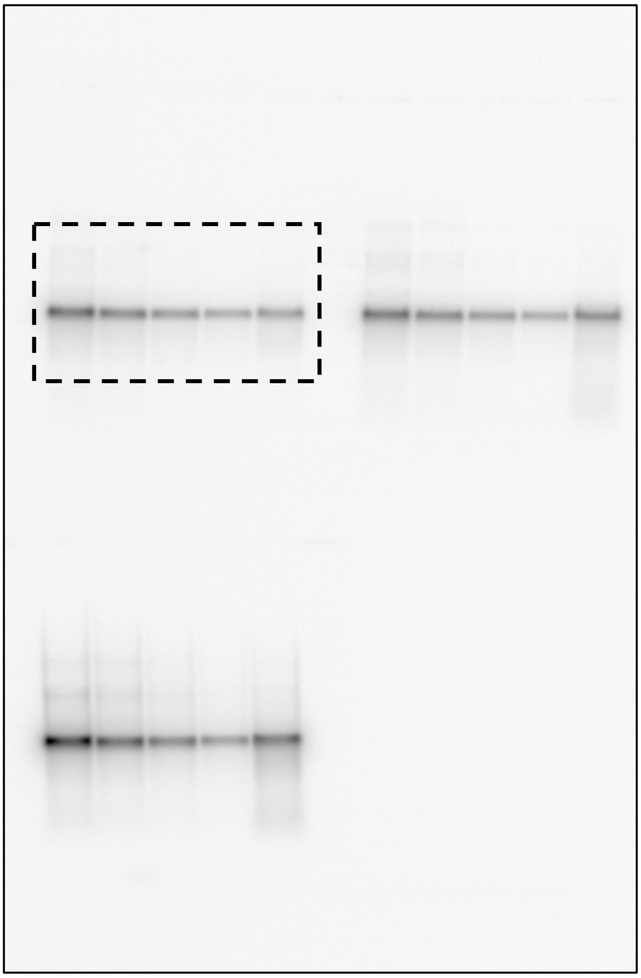

Supplement: Supplementary file 5 — Statistical source data and unprocessed pulse labelling and northern blots. [file 41477_2025_2074_MOESM5_ESM.pdf]
