# Supplementary material for: Assembly-dependent translational feedback regulation of photosynthetic proteins in land plants
Source: Nat Plants. 2025 Aug 18;11(9):1920–38. doi: 10.1038/s41477-025-02074-x (PMC12449265; doi:10.1038/s41477-025-02074-x)

Source data for Fig. 3F

Replicate I
5 µg total protein (100%)

PetA

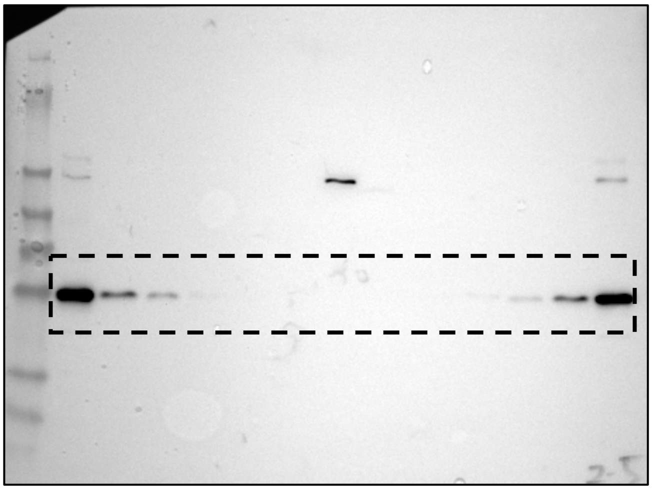

PetB

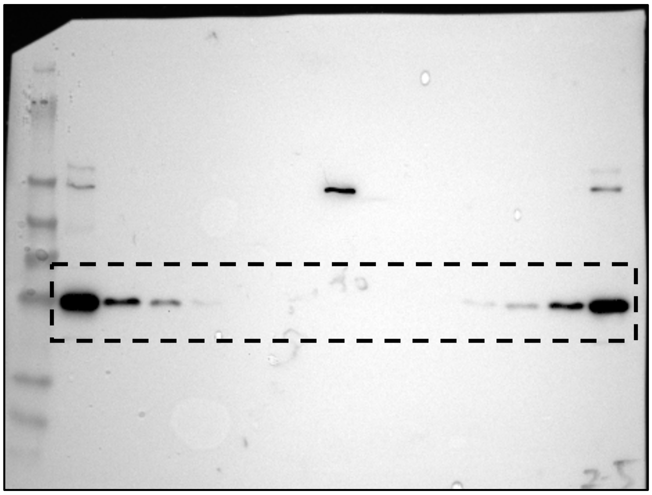

Ponceau

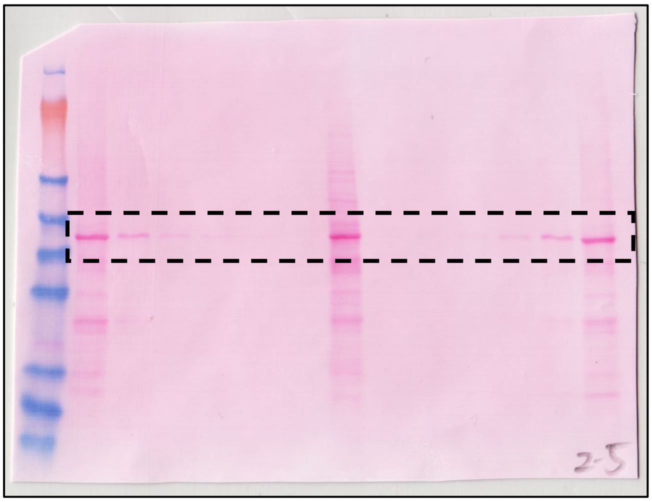

Source data for Fig. 3F

Replicate I
25 µg total protein (500%)

PetA

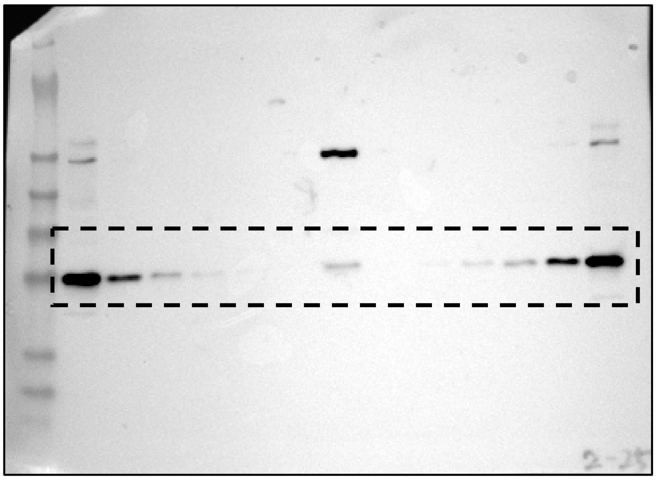

PetB

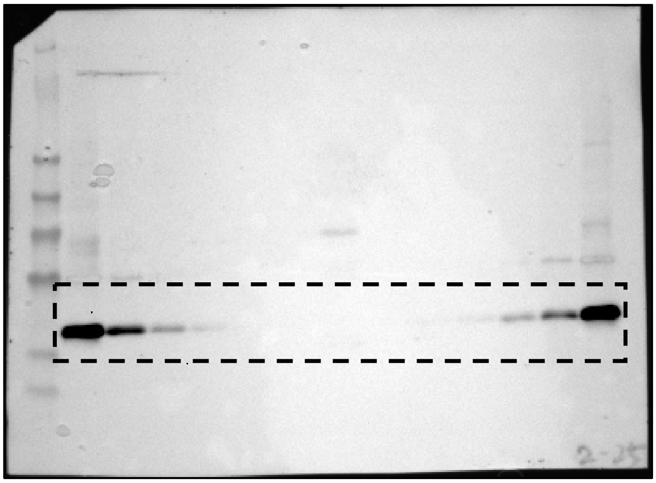

Ponceau

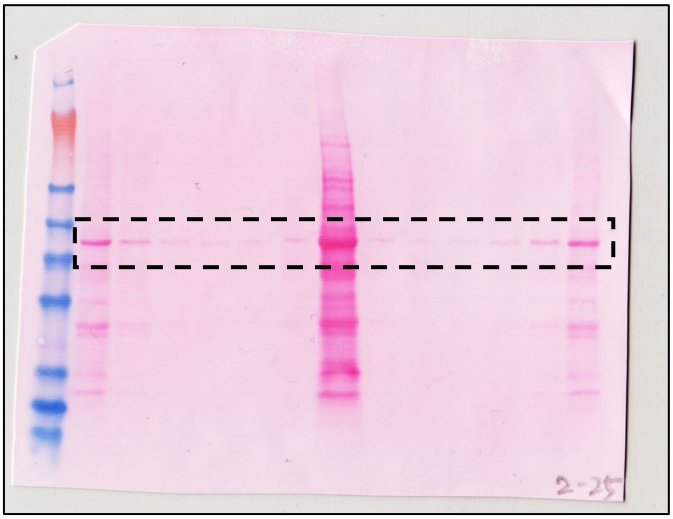

Supplement: Supplementary file 6 — Unprocessed western blots. [file 41477_2025_2074_MOESM6_ESM.pdf]
