# Supplementary material for: Assembly-dependent translational feedback regulation of photosynthetic proteins in land plants
Source: Nat Plants. 2025 Aug 18;11(9):1920–38. doi: 10.1038/s41477-025-02074-x (PMC12449265; doi:10.1038/s41477-025-02074-x)

Source data for Fig. 6F

PsbD

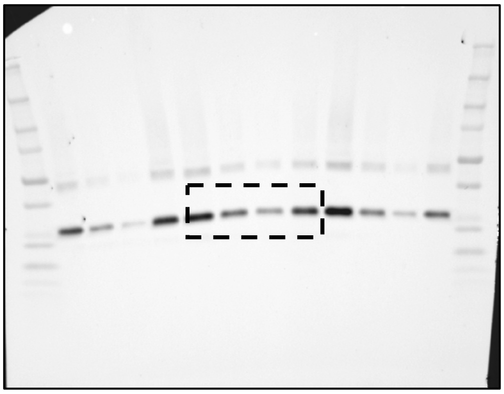

PsaD

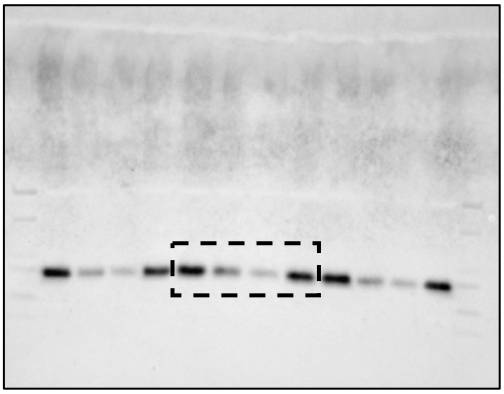

PetB

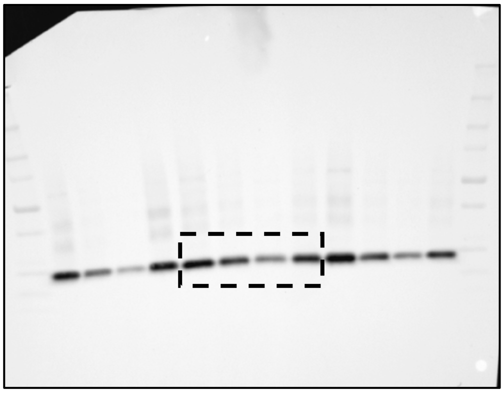

AtpB

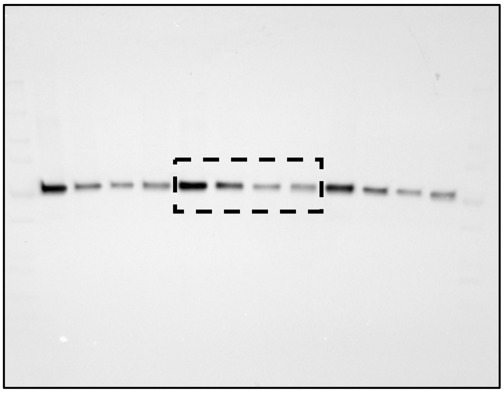

Ponceau

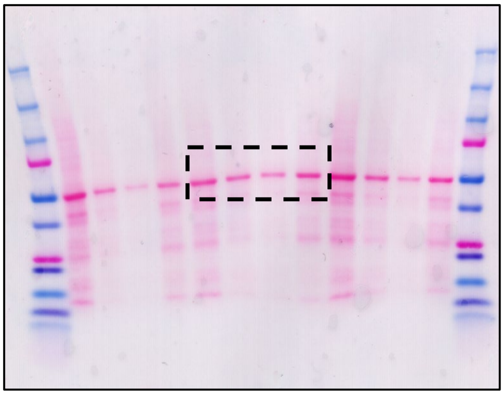

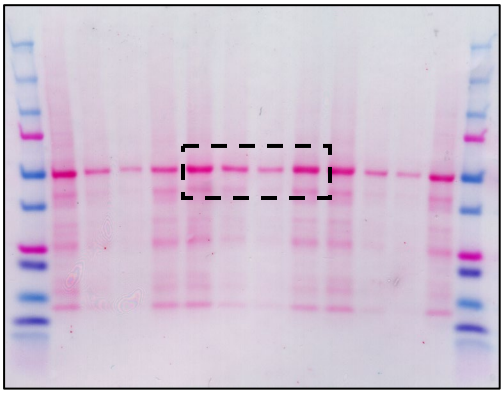

Source data for Fig. 6H

Methylene Blue

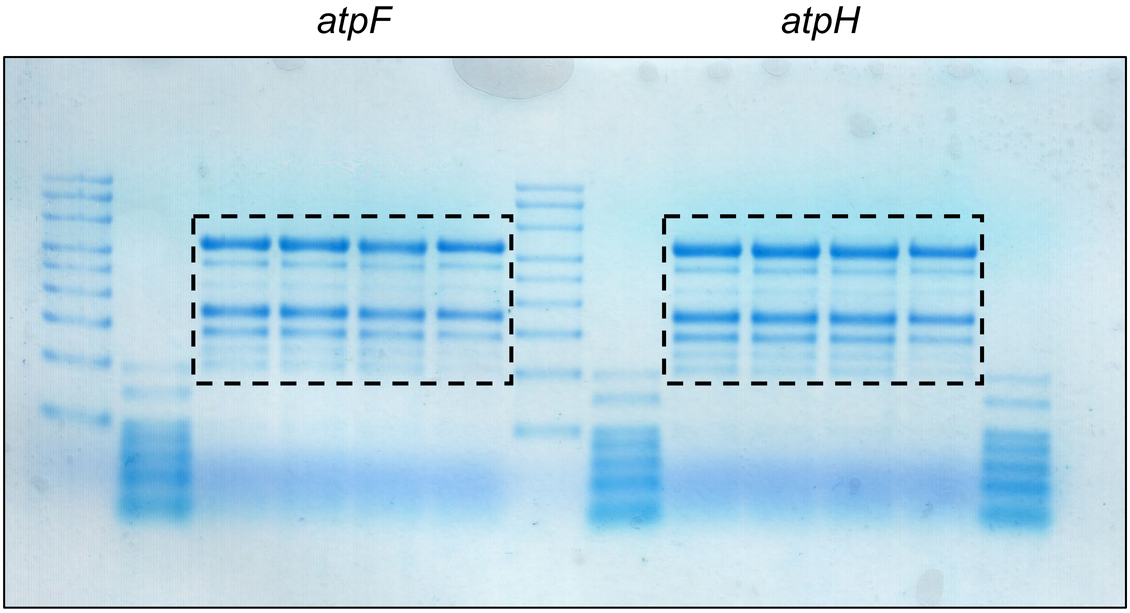

Northern Blot *atpF*

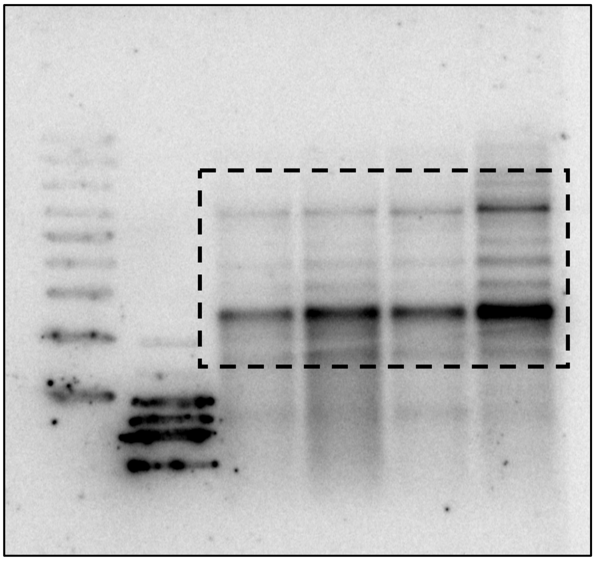

Northern Blot *atpH*

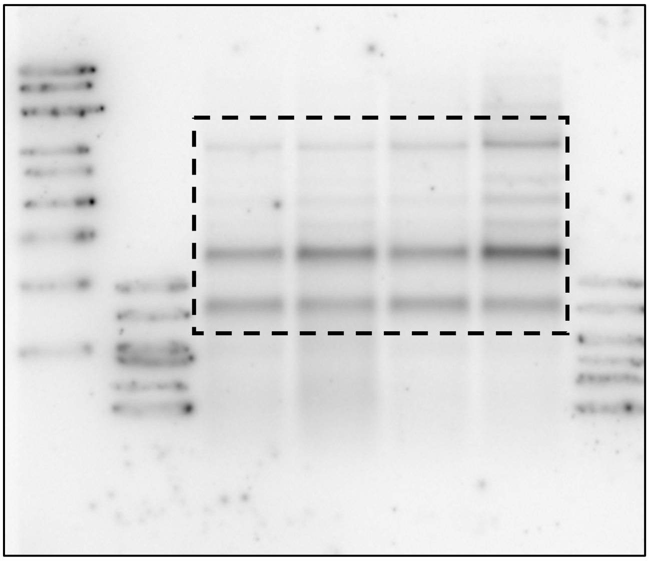

Supplement: Supplementary file 8 — Unprocessed western and northern blots. [file 41477_2025_2074_MOESM8_ESM.pdf]
