# Supplementary material for: Assembly-dependent translational feedback regulation of photosynthetic proteins in land plants
Source: Nat Plants. 2025 Aug 18;11(9):1920–38. doi: 10.1038/s41477-025-02074-x (PMC12449265; doi:10.1038/s41477-025-02074-x)
Supplement: Supplementary file 9 — Statistical source data and unprocessed pulse labelling blots. [file 41477_2025_2074_MOESM9_ESM.pdf]

Source data for Extended Fig. 1C

Replicate I

Coomassie

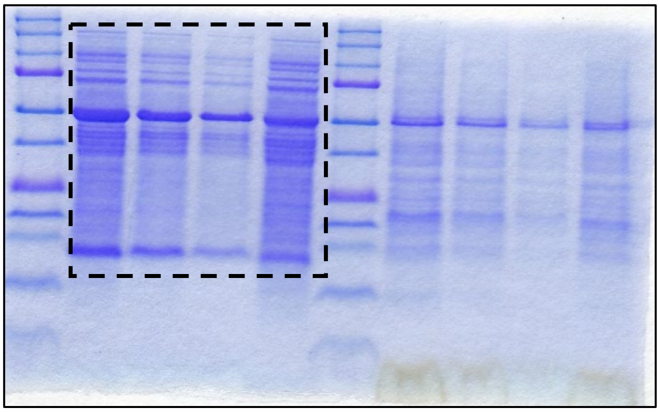

Pulse Labeling

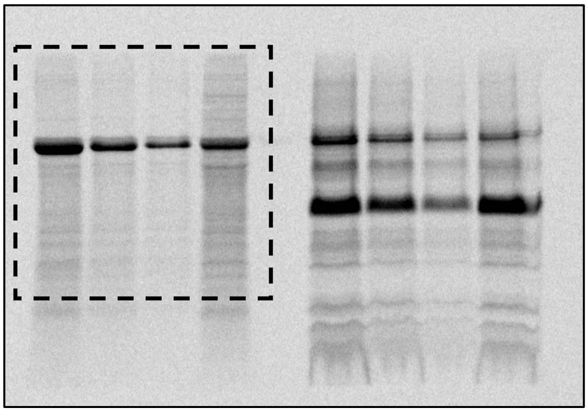

Replicate II

Coomassie

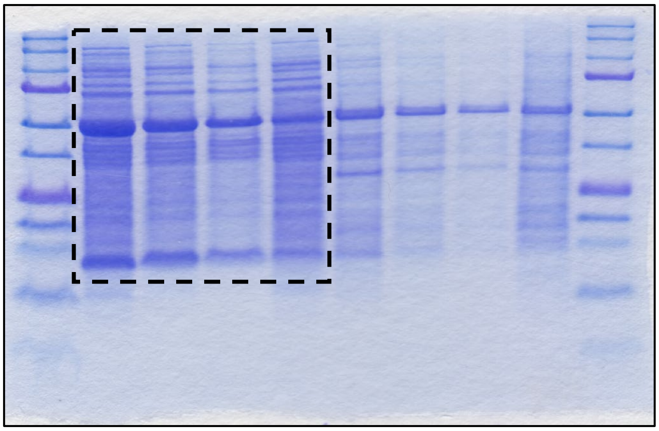

Pulse Labeling

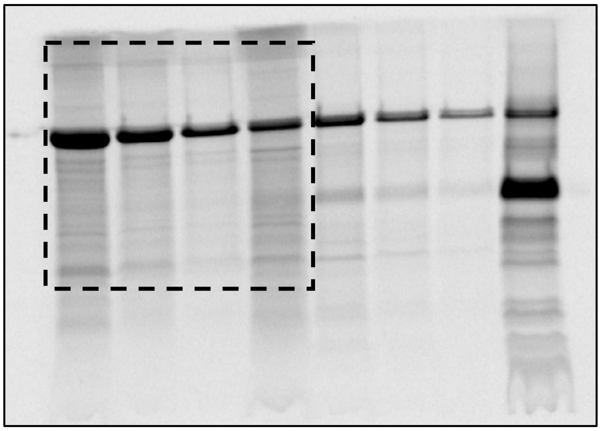

Source data for Extended Fig. 1D

Total Band Volume

	Replicate 1	Replicate 2
Control 100%	7521336	31631950
Control 50%	4752510	20877780
Control 25%	2835831	14194048
KD-psbD	3696350	10644561

Backround excluding RbcL band

	Replicate 1				Replicate 2			
	lower part	upper part	SUM	Normalization factor	lower part	upper part	SUM	Normalization factor
Control 100%	1171212	316455	1487667	1.00	5935440	2350530	8285970	1.00
Control 50%	553770	214620	768390	0.52	2708046	1820280	4528326	0.55
Control 25%	487347	183195	670542	0.45	1789828	1071340	2861168	0.35
KD-psbD	1629390	483770	2113160	1.42	3457790	3395352	6853142	0.83

Adjusted to the Background

	Replicate 1	Replicate 2
Control 100%	7521336	31631950
Control 50%	4600627	19101171
Control 25%	1572896	10276525
KD-psbD	2602235	12870084

Control set to 1

	Replicate 1	Replicate 2	Average
Control 100%	1	1	1
Control 50%	0.61	0.60	0.61
Control 25%	0.21	0.32	0.27
KD-psbD	0.35	0.41	0.38
