# Supplementary material for: Assembly-dependent translational feedback regulation of photosynthetic proteins in land plants
Source: Nat Plants. 2025 Aug 18;11(9):1920–38. doi: 10.1038/s41477-025-02074-x (PMC12449265; doi:10.1038/s41477-025-02074-x)

Source data for Extended Fig. 4A

PsbD

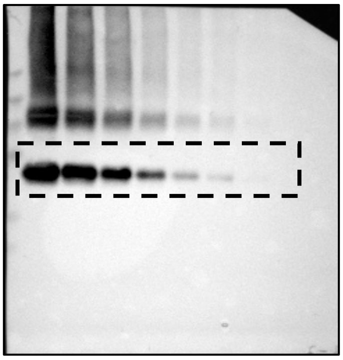

PsbB

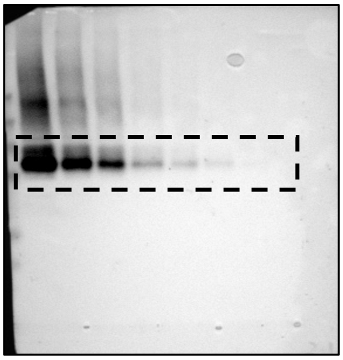

Ponceau

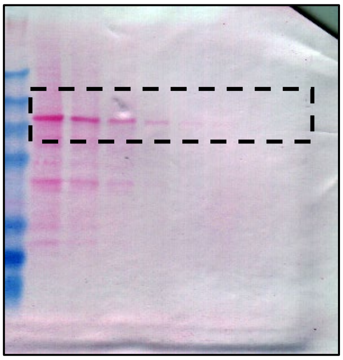

Ponceau

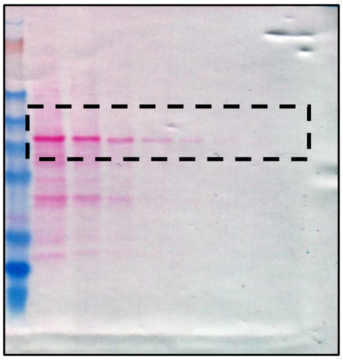

PsbC

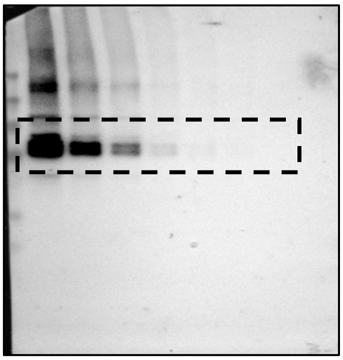

PsbA

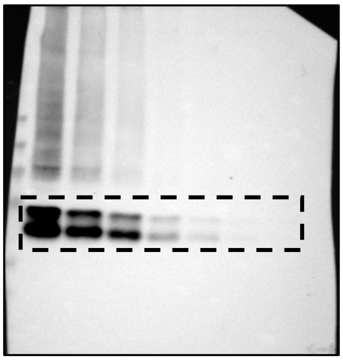

Ponceau

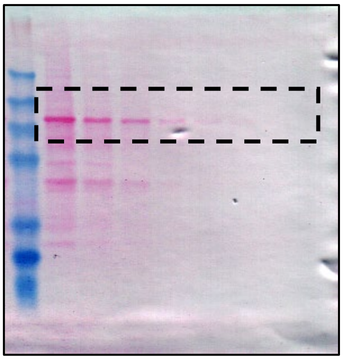

Ponceau

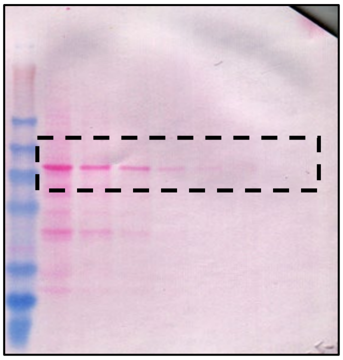

PsbH

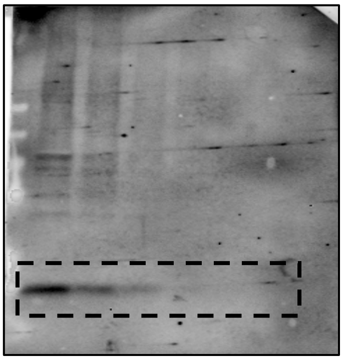

Ponceau

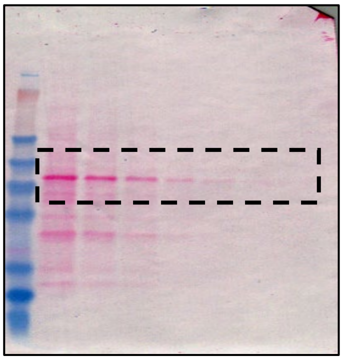

Source data for Extended Fig. 4B

PsbD

Rep I Rep II Rep III

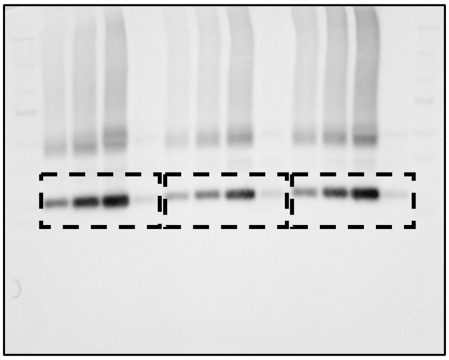

PsbH

Rep I Rep II Rep III

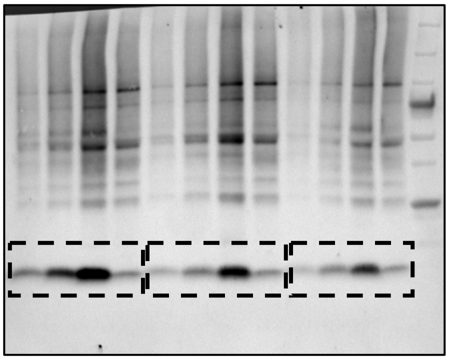

PsbC

Rep I Rep II Rep III

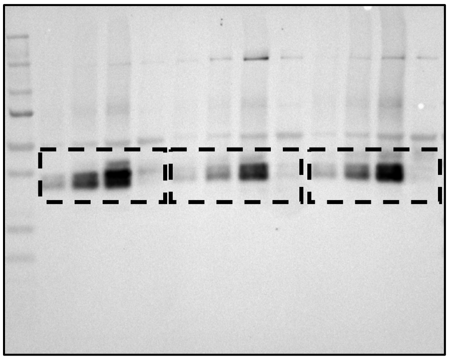

Ponceau

Rep I Rep II Rep III

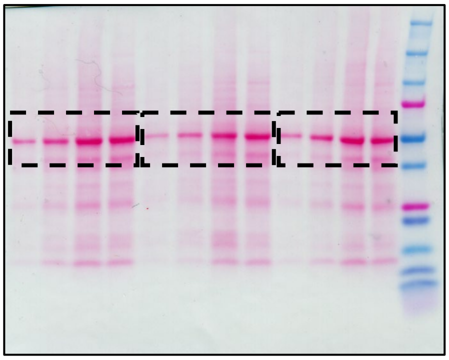

PsbB

Rep I Rep II Rep III

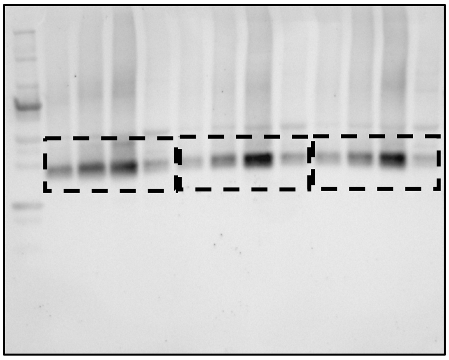

PsbA

Rep I Rep II Rep III

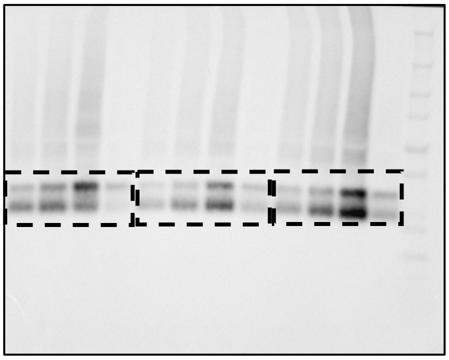

Ponceau

Rep I Rep II Rep III

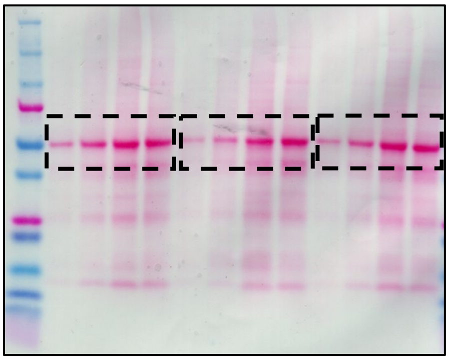

Ponceau

Rep I Rep II Rep III

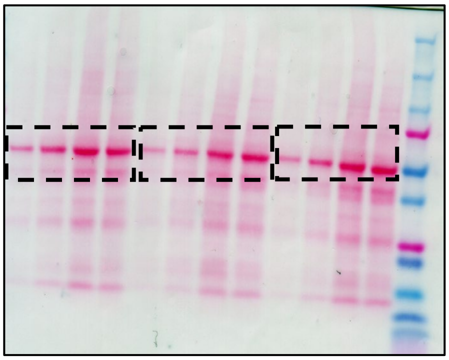

Source data for Extended Fig. 4C

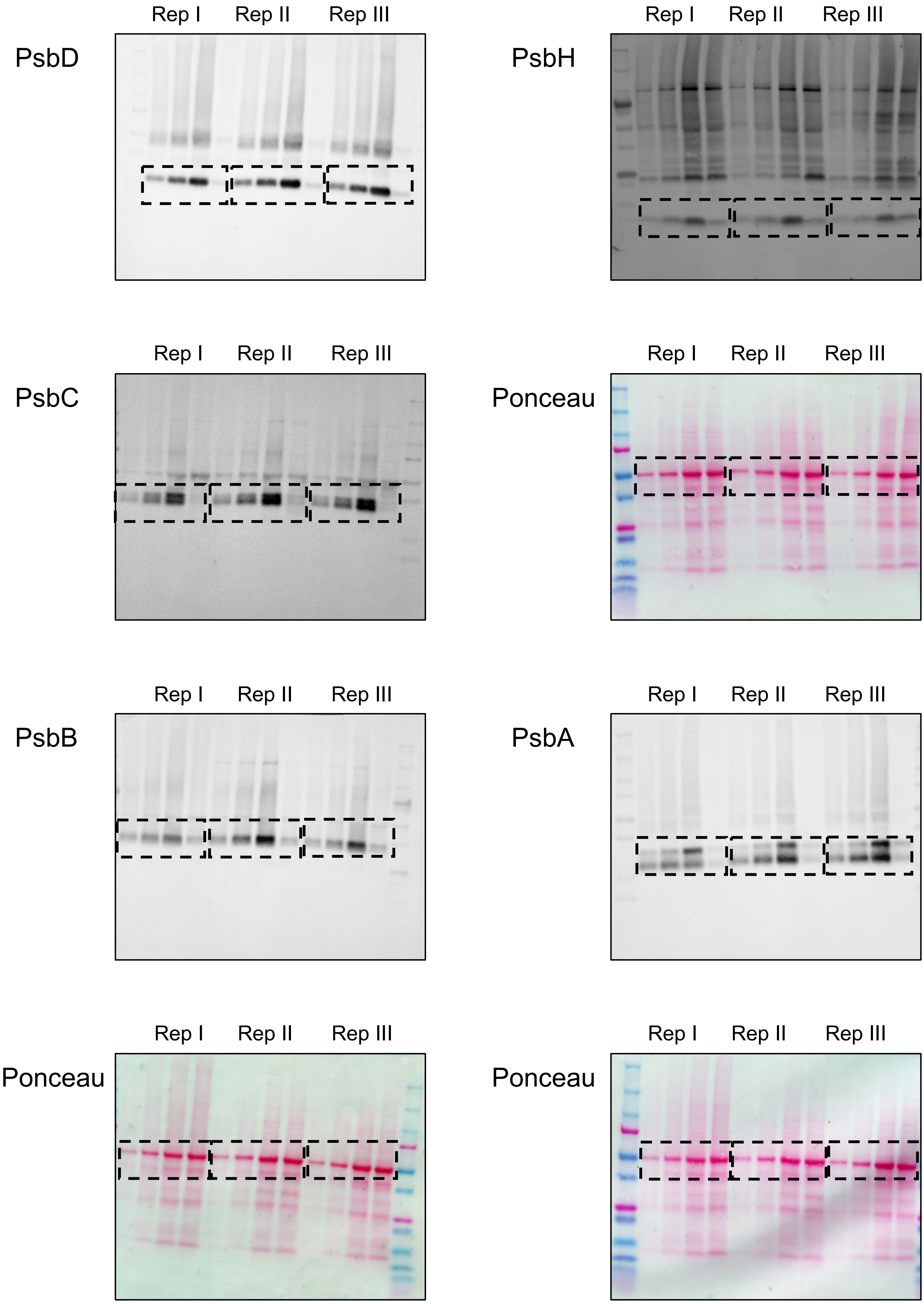

Source data for Extended Fig. 4D

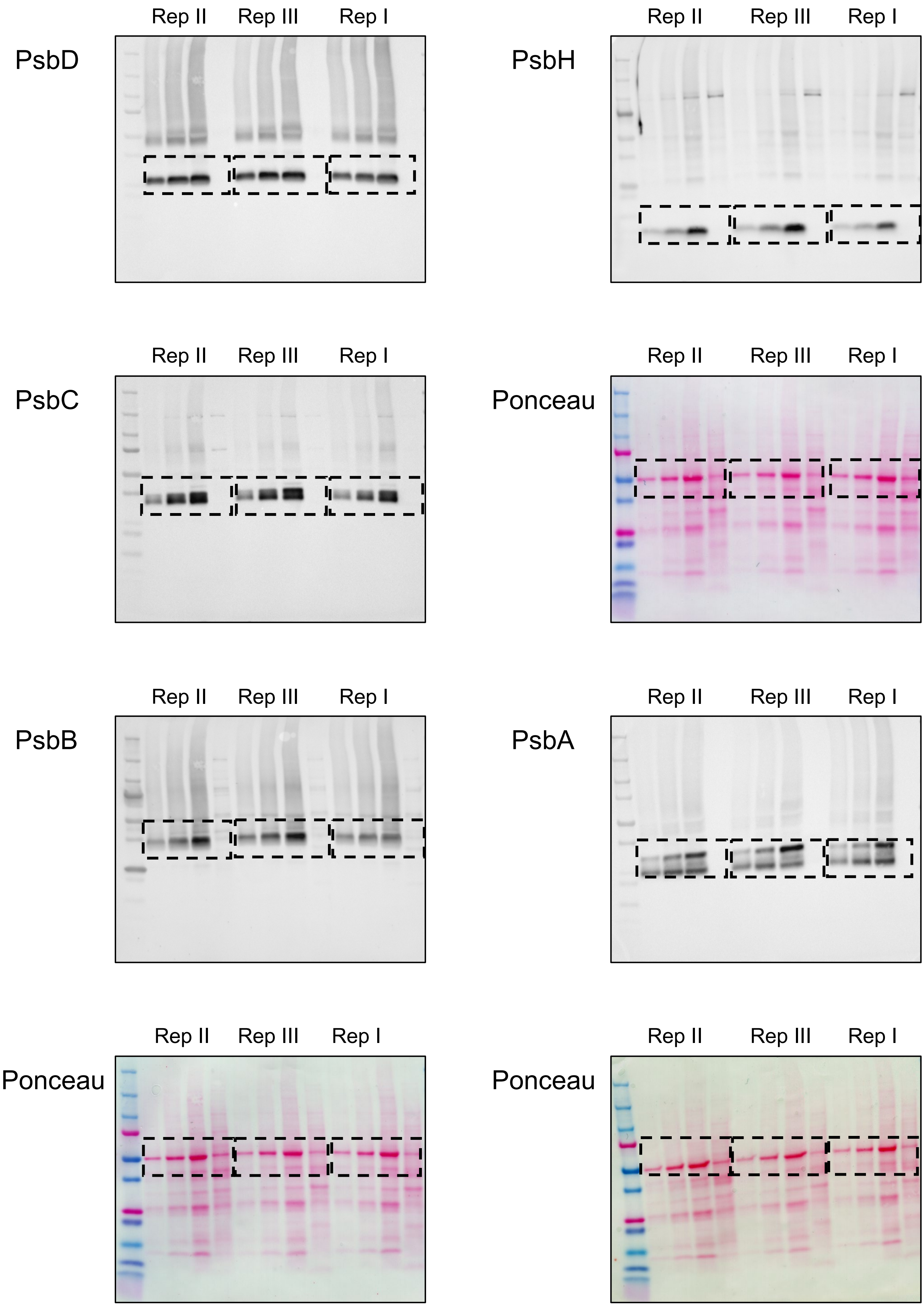

Source data for Extended Fig. 4E

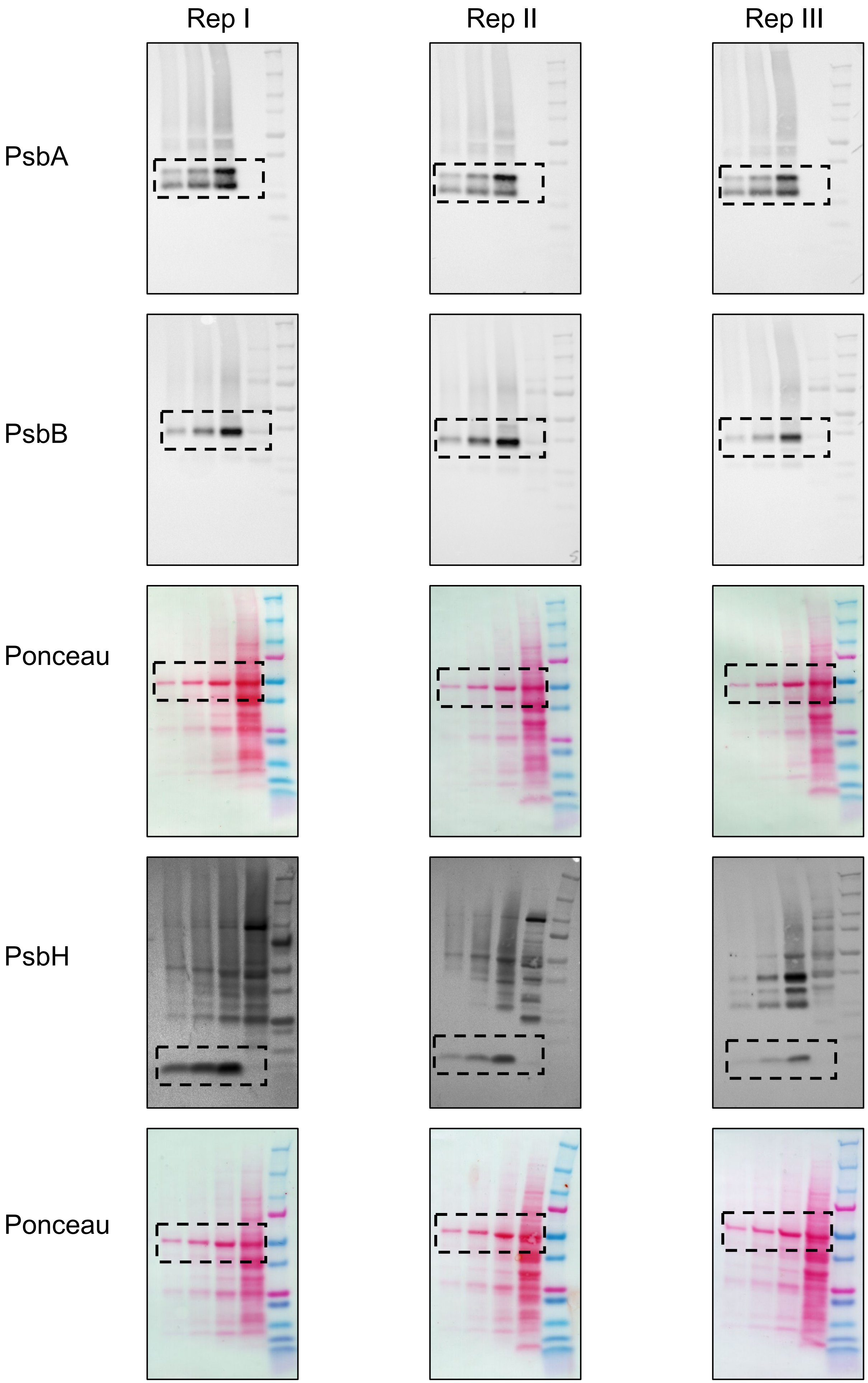

Source data for Extended Fig. 4F

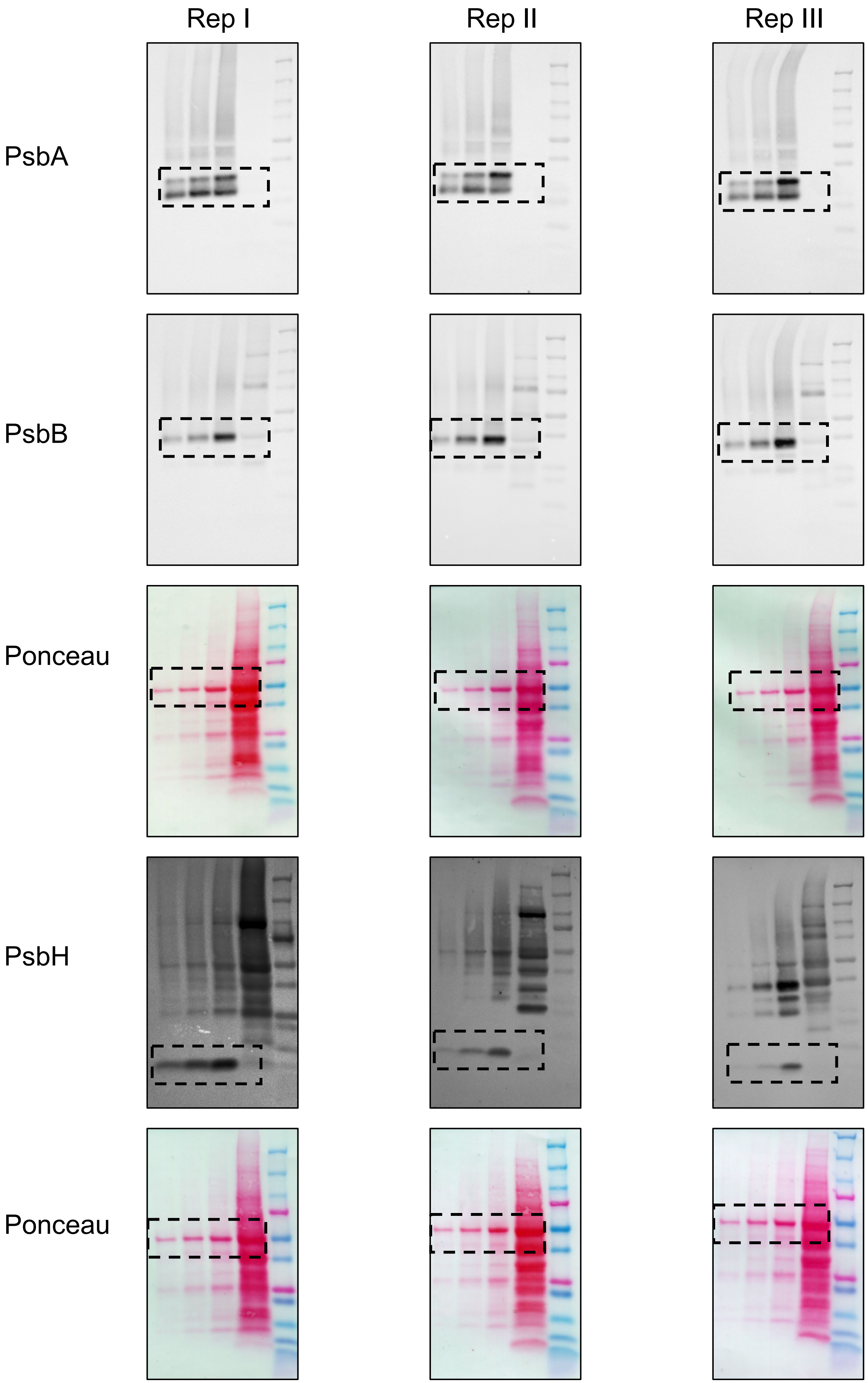

Supplement: Supplementary file 10 — Unprocessed western blots. [file 41477_2025_2074_MOESM10_ESM.pdf]
