# Supplementary material for: Assembly-dependent translational feedback regulation of photosynthetic proteins in land plants
Source: Nat Plants. 2025 Aug 18;11(9):1920–38. doi: 10.1038/s41477-025-02074-x (PMC12449265; doi:10.1038/s41477-025-02074-x)

Source data for Extended Fig. 6D

Replicate I

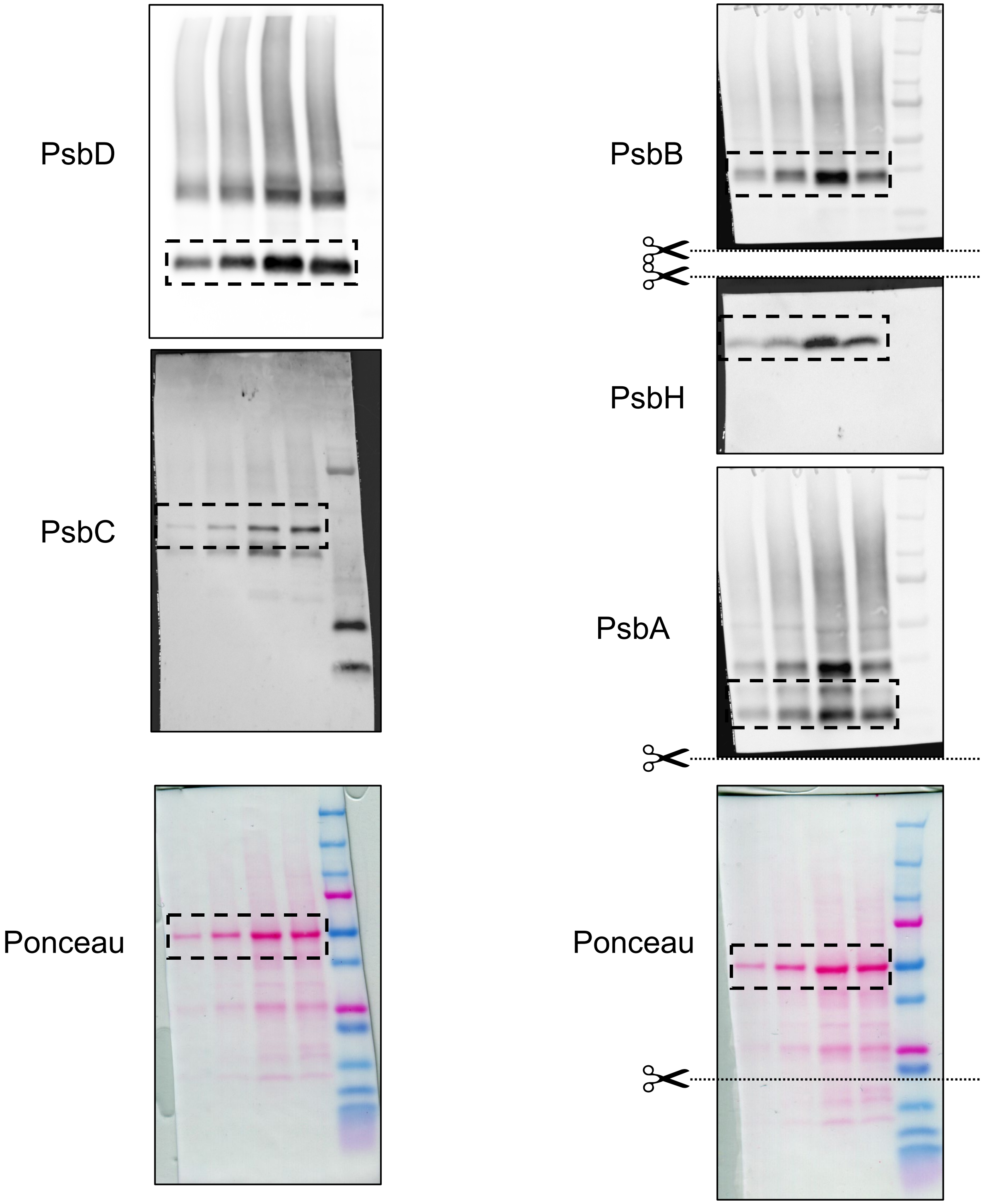

Source data for Extended Fig. 6D

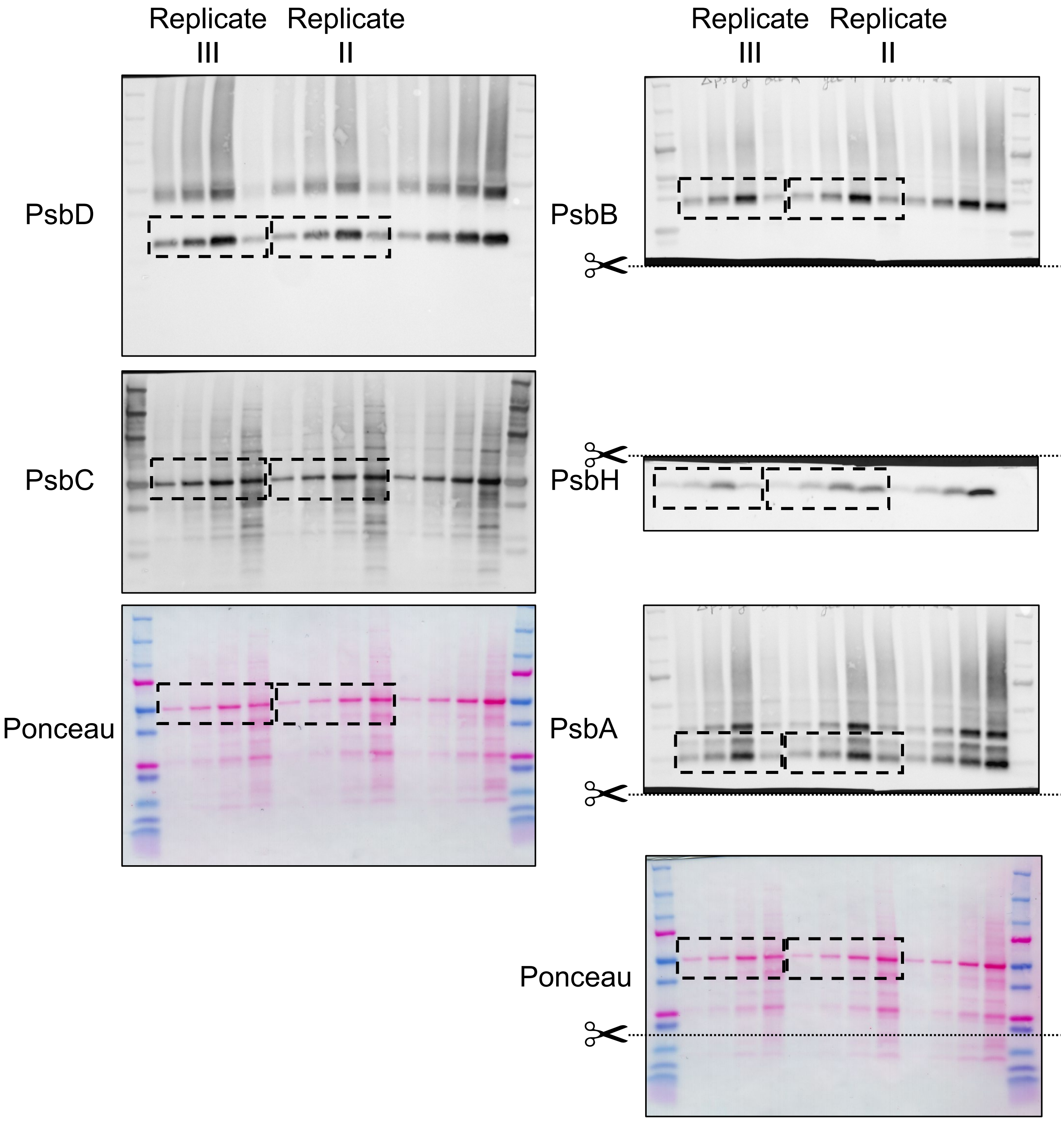

Supplement: Supplementary file 11 — Unprocessed western blots. [file 41477_2025_2074_MOESM11_ESM.pdf]
